# Seven-Membered Cyclic Diamidoalumanyls of Heavier Alkali Metals: Structures and C–H Activation of Arenes

**DOI:** 10.1021/acs.organomet.3c00323

**Published:** 2023-09-09

**Authors:** Han-Ying Liu, Michael S. Hill, Mary F. Mahon, Claire L. McMullin, Ryan J. Schwamm

**Affiliations:** Department of Chemistry, University of Bath, Claverton Down, Bath BA2 7AY, U.K.

## Abstract

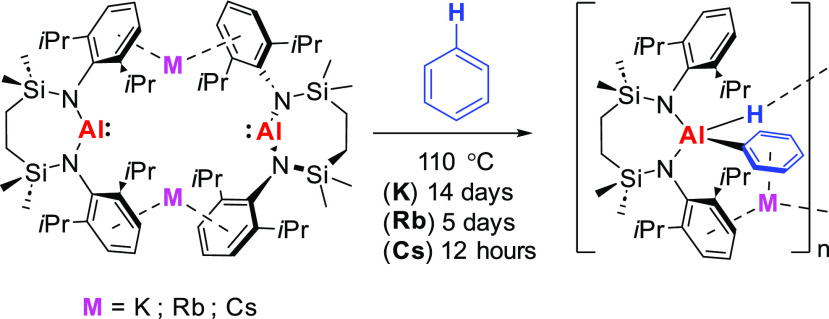

Like the previously reported potassium-based system, rubidium and cesium reduction of [{SiN^Dipp^}AlI] ({SiN^Dipp^} = {CH_2_SiMe_2_NDipp}_2_) with the heavier alkali metals [M = Rb and Cs] provides dimeric group 1 alumanyl derivatives, [{SiN^Dipp^}AlM]_2_. In contrast, similar treatment with sodium results in over-reduction and incorporation of a formal equivalent of [{SiN^Dipp^}Na_2_] into the resultant sodium alumanyl species. The dimeric K, Rb, and Cs compounds display a variable efficacy toward the C–H oxidative addition of arene C–H bonds at elevated temperatures (Cs > Rb > K, 110 °C) to yield (hydrido)(organo)aluminate species. Consistent with the synthetic experimental observations, computational (DFT) assessment of the benzene C–H activation indicates that rate-determining attack of the Al(I) nucleophile within the dimeric species is facilitated by π-engagement of the arene with the electrophilic M^+^ cation, which becomes increasingly favorable as group 1 is descended.

## Introduction

**Figure 1:**
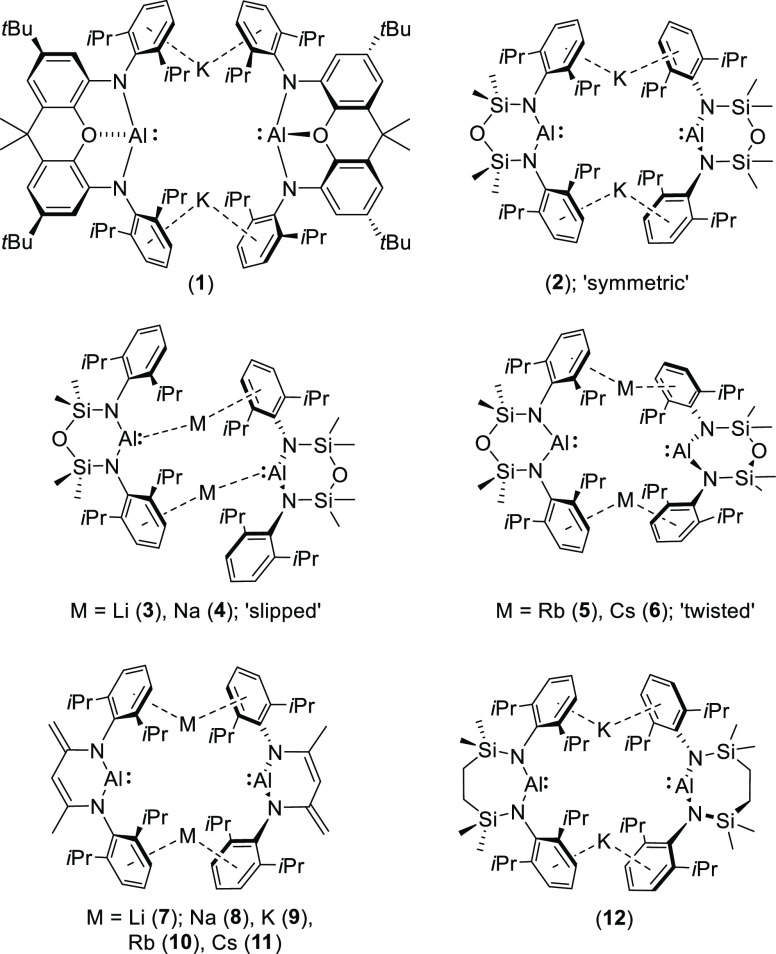
Exemplary diamidoalumanyl derivatives: compounds **1–12**.

In the less than half decade since Aldridge, Goicoechea, and co-workers’ report of the dimeric xanthene-based species [K{Al (_xanth_NON)}]_2_ (**1**, _xanth_NON = [4,5-(NDipp)_2_-2,7-*t*-Bu_2_-9,9-Me_2_-xanthene]^2–^), where Dipp = 2,6-*i*-Pr_2_C_6_H_3_; Figure 1),^1^ the synthesis and reactivity of alumanyl anions have developed into a significant subfield of main group element chemistry.^2–4^ The ability of compound **1** to act as a potent two-electron reductant or as a source of nucleophilic aluminum has provided the impetus for a rich reaction chemistry encompassing both the activation of robust small molecules^1,5–23^ and the isolation and study of a variety of unprecedented metal-to-Al bonds.^24–35^ Like compound **1**, the majority of subsequently reported alumanyl compounds (e.g., [{(NON)AlK]_2_ (NON = O(SiMe_2_NDipp)_2_; **2**) have employed elemental potassium to effect reduction to the formal Al(I) oxidation state. As well as providing the necessary charge balance, the K^+^ cation has been shown to deliver a variety of dimeric, monomeric, or charge-separated structures and to leverage contrasting chemical behaviors that are dependent either on the basicity of the solvent employed or through the introduction of a crown ether or cryptand cocomplexant.^5,7^

Despite the initial pre-eminence of potassium alumanyl species, more recent attention has turned to derivatives comprising alternative lighter (Li and Na^12,26,35,36^) or heavier (Rb and Cs^36,37^) counter cations. Coles, Mulvey, and co-workers’ extension of the NON-derived species to a contiguous series of contact pair alumanyls comprising all of the available alkali metals [{(NON)Al}M]_2_ M = Li (**3**),^12^ Na (**4**),^12^ K (**2**),^9^ Rb (**5**),^37^ and Cs (**6**)^37^ has highlighted a structural transition from so-termed “slipped” (**3** and **4**) through “symmetric” (**2**) to “twisted” (**5** and **6**) modes of *N*-aryl···M-based dimerization.^3^ While this solid-state feature may be attributed to the differing size and polarizability of the group 1 cations, of perhaps greater significance is the contrasting reactivity of **2–6** toward benzene solvent. The initial study of compound **1** disclosed that a C–H bond of benzene underwent oxidative addition to afford the potassium (hydrido)(phenyl)aluminate, [K{Al(H)(C_6_H_5_)(_xanth_NON)}]_2_, over 4 days at 57 °C.^1^ Although several other subsequently reported potassium alumanyls have displayed similar or related C(sp^2^)–H reactivity,^6,15,22,38^ only the cesium derivative (**6**) among Coles’ NON-supported derivatives, **2–6**, provided comparable behavior, yielding [Cs{Al(H)(C_6_H_5_)(NON)}] after 5 days of heating at 80 °C.^37^ Density functional theory (DFT) calculations attributed this limitation to a likely example of synergistic alkali metal mediation (AMM),^39^ in which the heavier alkali metal exerts a greater (i.e., more exergonic) influence over the formation of an aluminum nucleophile-induced Meisenheimer intermediate prior to the C–H activation transition state.^37^ In an even more recent advance, Harder and co-workers have devised synthetic routes to a further complete series of dimeric group 1 alumanyls, [{(BDI^Dipp^-H)Al}M]_2_ (BDI^Dipp^-H = [DippNC(Me)·C(H)C(=CH_2_)(NDipp)]^2–^; M = Li (**7**), Na (**8**), K (**9**), Rb (**10**), and Cs (**11**)].^36^ Although these compounds again adopt alkali-metal-dependent “slipped” (**7**, **8**) or “symmetric” (**9–11**) structures in the solid state, each of the potassium, rubidium, and cesium derivatives was shown to induce a similar, but 2-fold, *para*-C–H activation of benzene.

Prompted by an initial intention to elaborate alumanyl chemistry to the heavier elements of group 2, we have previously reported the seven-membered cyclic potassium diamidoalumanyl, [({SiN^Dipp^}Al)K]_2_ (**12**, {SiN^Dipp^} = {CH_2_SiMe_2_NDipp}_2_),^29^ which itself activates a methyl C(sp^3^)–H bond of the solvent when heated in toluene at 110 °C for 24 h to provide the benzylaluminum hydride product, [K{SiN^Dipp^}Al(H)(CH_2_Ph)].^17^ In this contribution, we report our efforts to extend the chemistry of the [{SiN^Dipp^}Al]^−^ alumanyl anion to a broader range of alkali metals and the reactivity of the resultant contact pair dimers toward the C–H bonds of arene solvents.

## Results and Discussion

### Synthesis of {SiN^Dipp^}-Ligated Sodium, Rubidium, and Cesium Alumanyls

Reduction of [{SiN^Dipp^}AlI] (**13**) either with a sodium mirror or with 5 wt.% Na/NaCl^40^ in hexane resulted in the complete consumption of the iodide reagent and the generation of a pale-yellow solution and gray suspension within 3 days at room temperature. Filtration and storage of a concentrated solution at −10 °C afforded compound **14** as a colorless crystalline powder in a good (>75%) yield. In contrast to the ^1^H and ^13^C nuclear magnetic resonance (NMR) spectra provided by compound **12**, which were diagnostic of a single chelated {SiN^Dipp^} environment and local *C*_2_ symmetry about aluminum, the corresponding spectra of **14** were more complex and, irrespective of the physical form of the group 1 reducing agent or the reaction stoichiometry, consistent with the presence of multiple dianilide ligand resonances. Notably, the specificity of the production of compound **14** was demonstrated by a further reaction performed between **13** and 5 wt.% Na/NaCl in C_6_D_6_ in a J Young NMR tube, monitoring of which evidenced the emergence of a ^1^H NMR spectrum over 5 days that was virtually identical to that provided by an isolated and crystallized sample.

**Figure 2:**
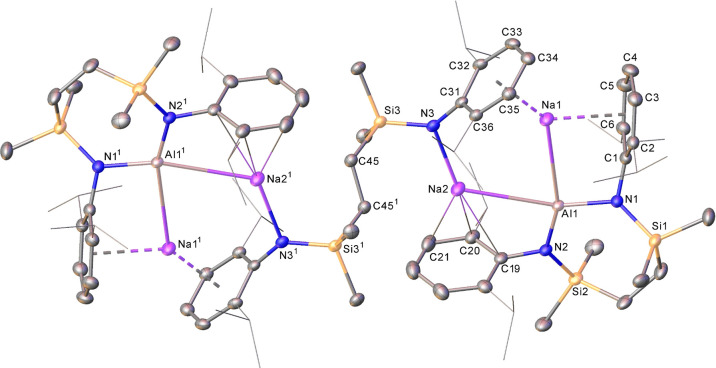
Displacement ellipsoid (30% probability) plot of the structure of compound **14**. For clarity, solvent has been omitted, and hydrogen atoms have been removed. Dipp *iso*-propyl substituents are also presented as wireframes for visual ease. Symmetry operation: ^1^1 – *x*, 1 – *y*, and 1 – *z*.

The origin of these observations was ultimately resolved by X-ray diffraction analysis of a single crystal of compound **14** (Figure 2 and Table 1), which may be considered a product of over-reduction of the target sodium alumanyl, [({SiN^Dipp^}Al)Na]_2_. Compound **14** is a heterobimetallic species, the asymmetric unit of which comprises half of the molecule, with the remainder generated via a crystallographic inversion center. The two symmetry-related aluminum atoms are *N*,*N′*-coordinated by still-chelated {SiN^Dipp^} ligands and display close contacts to two sodium cations [Al1–Na1 3.1251(10); Al1–Na2 3.2368(11) Å]. The sodium atoms in each of the resultant trimetallic units are bonded by polyhapto interactions with the Dipp substituents of the chelated {SiN^Dipp^} ligands but are differentiated by their binding to an additional diamide dianion that now adopts a {Na2-μ–κ^1^-*N*,μ–κ^1^-*N*′-Na2′} bridging mode. This latter bonding situation and the gross structure of **14** are, thus, strongly reminiscent of the recently reported and isomorphous sodium magnesiate [{(SiN^Dipp^)Mg(H)Na_2_}_2_{μ-(SiN^Dipp^)}] (**15**).^41^ Although charge balance in **15** was necessarily maintained by the trigonal encapsulation of a hydride by each MgNa_2_ array, the Al–N bond lengths to the {SiN^Dipp^} chelate [Al1–N1 1.8886(18); Al1–N2 1.8625(17) Å] in **14** are consistent with an assignment of the Al(I) oxidation state.^17,29^ This latter observation leads us to discount the possible presence of hydride anions in **14** and to continue to assign each ({SiN^Dipp^}Al) as alumanyl units with a high degree of confidence.

**Table 1:** Selected Bond Lengths (Å) and Angles (deg) of the Alkali Metal Alumanyls **14**, **16**, and **17**

	14^a^	16^b^	17^c^
Al1–N1	1.8886(18)	1.8866(15)	1.899(2)
Al1–N2	1.8625(17)	1.8880(16)	1.903(2)
Al2–N3		1.8960(16)	1.889(2)
Al2–N4		1.8989(16)	1.892(2)
M1-C1	2.912(2)	3.4783(17)	3.565(2)
M1-C2	2.853(2)	3.4805(18)	3.508(2)
M1-C3	2.930(3)	3.4302(19)	3.411(3)
M1-C4	3.082(3)	3.3889(19)	3.415(3)
M1-C5	3.175	3.3578(19)	3.462(3)
M1-C6	3.111(2)	3.3927(17)	3.535(3)
M2-C31	2.806(2)^d^	3.4063(18)	3.623(2)
M2-C32	2.724(2)^e^	3.402(2)	3.615(2)
M2-C33	2.722(2)^f^	3.331(2)	3.538(2)
M2-C34	2.817(2)^g^	3.264(2)	3.486(3)
M2-C35	2.913(2)^h^	3.267(2)	3.454(3)
M2-C36	2.930(2)^i^	3.3513(18)	3.509(2)
M2-N3	2.3164(18)		
N1–Al1–N2	111.73(8)	109.07(7)	109.64(9)
N3–Al2–N4		110.07(7)	108.50(9)

a M = Na.

b M = Rb.

c M = Cs.

d Na1–C31.

e Na1–C32.

f Na1–C33.

g Na1–C34.

h Na1–C35.

i Na1–C36.

Although we have no specific rationale for this outcome, we have previously noted the tendency of seven-membered chelated magnesium derivatives of the {SiN^Dipp^} dianion to undergo ring opening and the adoption of similar bimetallic bridged structures.^41,42^ These observations, in conjunction with the notable specificity of the formation of **14**, lead us to suggest that the reaction stoichiometry presented in eq 1 for the synthesis of **14** is realistic and justifiable.
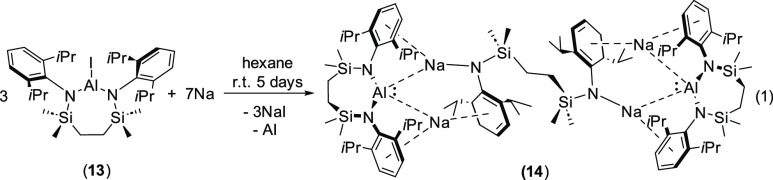
1

**Scheme 1:**
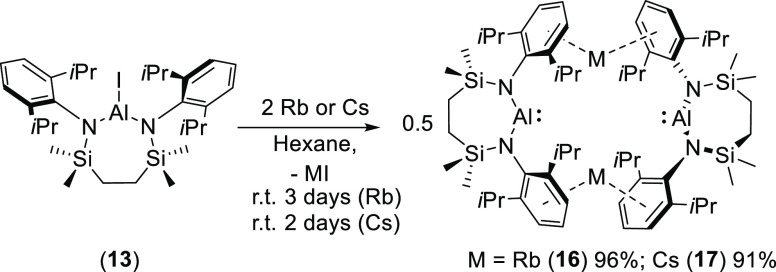
Synthesis of Compounds **16** and **17**

**Figure 3:**
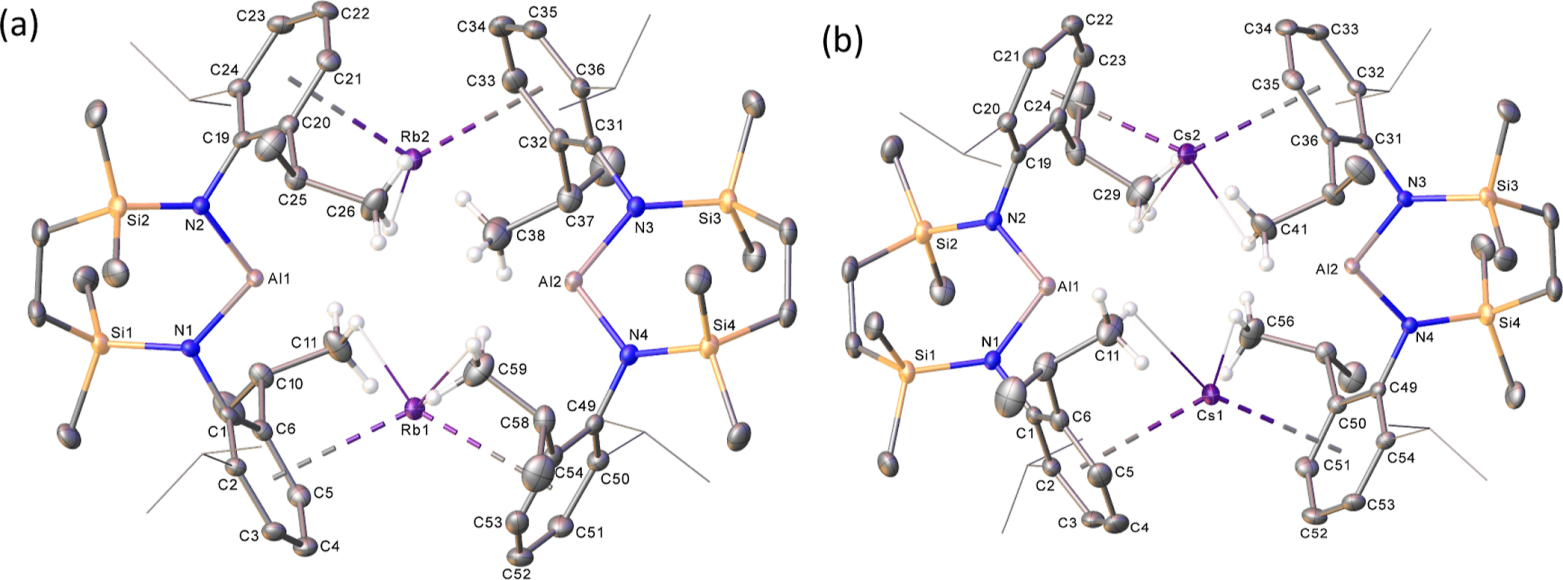
Displacement ellipsoid (30% probability) plots of the structures of (a) compound **16** and (b) compound **17**. For clarity, the solvent has been omitted, and with the exception of methyl groups that display close C–H···M contacts, hydrogen atoms are not shown. Most Dipp *iso*-propyl substituents are presented as wireframes, also for visual ease.

In contrast to these observations, reduction of **13** with Rb and Cs metals in hexane proceeded to completion at 30 °C in 3 and 2 days, respectively, to provide bright-yellow solutions of the heavier alkali metal alumanyls, compounds **16** and **17** (Scheme 1). Filtration and removal of volatiles at this point provided effectively pure samples of both compounds as yellow crystalline solids. Both **16** and **17** presented ^1^H and ^13^C NMR spectra that were strongly reminiscent of those of the analogous potassium derivative (**12**), indicative of a symmetrical *N*,*N*-chelated disposition of the dianilide ligand about aluminum. This inference was shown to be correct through the isolation of single crystals of both compounds, which were obtained by slow evaporation of hexane solutions at room temperature. The results of the subsequent X-ray analyses (Figure 3a,b) confirmed that, like **12**, both compounds crystallize as contact ion pairs in which the two, formally anionic, [(SiN^Dipp^)Al]^−^ subunits are solely connected by 2-fold polyhapto-Rb/Cs···π-arene bridging interactions. The mean M···centroid distances of 3.099 (**16**: M = Rb) and 3.247 (**17** M = Cs) Å are comparable to those displayed by the previously reported rubidium (**5** and **9**) and cesium (**6** and **10**) alumanyl derivatives and reflect the differing radii of the respective M^+^ cations.^43^ As was highlighted by Coles and Mulvey,^37^ the enhanced conformational freedom accorded to the dimeric structure by increasing alkali metal cation radius is also reflected in the dihedral angles subtended by the N1-A1-N2 and N3–Al2–N4 least-squares planes, which increase in the order **12** (M = K: 48.11°)^29^ < **16** (M = Rb: 62.33°) < **17** (M = Cs: 64.52°). The two latter values are reminiscent of those reported for the comparable metrics in compounds **5** and **6** such that **16** and **17** may also be described as presenting similarly “twisted” dimeric structures.

### Activation of Arene Solvents

**Scheme 2:**
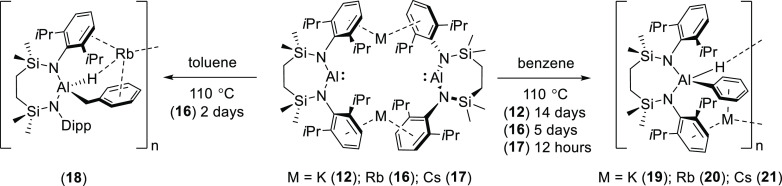
Synthesis of Compounds **18–21**

**Figure 4:**
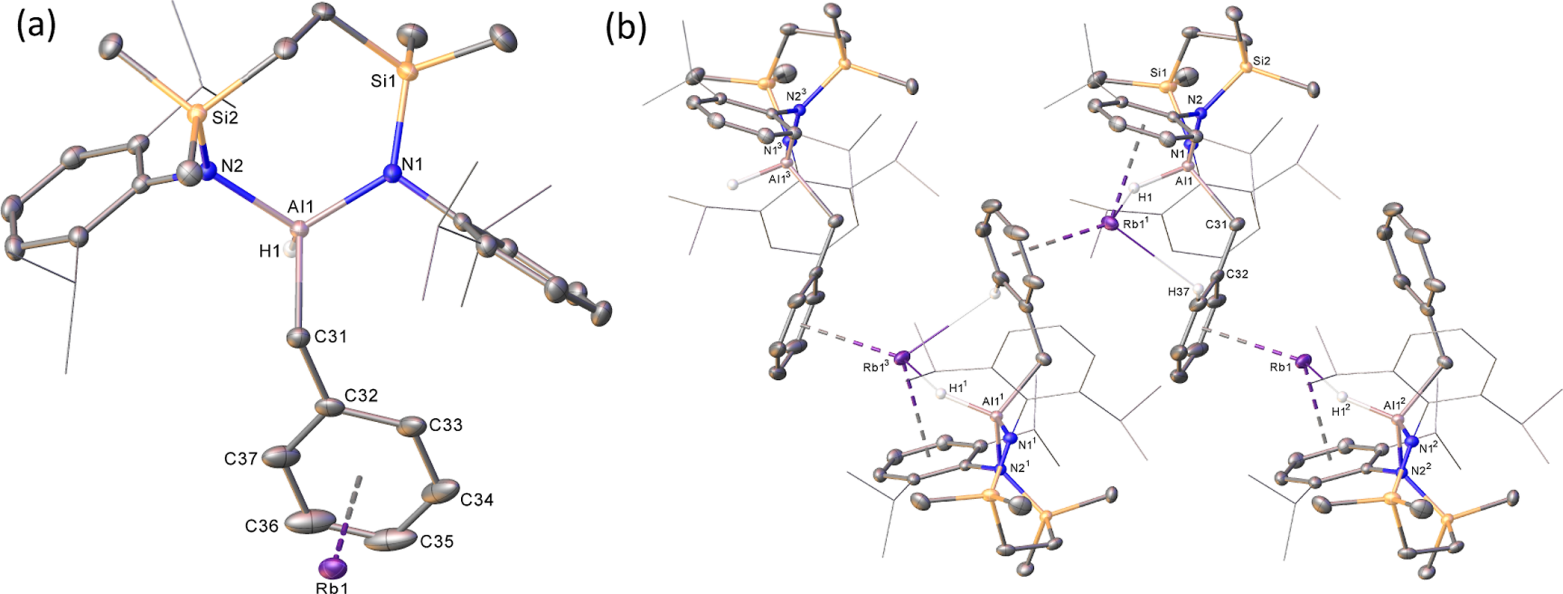
Displacement ellipsoid plots (30% probability) of (a) the monomeric unit and (b) the polymeric structure of compound **18**. Symmetry operations: ^1^3/2 – *x*, – 1/2 + *y*, 1/2 – *z*; ^2^ 3/2 – *x*, 1/2 + *y*, and 1/2 – *z*. For clarity, in both plots, hydrogen atoms have been removed with the exception of those which are aluminum-bound. Dipp *iso*-propyl substituents are represented as wireframes in (a), and disorder has been omitted throughout. In (b), entire Dipp substituents are denoted in wireframe mode, also for visual ease.

We have previously reported that compound **12** initiates the formal oxidative addition of a toluene-methyl C–H bond when heated at 110 °C for 24 h.^17^ In an attempt to extend this chemistry to the heavier alkali metal congeners, samples of the rubidium and cesium alumanyls, compounds **16** and **17**, were similarly heated in *d*_8_-toluene at 110 °C. Monitoring of the reactions over the course of 2 days by NMR spectroscopy provided evidence for the consumption of both alumanyl reagents and the generation of a complex mix of products. Although the cesium-containing reaction mixture proved intractable toward further purification, storage of the rubidium-derived solution at low temperature provided a small number of colorless single crystals of compound **18**, which could be separated mechanically from the mixture of otherwise unidentifiable products formed. X-ray diffraction analysis of **18** allowed its identification as the rubidium (hydrido)(benzyl)aluminate, [{(SiN^Dipp^)Al(H)(CH_2_Ph)}Rb], resulting from reductive activation of a toluene C(sp^3^)–H bond (Scheme 2). The results of this analysis, depicted in Figure 4, with selected bond lengths and angles summarized in Table 2, demonstrated that **18** is isostructural to its previously reported potassium analogue,^17^ crystallizing as an array of 4-coordinate diamido aluminate anions, which propagate along the crystallographic *b* axis as a 1-dimensional polymer via a series of Al–H–Rb, Rb-η^6^-Dipp, and Rb-η^6^-benzyl interactions.

**Table 2:** Selected Bond Lengths (Å) and Angles (deg) of Compounds **18–21**

	18^a^	19^b^	20^a^	21^c^
Al1–N1	1.8652(16)	1.877(2)	1.878(2)	1.873(3)
Al1–N2	1.8749(15)	1.867(4)	1.877(2)	1.889(3)
Al2–N3			1.885(2)	1.873(3)
Al2–N4			1.873(2)	1.881(3)
Al1–C31	2.032(2)	2.000(3)	2.010(3)	2.016(3)
Al2–C67			2.012(3)	2.012(3)
M1-C21	3.323(2)^d^	3.214(3)	3.298(3)	3.498(4)^g^
M1-C22	3.303(2)^e^	3.142(3)	3.230(3)	3.425(4)^h^
M1-C23	3.236(2)^f^	3.481(4)	3.420(3)	3.478(4)^i^
M1-C34	3.312(2)	3.380(4)	3.534(4)	3.628(4)
M1-C35	3.375(3)	3.128(4)	3.258(3)	3.415(4)
M1-C36	3.351(3)	3.393(3)	3.382(3)	3.456(3)
N1–Al1–N2	112.26(7)	111.8(2)	112.82(10)	112.99(12)
N1–Al1–C31	110.61(8)	114.02(11)	108.46(10)	108.56(13)
N2–Al1–C31	110.19(8)	106.7(2)	108.46(10)	113.27(13)

a M = Rb.

b M = K.

c M = Cs.

d Rb1–C21.^1^

e Rb1–C22.^1^

f Rb1–C23.^1^

g Cs1–C3.

h Cs1–C4.

i Cs1–C5.

In related observations, Aldridge and co-workers have previously reported that heating compound **1** in toluene for 2 days at 80 °C provides two products, which cocrystallize in a 3:1 ratio and result from *meta*-aryl and benzylic C–H bond activation, respectively.^6^ In a subsequent report, Yamashita and co-workers observed that toluene solutions of a potassium dialkylalumanyl variant provided the corresponding potassium (hydrido)(*m*-tolyl)aluminate as the sole product when simply allowed to stand at room temperature.^15^ These observations were ascribed to the operation of cooperative S_N_Ar processes, in which attack of the aluminum nucleophiles is facilitated by simultaneous complexation of the arene π-system by potassium. In this manner, the observed high levels of *meta*-C–H discrimination could be rationalized on the basis of the preferred charge distribution associated with the resultant Meisenheimer-type intermediates. While the behavior of compounds **16** and **17** toward toluene prompts us to suggest that similar mechanisms may be operable, further meaningful comment is precluded by the apparently reduced levels of discrimination provided by the {SiN^Dipp^}-derived systems.

The potassium diamido- and dialkylalumanyl species described by Aldridge and Yamashita have also been reported to effect similar C(sp^2^)–H alumination of benzene under relatively mild conditions (57 °C and room temperature, respectively). In contrast, the sole representative example of analogous reactivity among Coles and co-workers’ NON-supported systems (**2–6**) was provided by the cesium species (**7**), which also required more forcing conditions (5 days at 80 °C). With these observations in mind, therefore, we assessed the thermal stability of the respective potassium (**12**), rubidium (**16**), and cesium (**17**) alumanyl dimers in benzene solution. Although all three solutions required heating to 110 °C for the reactions to proceed at an appreciable rate, a gradual decolorization was observed in each case. Monitoring by ^1^H NMR spectroscopy indicated that the reactions were complete after 14 days (**12**), 5 days (**16**), and 12 h (**17**), while the disappearance of the alumanyl starting materials was accompanied by the simultaneous deposition of colorless crystals of the alkali metal (hydrido)(phenyl)aluminates, compounds **19** (75%), **20** (67%), and **21** (60%), resulting from the potassium-, rubidium-, and cesium-based reactions, respectively (Scheme 2). Although all three compounds proved to be insufficiently soluble in benzene for further mechanistic/kinetic analysis of their formation, their characterization by NMR spectroscopy was readily achieved by redissolution in THF-*d*_8_. While the resultant data provided convincing corroborative evidence for the formation of the mooted phenylaluminum products of benzene C–H activation, single-crystal X-ray diffraction analysis again delivered a definitive demonstration of the constitution of all three compounds.

**Figure 5:**
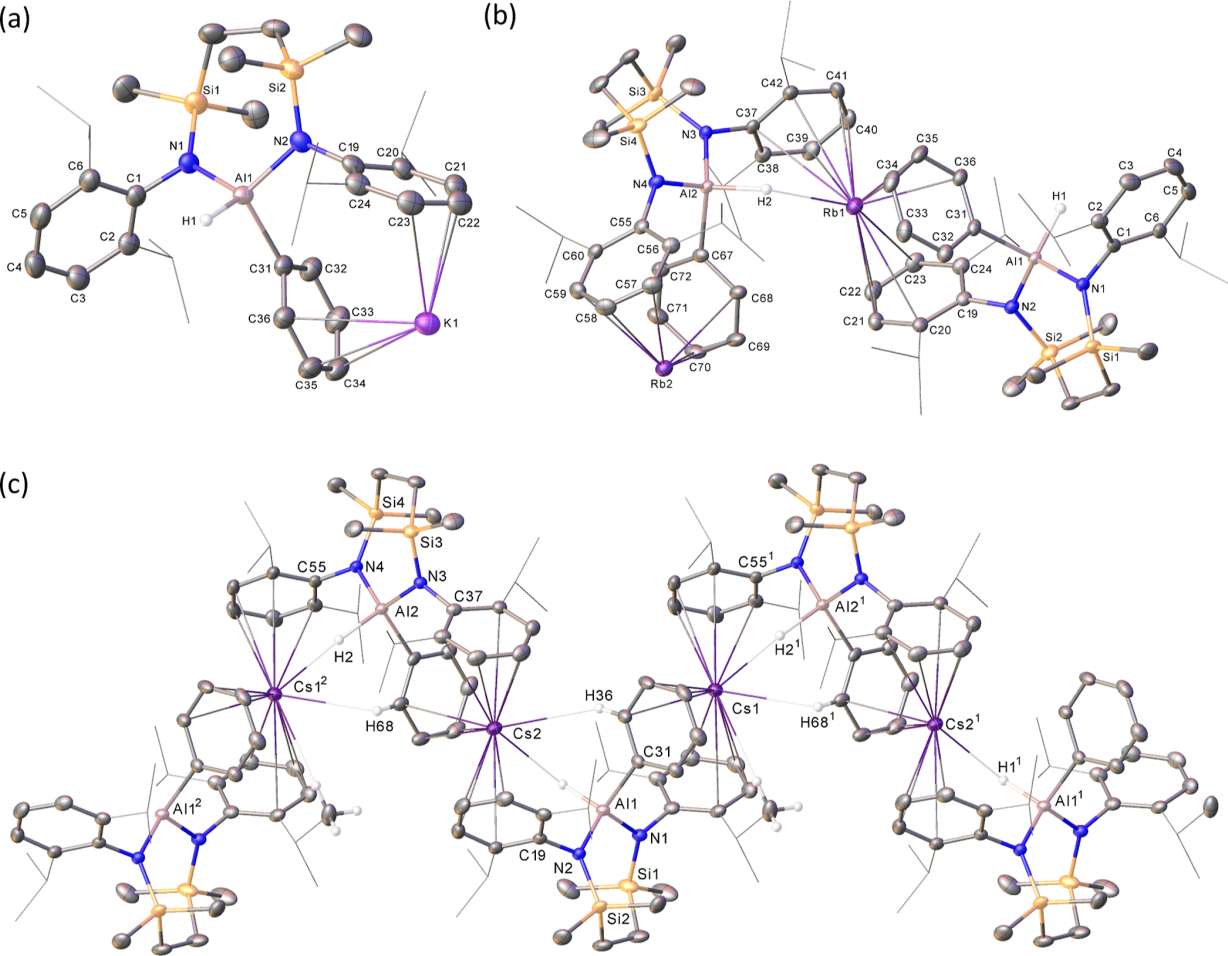
Displacement ellipsoid plots (30% probability) of the asymmetric units of (a) **19** and (b) **20**. (c) Polymeric structure of compound **21**. Symmetry operations (**21**): ^1^*x*, 1 + *y*, *z*;^2^*x*, – 1 + *y*, *z*. For clarity, hydrogen atoms have been removed with the exception of those which are aluminum-bound or involved in C–H···M interactions. Most Dipp *iso*-propyl substituents are presented as wireframes, and disorder has also been omitted from **19** for visual ease.

Selected bond length and angle data for all three compounds are presented in Table 2, and the asymmetric units of the potassium and rubidium (hydrido)(phenyl)aluminates, **19** and **20**, are illustrated in Figure 5a,b, respectively. Although only the triclinic structures of **20** and **21** are crystallographically isomorphous, any variation in the unit cell dimensions and volume is traceable to the adjustment in the ionic radii of the K^+^, Rb^+^, and Cs^+^ cations,^43^ an observation that is also reflected in the various M–C bonds observed across the three structures (Table 2). Like **18**, all three compounds crystallize as one-dimensional polymers, with propagation provided by encapsulation of the M^+^ ions, which span between adjacent pairs of aluminate anions via an alternating sequence of η^6^-M^+^-phenyl/η^6^-M^+^-Dipp-N and Al–H-M^+^/η^6^-M^+^-Dipp-N interactions. This is illustrated for cesium derivative **21** in Figure 5c. Despite the changing identity of the group 1 counter cations, the aluminate anions of all three structures show only minor variations across the various Al–N and Al–C bonds and within the chelated {SiN^Dipp^} ligands.

### DFT Calculations

Although Roesky’s monometallic β-diketiminate derivative, [HC{C(Me)NDipp)_2_Al],^44^ has been widely employed as a highly reducing and hydrocarbon-soluble source of aluminum(I),^45–64^ unless promoted by palladium catalysis,^65^ it has been reported as inert toward arene solvents.^66^ This behavior contrasts significantly with many of the alkali metal alumanyl derivatives summarized in Figure 1. It is notable, however, that benzene C–H activation, whether single^1,6,15,37,38^ or 2-fold,^22,36^ has been limited to alumanyl derivatives comprising a heavier (K–Cs) alkali metal cocation. In accord with earlier calculations on potassium alumanyls reported by Aldridge^6^ and Yamashita,^15^ Mulvey, Coles, McMullin, and co-workers attributed benzene activation by [{(NON)AlCs]_2_ (**6**) to the operation of a synergistic AMM effect, in which the softer, heavier Cs^+^ cation is best disposed to engage the benzene π system toward nucleophilic alumanyl attack.^37,67^ Although, among the series of [{(NON)AlM]_2_ [M = Li – Cs] derivatives, the acquisition of experimental evidence was unique to **6**, DFT calculations ascribed only a marginally diminished kinetic aptitude (*ca*. 5 kcal mol^–1^) for benzene C–H activation arising from its Rb analogue (**5**) and irrespective of whether a mono- or dimeric pathway was computed (vide infra).

**Figure 6:**
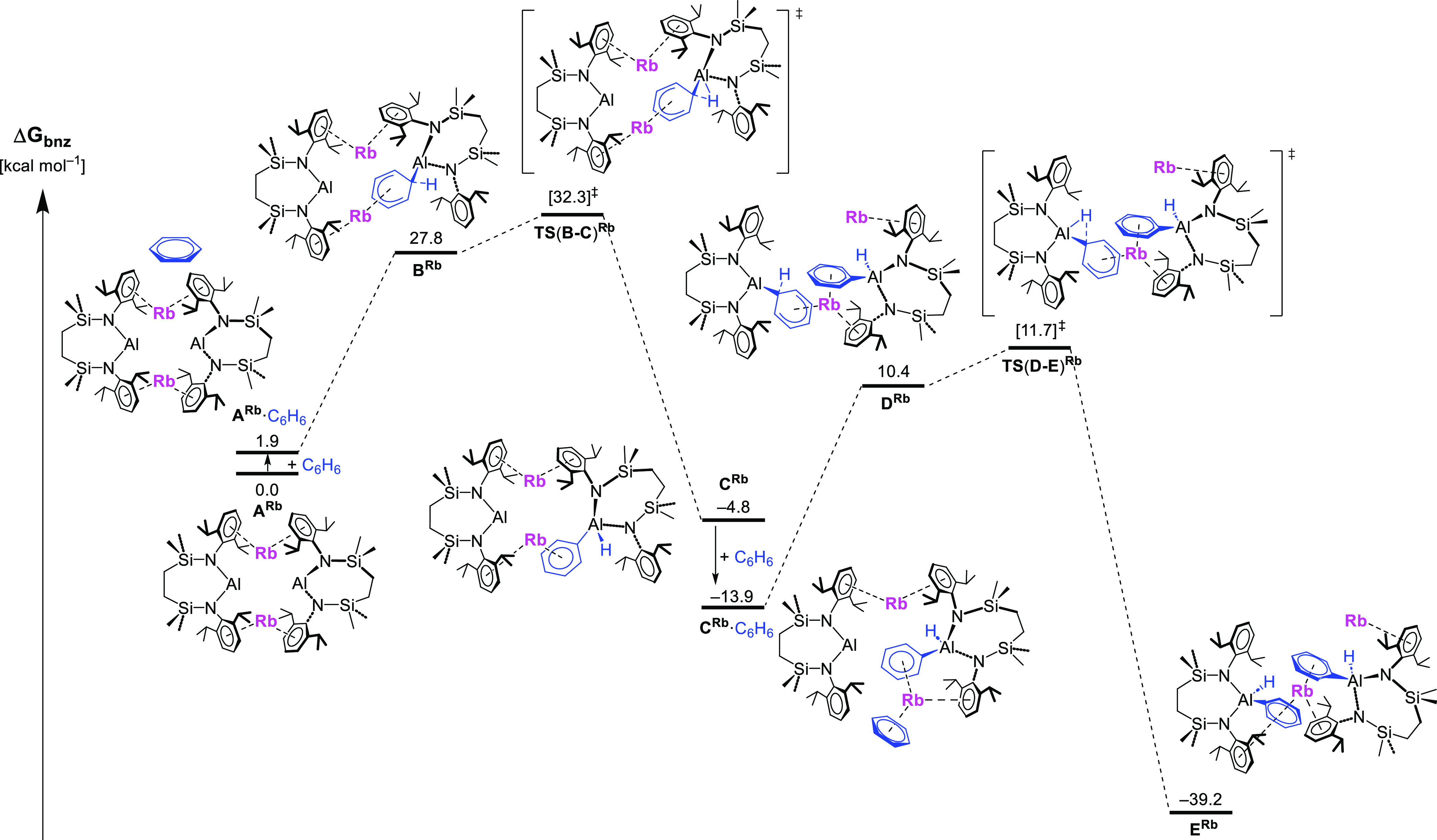
Computed free energy profile (BP86-D3BJ(PCM = C_6_H_6_)/BS2//BP86/BS1 level, energies quoted in kcal mol^–1^) for the formation of the dimeric rubidium (hydrido)(phenyl)aluminate **E**^**Rb**^.

**Figure 7:**
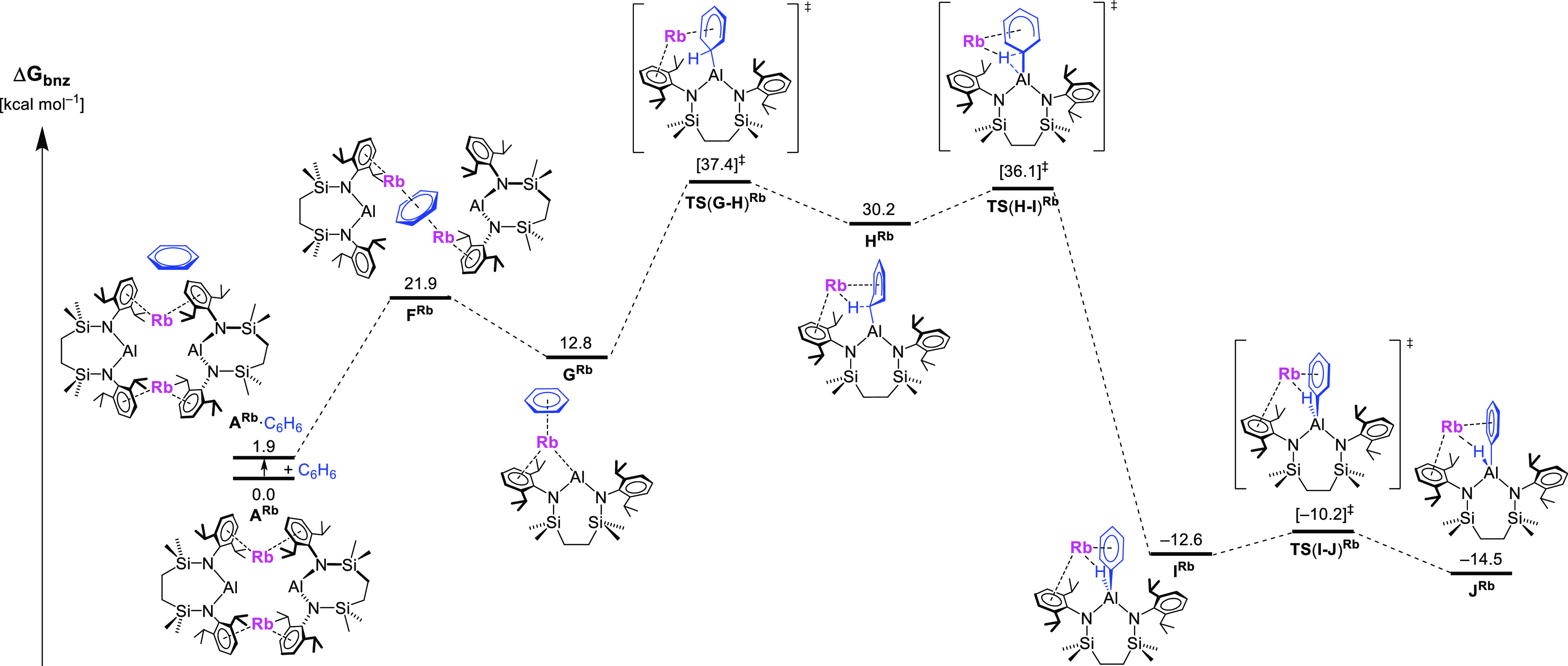
Computed free energy profile (BP86-D3BJ(PCM = C_6_H_6_)/BS2//BP86/BS1 level, energies quoted in kcal mol^–1^) for the formation of the monomeric rubidium (hydrido)(phenyl)aluminate, **J**^**Rb**^.

The isolation of compounds **19–21**, therefore, and the variable facility of their formation provide a coherent series with which to further assess the consequences of varying alkali metal identity on the arene reactivity of dimeric alumanyl derivatives. Accordingly, and in a comparable manner to the recent computational analysis of the reaction of benzene with [(NON)AlM]_2_ (M = Rb and Cs),^37^ two pathways (described herein as “dimeric” and “monomeric”) were explored for benzene C–H activation by the series of [{SiN^Dipp^}AlM]_2_ (where M = Na, K, Rb, and Cs) (see Supporting Information; Figures S25 and S26). Although similar profiles were calculated and the subsequent discussion relates to all four alkali metal species, for illustrative purposes, Figures 6 and 7 are restricted to the data arising from the respective dimeric and monomeric pathways for the rubidium-derived reaction only.

The dimeric pathway achieves benzene activation without any requirement for the initially dimeric alumanyl to dissociate into monomeric units. The association of one benzene molecule to the dialumanyl complex (**A**) forms species **B**, which then undergoes the first C–H activation via a Meisenheimer-like transition state, **TS(B–C)**. Notably, and reminiscent of several recent reports in which a benzene derivative is activated through its polyhapto engagement with a heavier *s*-*block* or Yb(II) cation,^1,6,15,36,38,68–72^ the assembly of **TS(B–C)** is facilitated by similar π engagement and resultant rear-side nucleophilic attack by one of the low oxidation state aluminum centers. Although this process disrupts the previously symmetrical structure of the alumanyl dimer (**A**), the resultant (hydrido)(phenyl)aluminate does not completely dissociate from the remaining alumanyl unit. Rather, contact between both [(SiN^Dipp^)Al] chelate structures is maintained in **C** through a persistent Dipp-M···Dipp bridging interaction. A second benzene molecule is, however, able to interact with the now more exposed group 1 metal, which remains coordinated via the phenyl ligand of the newly formed phenylaluminate in **C**·C_6_H_6_. In a manner similar to the initial C–H activation process, attack of the second alumanyl proceeds via the formation of **D** and **TS(D-E)**, the magnitude of which is closely comparable to **TS(B-C)** (Table 3). The thermodynamic viability of the overall transformation is then ensured by the stability of the ultimate (dihydrido)(diphenyl)dialuminate complex (**E**).

**Table 3:** Gibbs Free Energy Barriers (in kcal mol^–1^) for the C–H Activation of Benzene in the Alumanyl Na–Cs Series (BP86-D3BJ(PCM = C_6_H_6_)/BS2//BP86/BS1)

	M =	Na	K	Rb	Cs
dimeric-1st C–H activation	**TS(B–C)**^**M**^	39.8	34.1	32.3	28.8
dimeric-2nd C–H activation	**TS(D-E)**^**M**^	41.8	27.9	25.6	24.5
monomeric C_6_H_6_Al-association	**TS(G-H)**^**M**^	45.0	38.7	37.4	35.3
monomeric C–H activation	**TS(H-I)**^**M**^	41.1	36.6	36.1	34.5

The alternative monomeric pathway first requires the cleavage of the dimer (**A**), which proceeds via species **F** through the insertion of a single benzene molecule into the Al_2_M_2_ diamond. This process increases the separation between the Al centers to over 10 Å and ultimately develops into species **G**, a monomeric alumanyl in which benzene displays an η^6^-interaction with the group 1 metal cation. From **G**, Al-induced C–H activation occurs in a somewhat analogous fashion to that outlined for the previously described dimeric pathway. Attack of the Al(I) nucleophile at benzene provides an initial intermediate **H** via **TS(G-H)**, whereupon cleavage of the C–H bond via **TS(H-I)** yields a monomeric (hydrido)(phenyl)aluminate, **I**. The final step of this pathway involves a small rotation of the phenyl group at aluminum through **TS(I-J)** to give the more stable conformer **J**.^73^

The free energies arising from both computed pathways (Table 3) show the anticipated trend, where the ultimate products, **E**^**K**^/**J**^**K**^**,E**^**Rb**^/**J**^**Rb**^ and **E**^**Cs**^/**J**^**Cs**^, represent monomeric variants of the polymeric (hydrido)(phenyl)aluminate species, **19**, **20**, and **21**, respectively. Although experimentally unavailable for assessment, the highest activation barriers presented by both pathways are associated with the sodium-derived species and then decrease incrementally as group 1 descends. In every case, however, the sequential dimeric pathway is favored mechanistically, providing barrier heights that are congruent with the necessary experimental conditions and predicting that the first C–H activation step via **TS(B-C)** is rate-determining for all three reactions available for experimental assessment.

## Conclusions

Reduction of [{SiN^Dipp^}AlI] with the alkali metals K, Rb, and Cs provides similarly dimeric group 1 alumanyl derivatives. Although the analogous reaction with sodium also results in the isolation of an alumanyl species, a consistent level of over-reduction leads to the incorporation of a formal equivalent of [{SiN^Dipp^}Na_2_] into the resultant structure. The dimeric (K, Rb, and Cs) species all react with arene solvents at elevated temperatures to yield (hydrido)organoaluminate species, while computational assessment of the benzene C–H activation indicates that the rate-determining attack of the Al(I) nucleophile is facilitated by π-engagement of the arene with the electrophilic cation, which becomes increasingly favorable as group 1 is descended.

## Experimental Section

Unless stated otherwise, all of the experiments were conducted using standard Schlenk line and glovebox techniques under an inert atmosphere of argon. NMR spectra were recorded with a Bruker AVANCE III spectrometer (^1^H at 400 MHz; ^13^C at 101 MHz). The spectra are referenced relative to residual protio solvent resonances. Elemental analyses were performed at Elemental Microanalysis Ltd., Okehampton, Devon, UK. Solvents were dried by passage through a commercially available solvent purification system and stored under argon in ampoules over 4 Å molecular sieves. Benzene-*d*_6_ and THF-*d*_8_ were purchased from Sigma-Aldrich and dried over a potassium mirror before distilling and storage over molecular sieves. [{SiN^Dipp^}AlI] (**13**) and [{SiN^Dipp^}AlK]_2_ (**12**) were prepared according to the reported procedures.^29^ All other chemicals were purchased from Merck and used without further purification.

### Synthesis of [{SiN^Dipp^}AlNa({SiN^Dipp^}Na_2_)NaAl{SiN^Dipp^}] (14)

A Schlenk tube was charged with [{SiN^Dipp^}AlI] (**13**, 0.648 g, 1.0 mmol) and excess Na/NaCl (5 wt.%, 1.5 g),^41^ before the addition of hexane (25 mL) via a cannula. The reaction mixture was then stirred for 3 days at room temperature, affording a pale-yellow solution with a gray suspension. The crude mixture was then filtered and concentrated (to about 5 mL). Storage of the concentrated solution at −10 °C for 3 days afforded **14** as a colorless, crystalline powder. Yield 0.21 g, 77%. A single crystal suitable for X-ray diffraction analysis was then selected from the batch of recrystallized solids. The reaction was then repeated on a smaller scale for monitoring by NMR spectroscopy. [{SiN^Dipp^}AlI] (**13**, 16 mg, 0.025 mmol) was dissolved in C_6_D_6_ in a J. Young NMR tube before addition of excess Na/NaCl (5 wt.%, 30 mg) to the colorless solution. The reaction mixture was then kept at 30 °C and continually shaken for 5 days. A ^1^H NMR spectrum obtained from the small-scale reaction was virtually identical to that of the isolated sample, indicating the exclusive formation of **14** under the applied reaction conditions. Despite repeated attempts, an accurate microanalysis could not be obtained for this compound. ^1^H NMR (400 MHz, 298 K, Benzene-*d*_6_): δ 7.05 (d_app_, 4H, m-C_6_*H*_3_), 7.00–6.84 (m, 13H, C_6_*H*_3_), 6.72 (t_app_, 1H, *p*-C_6_*H*_3_), 4.00 (sept, *J* = 6.8 Hz, 4H,C*H*Me_2_), 3.87–3.68 (m, 8H, C*H*Me_2_), 1.33–1.22 (m, 45H, CH*Me*_2_), 1.19* (d, *J* = 6.8 Hz, CH*Me*_2_), 1.18* (d, *J* = 6.8 Hz, 6H, CH*Me*_2_), * overlapping doublets, 1.15 (s, 4H, SiC*H*_2_), 1.13 (s, 8H, SiC*H*_2_), 1.10–1.04 (m, 15H, CH*Me*_2_), 0.26–0.12 (m, 36H, Si*Me*_2_). ^13^C{^1^H} NMR (101 MHz, 298 K, Benzene-*d*_6_): δ 148.2 (*i*-*C*_6_H_3_), 148.1 (*i*-*C*_6_H_3_), 145.7 (*o*-*C*_6_H_3_), 145.4 (*o*-*C*_6_H_3_), 123.9 (*m*-*C*_6_H_3_), 123.5 (*m*-*C*_6_H_3_), 123.4 (*p*-*C*_6_H_3_), 122.9 (*p*-*C*_6_H_3_), 28.7 (*C*HMe_2_), 28.4 (*C*HMe_2_), 27.9 (*C*HMe_2_), 27.5 (*C*HMe_2_), 25.6 (CH*Me*_2_), 25.6 (CH*Me*_2_), 25.1 (CH*Me*_2_), 24.9 (CH*Me*_2_), 24.7 (CH*Me*_2_), 24.4 (CH*Me*_2_), 24.2 (CH*Me*_2_), 24.1 (CH*Me*_2_), 14.6 (Si*C*H_2_), 14.3 (Si*C*H_2_), 14.2 (Si*C*H_2_), 13.4 (Si*C*H_2_), 1.5 (Si*Me*_2_), 1.2 (Si*Me*_2_), 0.0 (Si*Me*_2_), −0.2 (Si*Me*_2_).

### Synthesis of [({SiN^Dipp^}Al)Rb]_2_ (16)

A Schlenk tube was charged with [{SiN^Dipp^}AlI] (**13**, 0.324 g, 0.500 mmol) and Rb metal (0.140 g, 1.655 mmol) before hexane (30 mL) was added via a cannula. The reaction mixture was then stirred at 30 °C for 3 days, giving a bright-yellow solution with a gray suspension. The mixture was filtered through a cannula filter, and the bright-yellow filtrate was collected and put under reduced pressure to remove all volatiles, affording [({SiN^Dipp^}Al)Rb]_2_ (**16**) as a bright-yellow crystalline powder. Yield 0.290 g, 96%. Anal. Calcd for C_60_H_100_Al_2_Rb_2_N_4_Si_4_ (**16**, 1212.48): C, 59.33; H, 8.30; N, 4.61%. Found: C, 59.42; H, 8.46, N, 4.48%. A single crystal suitable for X-ray diffraction analysis was obtained by slow evaporation of a hexane solution of **16** at room temperature. ^1^H NMR (500 MHz, 298 K, Benzene-*d*_6_): δ 6.89 (d, *J* = 7.6 Hz, 4H, *m*-C_6_*H*_3_), 6.75 (t, *J* = 7.6 Hz, 2H, *p*-C_6_*H*_3_), 4.04 (sept, *J* = 6.9 Hz, 4H, C*H*Me_2_), 1.30 (d, *J* = 6.9 Hz, 12H, CH*Me*_2_), 1.13 (s, 4H, SiC*H*_2_), 1.08 (d, *J* = 6.9 Hz, 12H, CH*Me*_2_), 0.22 (s, 12H, Si*Me*_2_). ^13^C{^1^H} NMR (126 MHz, 298 K, Benzene-*d*_6_): δ: 152.1 (*i*-*C*_6_H_3_), 149.1 (*o*-*C*_6_H_3_), 123.0 (*m*-*C*_6_H_3_), 122.1 (*p*-*C*_6_H_3_), 27.9 (*C*HMe_2_), 25.0 (CH*Me*_2_), 24.0 (CH*Me*_2_), 14.4 (Si*C*H_2_), 1.7 (Si*Me*_2_). *Hexane impurity observed within the sample.

### Synthesis of [({SiN^Dipp^}Al)Cs]_2_ (17)

[{SiN^Dipp^}AlI] (**13**, 0.324 g, 0.500 mmol) and Cs metal (0.220 g, 1.655 mmol) were charged into a Schlenk tube before hexane (30 mL) was added via cannula. The reaction mixture was then stirred at 30 °C for 2 h followed by further stirring at room temperature for 2 days, providing a bright-yellow solution with a gray suspension. The mixture was filtered through a cannula filter, the yellow filtrate was collected and put under reduced pressure to remove all volatiles to provide [({SiN^Dipp^}Al)Cs]_2_ (**17**) as a bright-yellow crystalline powder. Yield 0.274 g, 86%. A single crystal suitable for X-ray diffraction analysis was obtained by slow evaporation of a hexane solution of **17** at room temperature. Anal. Calcd for C_60_H_100_Al_2_Cs_2_N_4_Si_4_ (**17**, 1308.48): C, 55.03; H, 7.70; N, 4.28%. Found: C, 54.86; H, 7.88, N, 4.10%. ^1^H NMR (500 MHz, 298 K, Benzene-*d*_6_): δ 6.87 (d, *J* = 7.5 Hz, 4H, *m*-C_6_H_3_), 6.72 (t, *J* = 7.5 Hz, 2H, p-C_6_*H*_3_), 4.12 (sept, *J* = 7.0 Hz, 4H, C*H*Me_2_), 1.30 (d, *J* = 7.0 Hz, 12H, CH*Me*_2_), 1.13^†^ (s, 4H, SiC*H*_2_), 1.13^†^ (d, *J* = 7.0 Hz, 12H, CH*Me*_2_) ^†^overlapping peaks, 0.21 (s, 12H, Si*Me*_2_). ^13^C{^1^H} NMR (126 MHz, 298 K, Benzene-*d*_6_): δ: 153.1 (*i*-*C*_6_H_3_), 149.1 (*o*-*C*_6_H_3_), 123.4 (*m*-*C*_6_H_3_) 122.0 (*p*-*C*_6_H_3_), 28.0 (*C*HMe_2_), 24.9 (CH*Me*_2_), 24.0 (CH*Me*_2_), 14.4 (Si*C*H_2_), 1.6 (Si*Me*_2_). *Hexane impurity observed within the sample.

### Reaction of [({SiN^Dipp^}Al)Rb]_2_ (16) with C_7_H_8_; Identification of [{(SiN^Dipp^)Al(H)(CH_2_Ph)}Rb] (18)

Inside a J. Young tube, [({SiN^Dipp^}Al)Rb]_2_ (**16**, 30 mg, 0.025 mmol) was dissolved with 0.4 mL of toluene to afford a bright yellow solution. The reaction mixture was then kept at 110 °C for 2 days, giving a colorless solution with a very small amount of gray precipitate. The tube was then taken into a glovebox, and the solution was filtered before layering hexane (0.4 mL) onto the colorless filtrate in a vial. Storage of the vial at ambient temperature provided a mixture of crystals and some white powder, of which the crystals were identified as **(18)** by X-ray diffraction analysis. Washing the crystals with hexane (0.2 mL × 2) and removing amorphous solids, followed by removal of all volatiles in vacuo provided a sample which was dissolved in *d*_8_-THF and analyzed by ^1^H NMR spectroscopy. Diagnostic septet resonances at *d*_H_ = 4.16 and 4.05 ppm were tentatively assigned to **18** among a mixture of other unidentifiable products. Multiple attempts with freshly dried and distilled solvents provided comparable results with varying ratios of the integration of the septet signals, and isolation of each crystal could only be achieved by mechanical separation.

### Synthesis of [{(SiN^Dipp^)Al(H)(C_6_H_5_)}K] (19)

[({SiN^Dipp^}Al)K]_2_ (**12**, 28 mg, 0.025 mmol) was dissolved in a J. Young tube with 0.4 mL of benzene to afford a bright yellow solution. The reaction mixture was then kept at 110 °C for 14 days, during which time a gradual decolorization of the mixture was observed. After 2 weeks, colorless crystals were observed to have formed within the pale-orange hazy solution. The supernatant was then carefully decanted, and the colorless crystals were collected and washed with hexane (0.4 mL × 2) before removal of all volatiles to afford **19** as a colorless crystalline powder. Yield 24 mg, 75%. A single crystal suitable for X-ray diffraction analysis was selected from the crystals thus obtained. Despite repeated attempts, an accurate microanalysis could not be obtained for this compound. ^1^H NMR (400 MHz, 298 K, THF-*d*_8_): δ 7.12–7.05 (m, 2H, *o*-C_6_*H*_5_), 6.83–6.77 (m, 2H, *p*-C_6_*H*_3_ on SiN^Dipp^), 6.68–6.62 (m, 3H, Ar*H*), 6.63–6.57 (m, 4H, *m*-C_6_*H*_3_ on SiN^Dipp^), 4.28 (sept, *J* = 6.9 Hz, 2H, C*H*Me_2_ on SiN^Dipp^), 3.94 (p, *J* = 6.9 Hz, 2H, C*H*Me_2_ on SiN^Dipp^), 1.17 (d, *J* = 6.9 Hz, 6H, CH*Me*_2_ on SiN^Dipp^), 1.14 (d, *J* = 6.9 Hz, 6H, CH*Me*_2_ on SiN^Dipp^), 1.07 (d, *J* = 6.9 Hz, 6H, CH*Me*_2_ on SiN^Dipp^), 1.03–0.93 (m, 4H, SiC*H*_2_), 0.42 (d, *J* = 6.9 Hz, 6H, CH*Me*_2_ on SiN^Dipp^), 0.02 (s br, 12H, Si*Me*_2_). ^1^H resonance correlated to Al–*H* was not observed. ^13^C{^1^H} NMR (101 MHz, 298 K, THF-*d*_8_): δ 151.4 (*i*-*C*_6_H_3_ on SiN^Dipp^), 148.5 (*o*-*C*_6_H_3_ on SiN^Dipp^), 148.0 (*o*-*C*_6_H_3_ on SiN^Dipp^), 140.0 (*o*-*C*_6_H_5_), 125.8 (*m*-*C*_6_H_5_), 124.6 (*p*-*C*_6_H_5_), 123.5 (*m*-*C*_6_H_3_ on SiN^Dipp^), 123.2 (*m*-*C*_6_H_3_ on SiN^Dipp^), 121.3 (*p*-*C*_6_H_3_ on SiN^Dipp^), 27.9 (*C*HMe_2_), 27.9 (*C*HMe_2_), 26.5 (CH*Me*_2_), 26.4 (CH*Me*_2_), 26.2 (CH*Me*_2_), 26.0 (CH*Me*_2_), 16.2 (Si*C*H_2_), 2.4 (Si*Me*_2_), 1.7 (Si*Me*_2_). ^13^C resonance correlated to Al–*C* (*i-C*_6_H_5_) was not observed.

### Synthesis of [{(SiN^Dipp^)Al(H)(C_6_H_5_)}Rb] (**20**)

[({SiN^Dipp^}Al)Rb]_2_ (**16**, 30 mg, 0.025 mmol) was dissolved with 0.4 mL of benzene in a J. Young tube to afford a bright yellow solution. The reaction mixture was then kept at 110 °C for 5 days, during which time a gradual decolorization of the mixture was observed. After 5 days, colorless crystals were observed to have formed within the pale-orange hazy solution. The supernatant was carefully decanted, and the colorless crystals were collected and washed with hexane (0.4 mL × 2) before removal of all volatiles to afford **20** as a colorless crystalline powder. Yield 23 mg, 67%. A single crystal suitable for X-ray diffraction analysis was selected from the crystals thus obtained. Despite repeated attempts, an accurate microanalysis could not be obtained for this compound. ^1^H NMR (400 MHz, 298 K, THF-*d*_8_): δ 7.28–7.20 (m, 2H, *o*-C_6_*H*_5_), 6.85–6.87 (m, 2H, *p*-C_6_*H*_3_ on SiN^Dipp^), 6.73–6.58 (m, 7H, Ar*H*), 4.30 (sept, *J* = 6.8 Hz, 2H, C*H*Me_2_), 3.93 (sept, *J* = 6.8 Hz, 2H, C*H*Me_2_), 1.25 (d, *J* = 6.8 Hz, 6H, CH*Me*_2_), 1.16 (d, *J* = 6.8 Hz, 6H, CH*Me*_2_), 1.08 (d, *J* = 6.8 Hz, 6H, CH*Me*_2_), 1.04–0.94 (m, 4H, SiC*H*_2_), 0.41 (d, *J* = 6.8 Hz, 6H, CH*Me*_2_), 0.04 (s, 6H, Si*M*e_2_), 0.03 (s, 6H, Si*Me*_2_). ^1^H resonance correlated to Al–*H* was not observed. ^13^C{^1^H} NMR (298 K, THF-*d*_8_): δ 151.4 (*i*-*C*_6_H_3_ on SiN^Dipp^), 148.7 (*o*-*C*_6_H_3_ on SiN^Dipp^), 148.2 (*o*-*C*_6_H_3_ on SiN^Dipp^), 139.9 (*o*-*C*_6_H_5_), 126.3 (*m*-*C*_6_H_5_), 125.1 (*p*-*C*_6_H_5_), 123.7 (*o*-*C*_6_H_3_ on SiN^Dipp^), 123.5 (*o*-*C*_6_H_3_ on SiN^Dipp^), 121.6 (*p*-*C*_6_H_3_ on SiN^Dipp^), 27.9 (*C*HMe_2_), 26.4 (CH*Me*_2_), 26.3 (CH*Me*_2_), 26.1(CH*Me*_2_), 26.0 (CH*Me*_2_), 16.1 (Si*C*H_2_), 2.4 (Si*Me*_2_), 1.6 (Si*Me*_2_). ^13^C resonance correlated to Al-*C* (*i-C*_6_H_5_) was not observed. Hexane impurity was observed within the sample.

### Synthesis of [{(SiN^Dipp^)Al(H)(C_6_H_5_)}Cs] (**21**)

[({SiN^Dipp^}Al)Cs]_2_ (**17**, 33 mg, 0.025 mmol) was dissolved in a J. Young tube with 0.4 mL of benzene to afford a bright yellow solution. The reaction mixture was then kept at 110 °C for 12 h, during which time the mixture was observed to decolorize with the formation of a colorless white precipitate. The tube was then taken into the glovebox, and 0.1 mL of toluene was added to the reaction mixture. The reaction mixture was then kept at 60 °C, affording single crystals suitable for X-ray diffraction analysis. The supernatant was carefully decanted, and the colorless crystals were collected and washed with hexane (0.4 mL × 2) before removal of all volatiles, to afford **21** as a colorless crystalline powder. Yield 21 mg, 60%. Despite repeated attempts, an accurate microanalysis could not be obtained for this compound. ^1^H NMR (400 MHz, 298 K, THF-*d*_8_): δ 7.32–7.30 (m, 2H, *o*-C_6_*H*_5_), 6.88–6.86 (m, 2H, *p*-C_6_*H*_3_ on SiN^Dipp^), 6.76–6.70 (m, 3H, *m*- and *p*-C_6_*H*_5_), 6.70–6.59 (m, 4H, *m*-C_6_*H*_3_ on SiN^Dipp^), 4.32 (sept, *J* = 6.8 Hz, 2H, C*H*Me_2_), 3.93 (sept, *J* = 6.8 Hz, 2H, C*H*Me_2_), 1.28 (d, *J* = 6.8 Hz, 6H, CH*Me*_2_), 1.17 (d, *J* = 6.9 Hz, 6H, CH*Me*_2_), 1.09 (d, *J* = 6.8 Hz, 6H, CH*Me*_2_), 1.05–0.94 (m, 4H, SiC*H*_2_), 0.41 (d, *J* = 6.8 Hz, 6H, CH*Me*_2_), 0.05 (s, 6H, Si*Me*_2_), 0.02 (s, 6H, Si*Me*_2_). ^1^H resonance correlated to Al–*H* was not observed. ^13^C{^1^H} NMR (101 MHz, 298 K, THF-*d*_8_): δ 151.6 (*i*-*C*_6_H_3_ on SiN^Dipp^), 148.8 (*o*-*C*_6_H_3_ on SiN^Dipp^), 148.4 (*o*-*C*_6_H_3_ on SiN^Dipp^), 140.1 (*o*-*C*_6_H_5_), 129.2 (*m*-*C*_6_H_5_), 126.4 (*p*-*C*_6_H_5_), 125.3 (*m*-*C*_6_H_3_ on SiN^Dipp^), 123.9 (*m*-*C*_6_H_3_ on SiN^Dipp^), 121.8 (*p*-*C*_6_H_3_ on SiN^Dipp^), 28.0 (*C*HMe_2_), 26.3 (CH*Me*_2_), 26.2 (CH*Me*_2_), 26.1 (CH*Me*_2_), *plausibly one CH*Me*_2_ peak merged with ^13^C resonance of *d*_8_-THF, 16.1 (Si*C*H_2_), 2.4 (Si*Me*_2_), 1.6 (Si*Me*_2_). ^13^C resonance correlated to Al–*C* (*i-C*_6_H_5_) was not observed. Hexane impurity was observed within the sample.

## Computational Methodology

DFT calculations were run with Gaussian 16 (C.01).^74^ The Na, Al, Si, K, Rb, and Cs centers were described with the Stuttgart RECPs and associated basis sets,^75^ and the 6-31G** basis set was used for all other atoms (BS1).^76^ A polarization function was also added to Al (ζ_d_ = 0.190), Si (ζ_d_ = 0.284), K (ζ_d_ = 1.000), Rb (ζ_d_ = 0.491), and Cs (ζ_d_ = 0.306). Initial BP86 optimizations were performed using the “grid = ultrafine” option,^77^ with all stationary points being fully characterized via analytical frequency calculations as minima or transition states (all positive eigenvalues or one imaginary eigenvalue, respectively). All energies were recomputed with a larger basis set featuring 6-311++G** basis sets on all atoms with the exceptions of Rb and Cs, which used def2-TVZP (BS2). Corrections for the effect of benzene (ε = 2.2706) solvent were run using the polarizable continuum model and BS1,^78^ using the keyword “scrf = benzene” within Gaussian. Single-point dispersion corrections to the BP86 results employed Grimme’s D3 parameter set with Becke-Johnson damping, as implemented in Gaussian.^79^

## Supporting Information Available

NMR spectra of all compounds and details of the X-ray and computational analyses, including the various computed free energy profiles (PDF)
Coordinates for all optimized computed structures and transition states (XYZ)

## Supplementary Material

om3c00323_si_001.pdf

om3c00323_si_002.xyz
