# Supplementary material for: Seven-Membered Cyclic Diamidoalumanyls of Heavier Alkali Metals: Structures and C–H Activation of Arenes
Source: Organometallics. 2023 Sep 9;42(19):2881–92. doi: 10.1021/acs.organomet.3c00323 (PMC10565898; doi:10.1021/acs.organomet.3c00323)
Supplement: Supplementary file 1 — om3c00323_si_001.pdf [file om3c00323_si_001.pdf]

Supplementary Information for:

Seven-membered Cyclic Diamidoalumanyls of the Heavier Alkali Metals: Structures and C–H activation of Arenes

Han-Ying Liu, Michael S. Hill,* Mary F. Mahon,* Claire L. McMullin* and Ryan J. Schwamm

Department of Chemistry, University of Bath, Claverton Down, Bath, BA2 7AY, UK

1 Experimental and Supplementary Information

1.1 General information

Except stated otherwise, all the experiments were conducted using standard Schlenk line and/or glovebox techniques under an inert atmosphere of argon. NMR spectra were recorded with a Bruker Avance III spectrometer (^{1}H at 400 MHz, ^{13}C at 101 MHz). The spectra are referenced relative to residual protio solvent resonances. Elemental analyses were performed at Elemental Microanalysis Ltd., Okehampton, Devon, UK. Solvents were dried by passage through a commercially available solvent purification system and stored under argon in ampoules over 4 Å molecular sieves. Benzene-d_6 and THF-d_8 was purchased from Sigma-Aldrich, dried over a potassium mirror before distilling and storage over molecular sieves. {SiN^{Dipp}}AlI (**13**)[1] and [{SiN^{Dipp}}AlK]$_2$ (**12**)[1] was prepared according to reported procedures. All other chemicals were purchased from Merck and used without further purification.

Figure S1: ^{1}H NMR (400MHz, 298 K, d_6-benzene) spectrum of **14.**

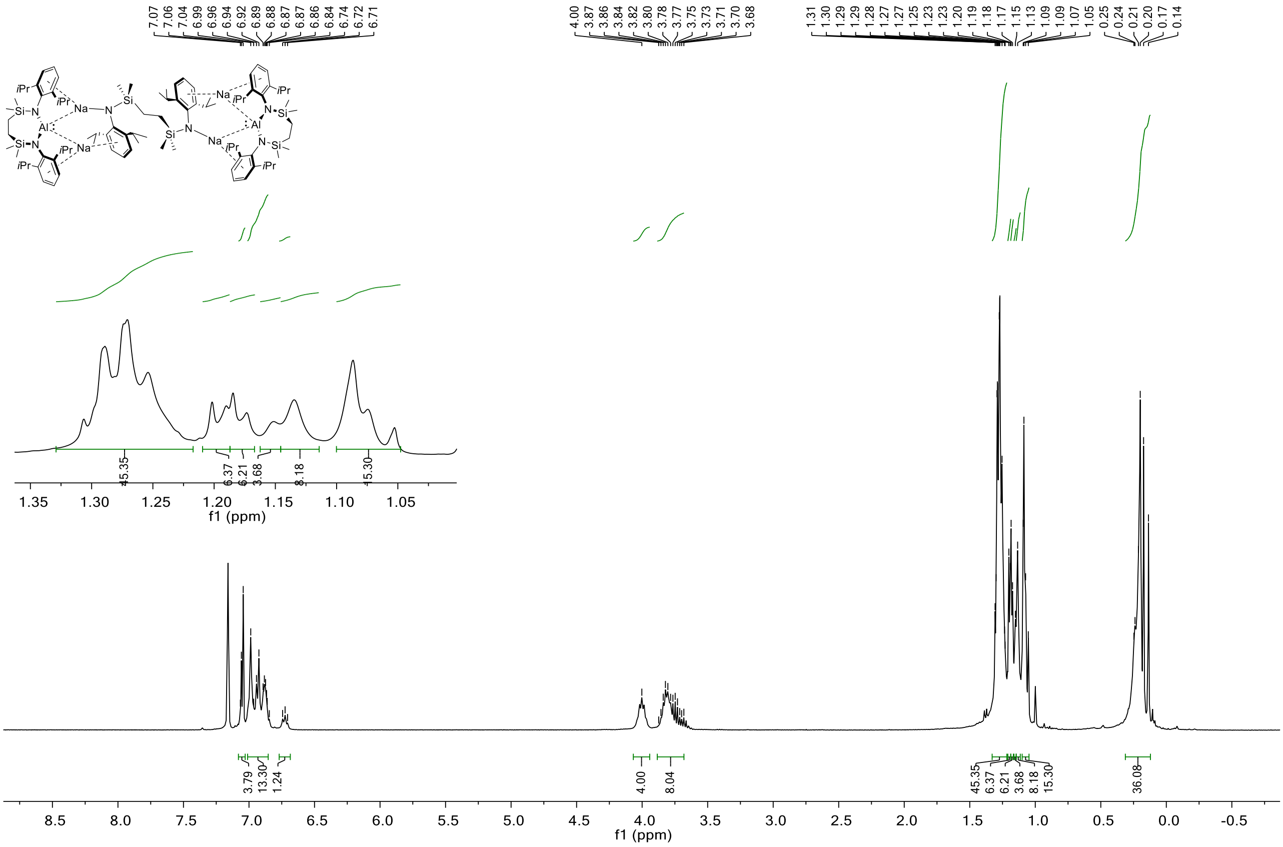

Figure S2: ^{13}C{^{1}H} NMR (101MHz, 298 K, d_6-benzene) spectrum of **14**.

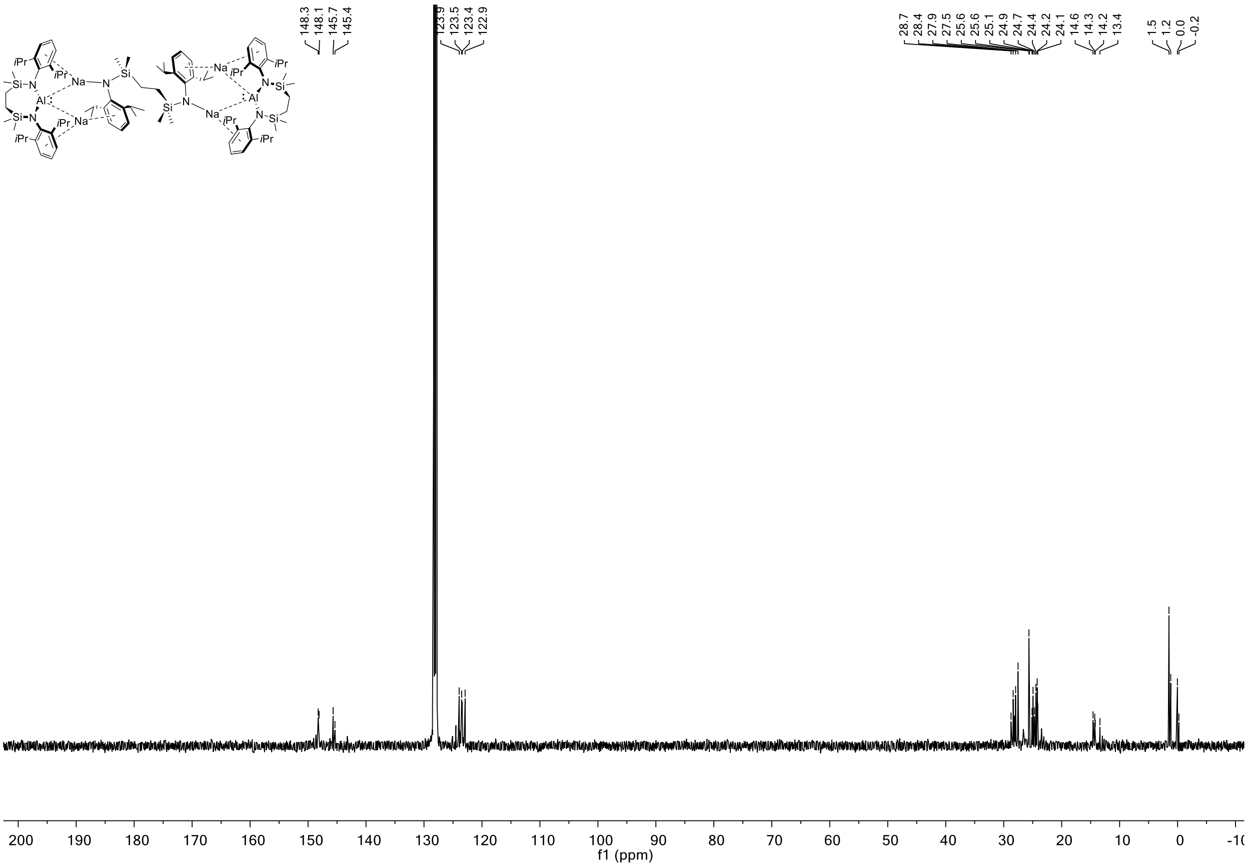

Figure S3: ^{1}H NMR (400MHz, 298 K, d_6-benzene) spectrum of **16.** *hexane.

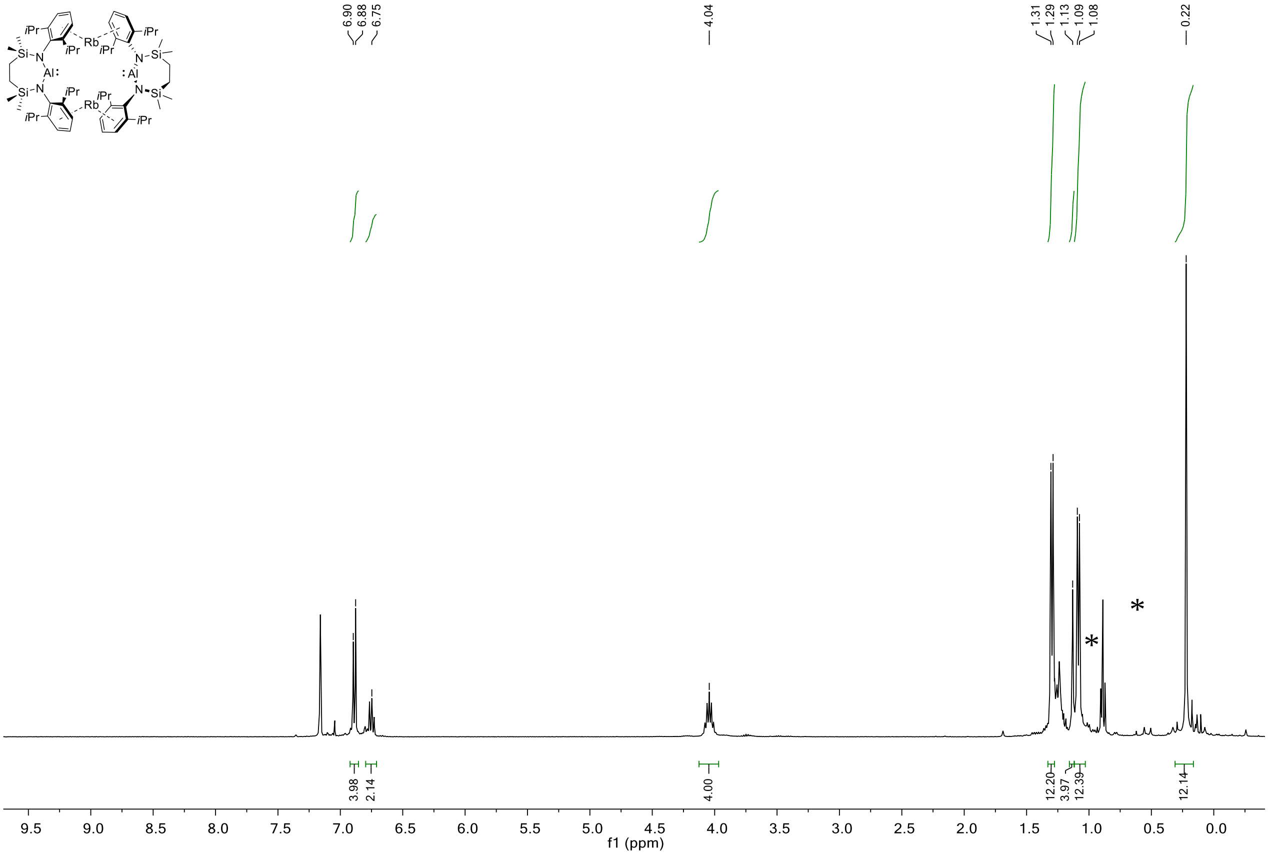

Figure S4: ^{13}C{^{1}H} NMR (101MHz, 298 K, d_6-benzene) spectrum of **16**. *hexane

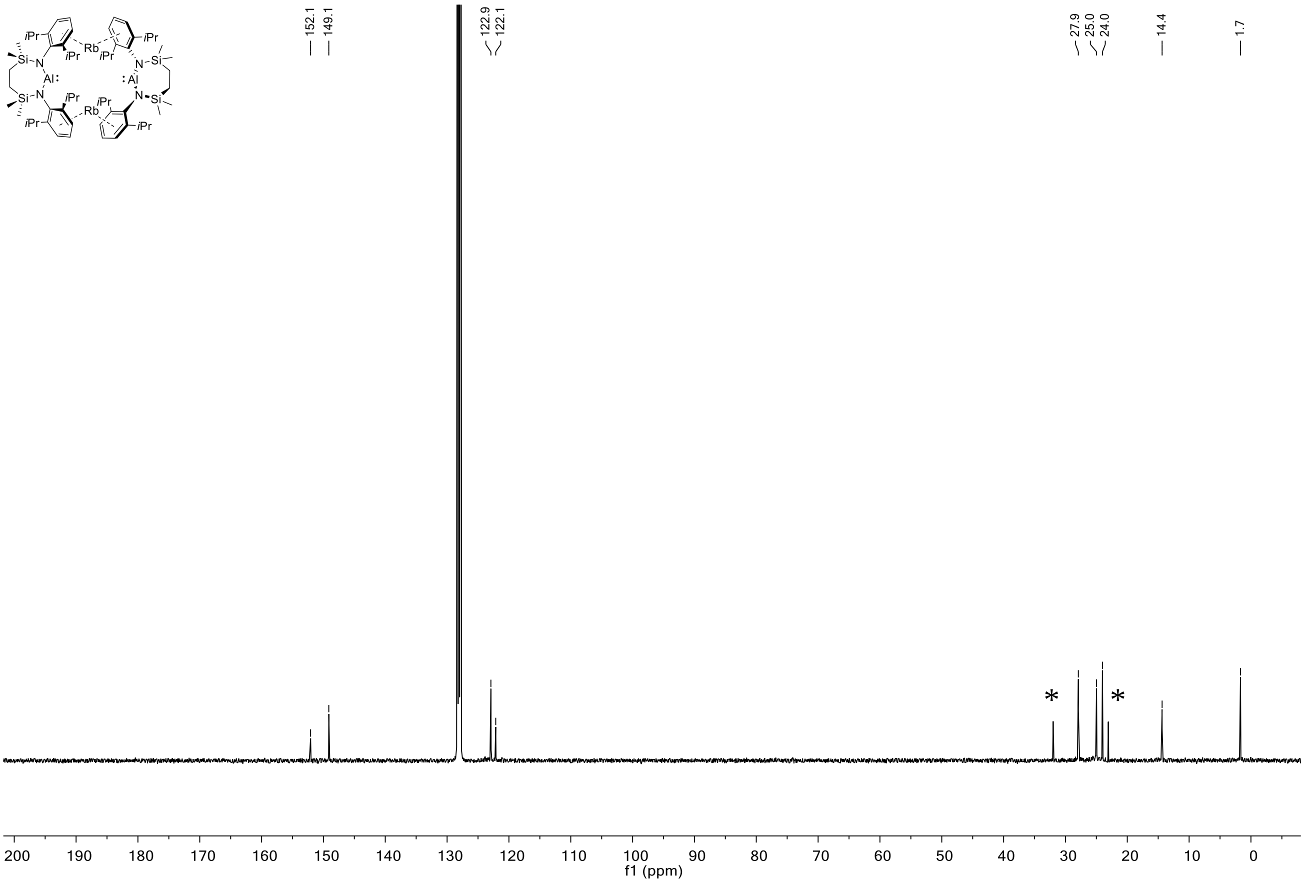

Figure S5: ^1H-^{13}C HSQC spectrum of **16**.

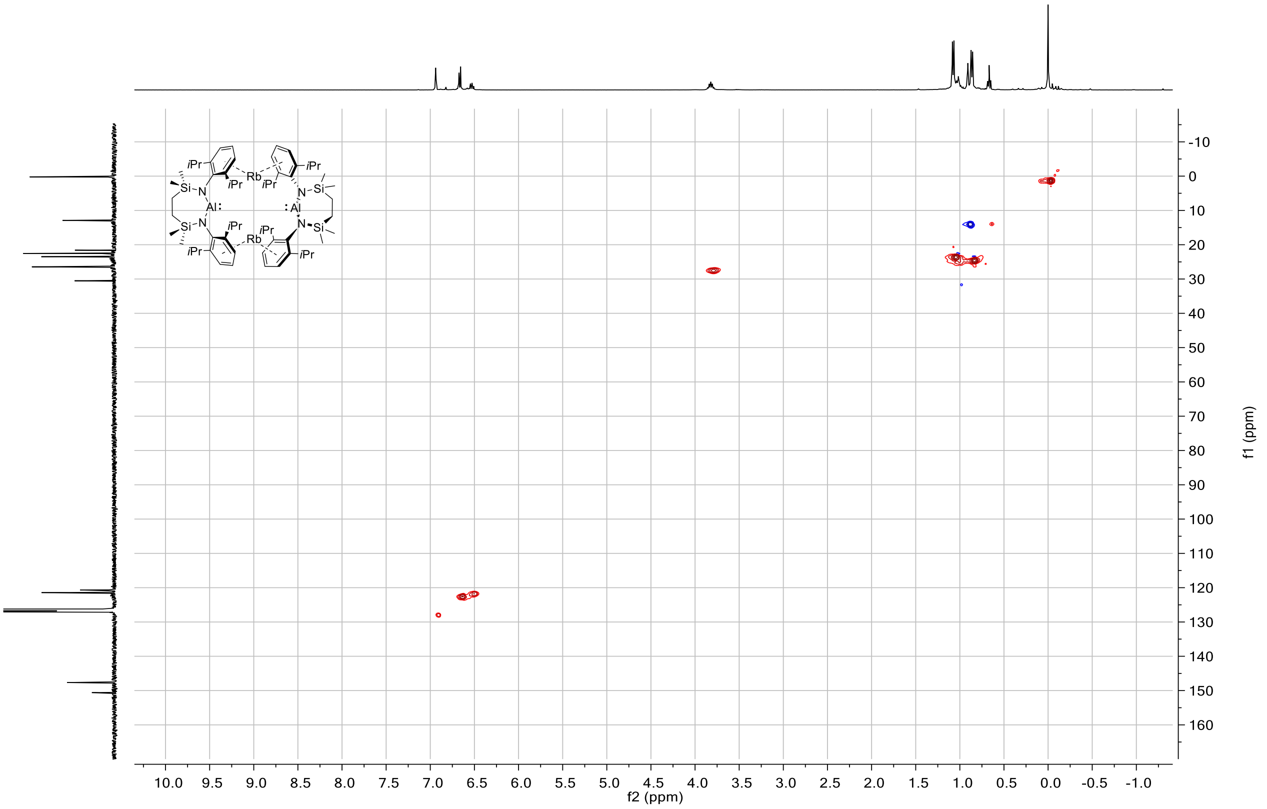

Figure S6: ^1H-^{13}C HMBC spectrum of **16**.

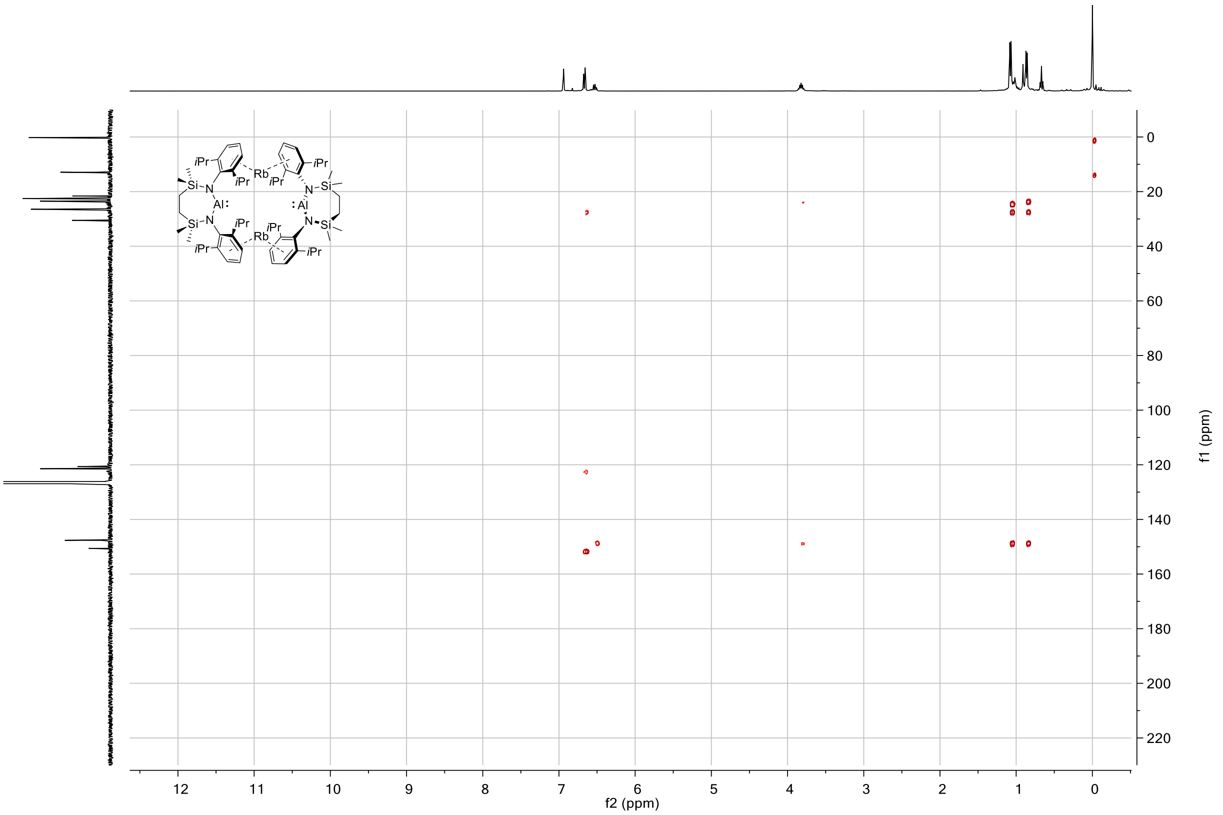

Figure S7: ^{1}H NMR (400MHz, 298 K, d_6-benzene) spectrum of **17.** *hexane

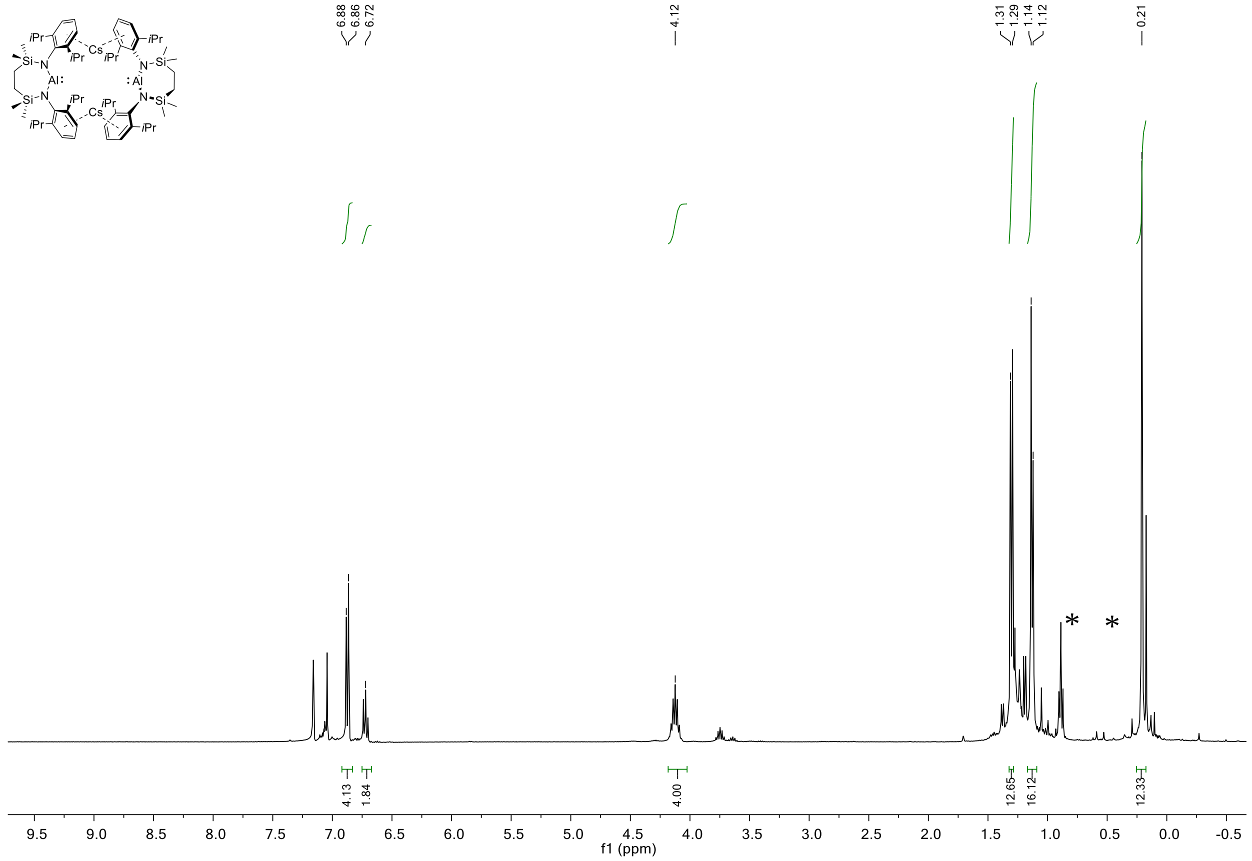

Figure S8: $^{13}C\{^{1}H\}$ NMR (101MHz, 298 K, d_6-benzene) spectrum of **17**. *hexane

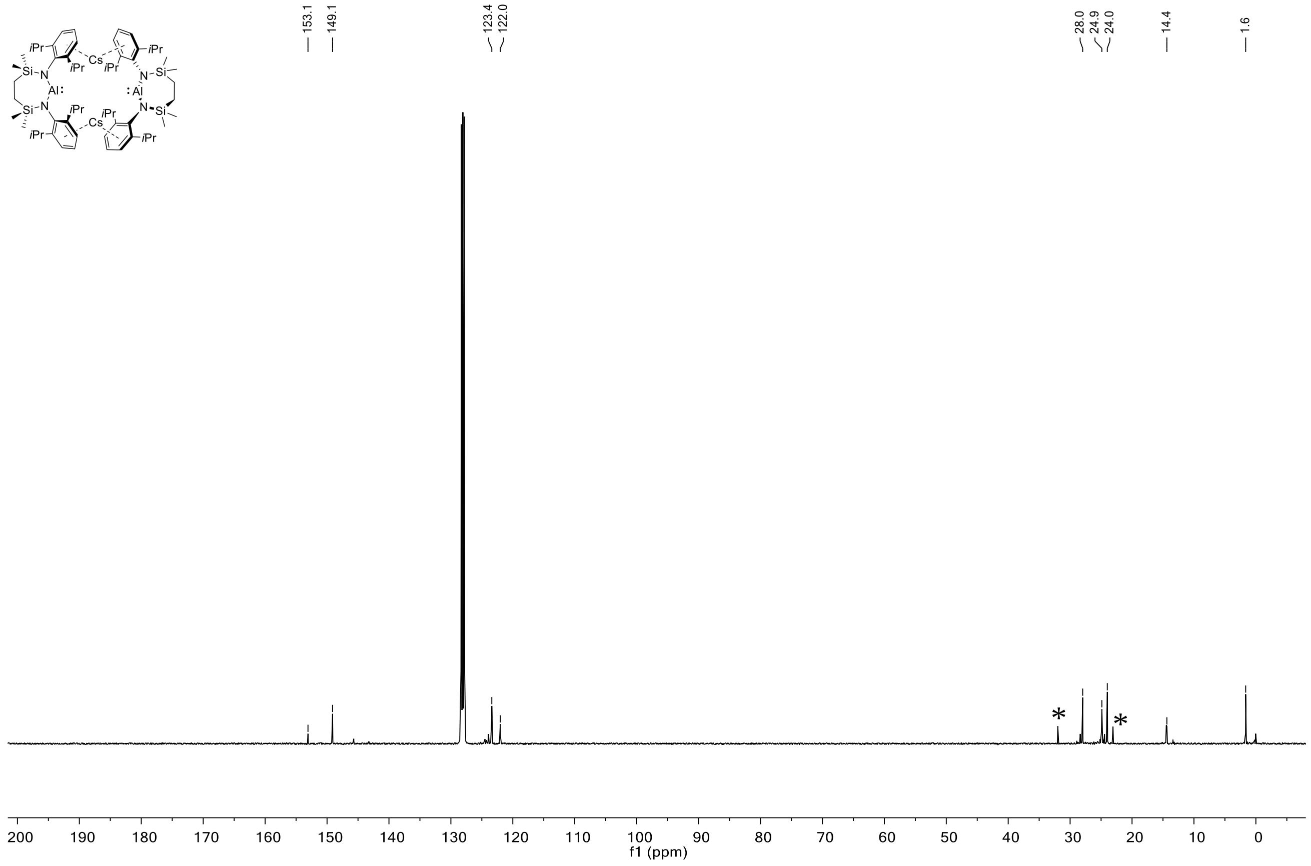

Figure S9: ^{1}H-^{13}C HSQC spectrum of **17**.

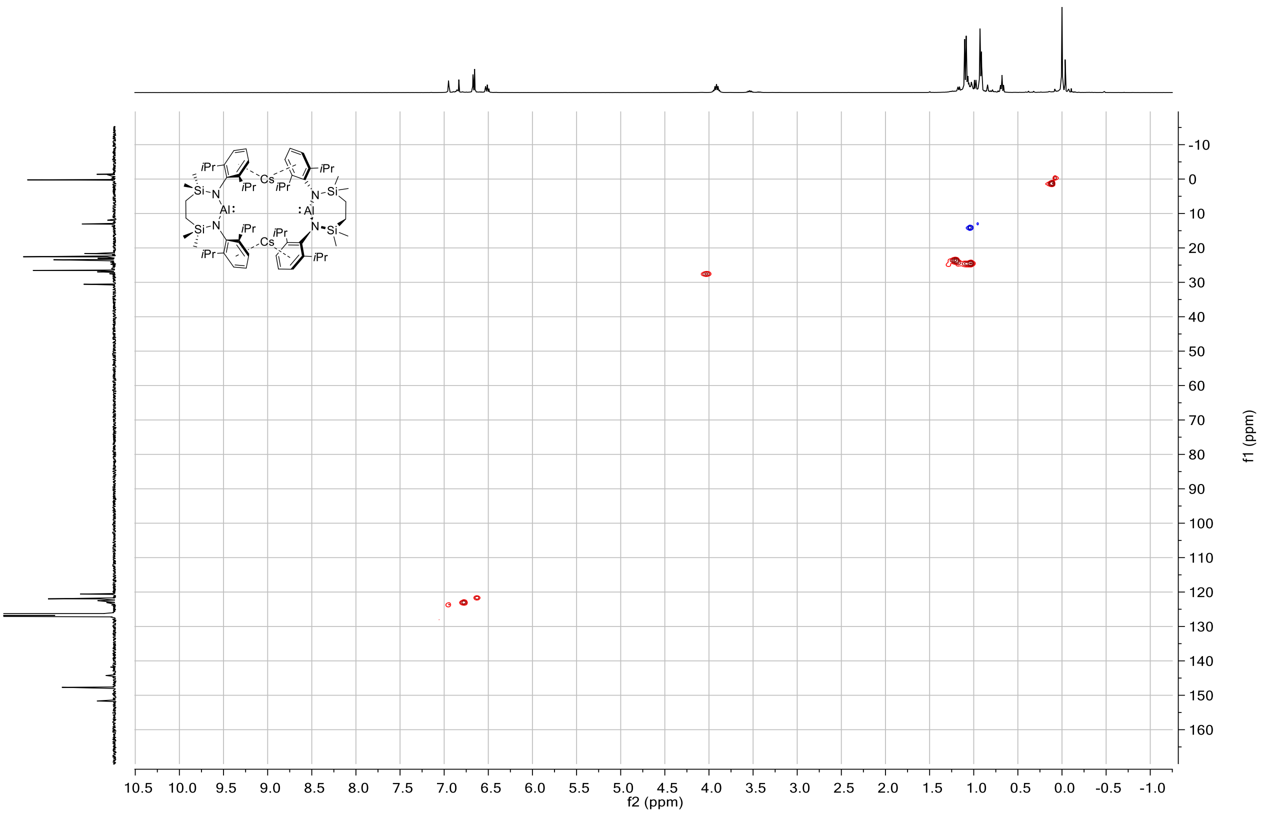

Figure S10: ^{1}H-^{13}C HMBC spectrum of **17**.

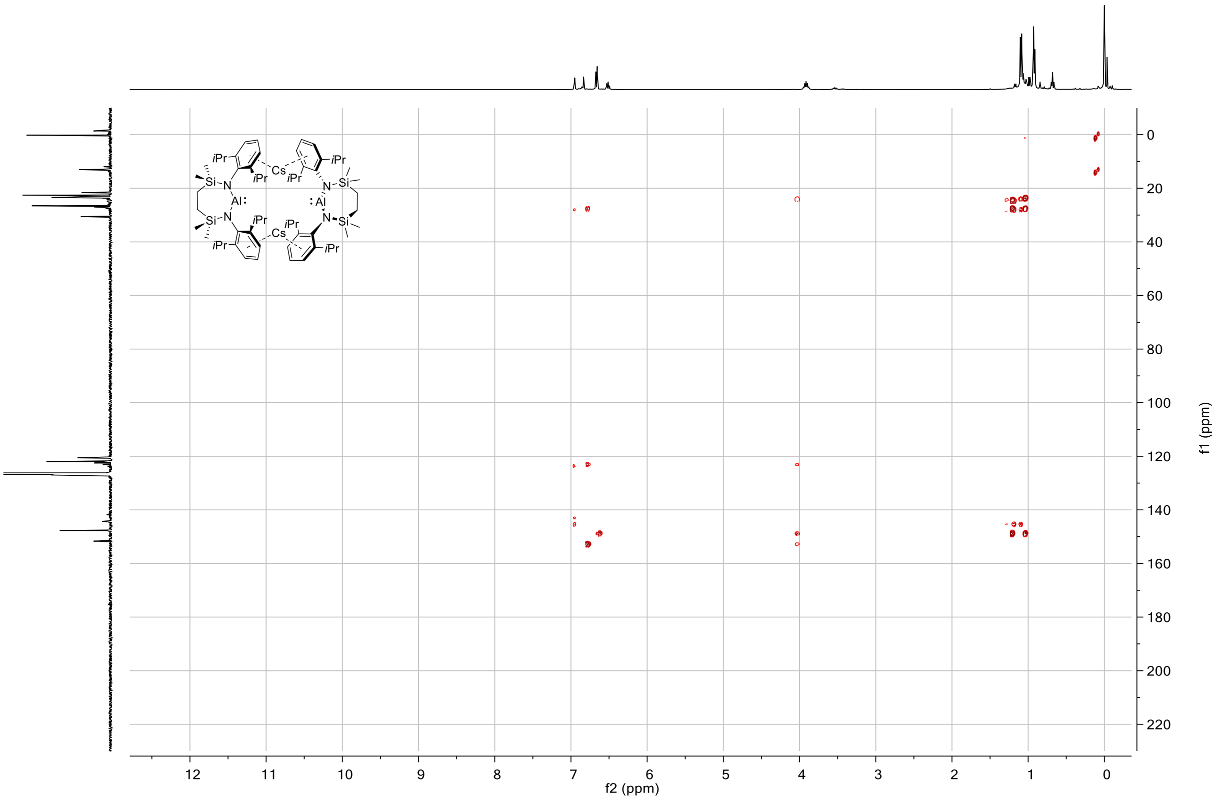

Figure S11: ^{1}H NMR (400MHz, 298 K, d_6-benzene) spectrum of the isolated crystals of **18**.

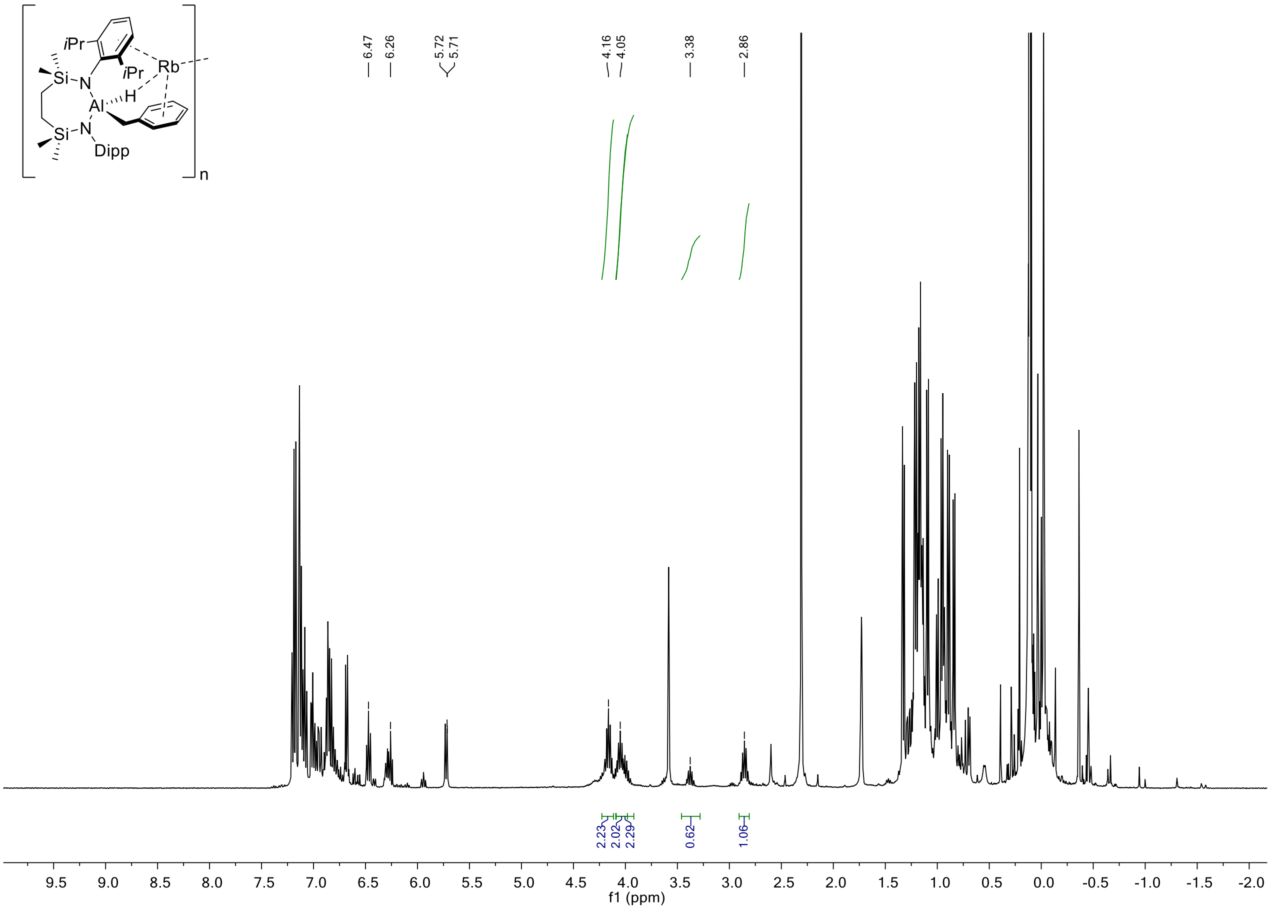

Figure S12: ^{1}H NMR (400MHz, 298 K, d$_8$-THF) spectrum of **19.** *benzene

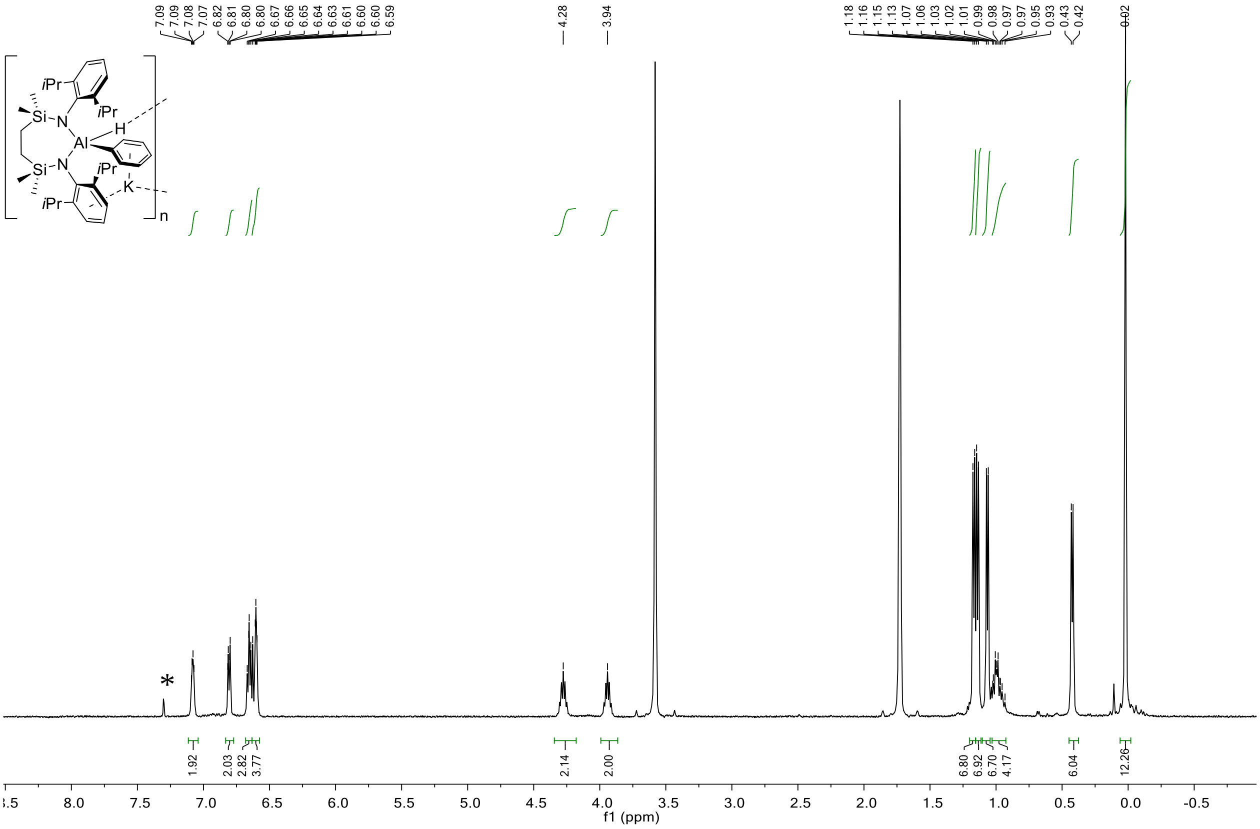

Figure S13: ^{13}C{^{1}H} NMR (101MHz, 298 K, d$_8$-THF) spectrum of **19**.

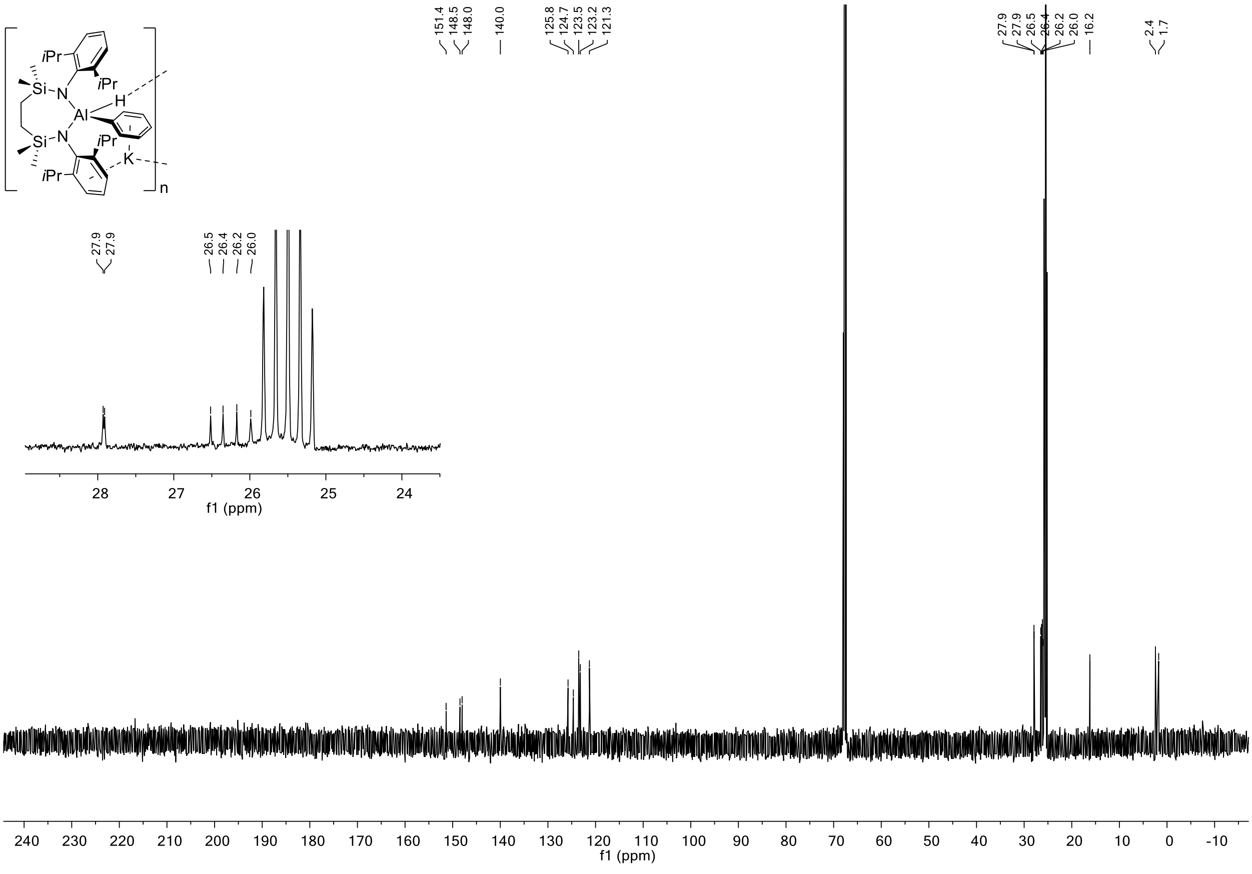

Figure S14: ^{1}H-^{13}C HSQC spectrum of **19**.

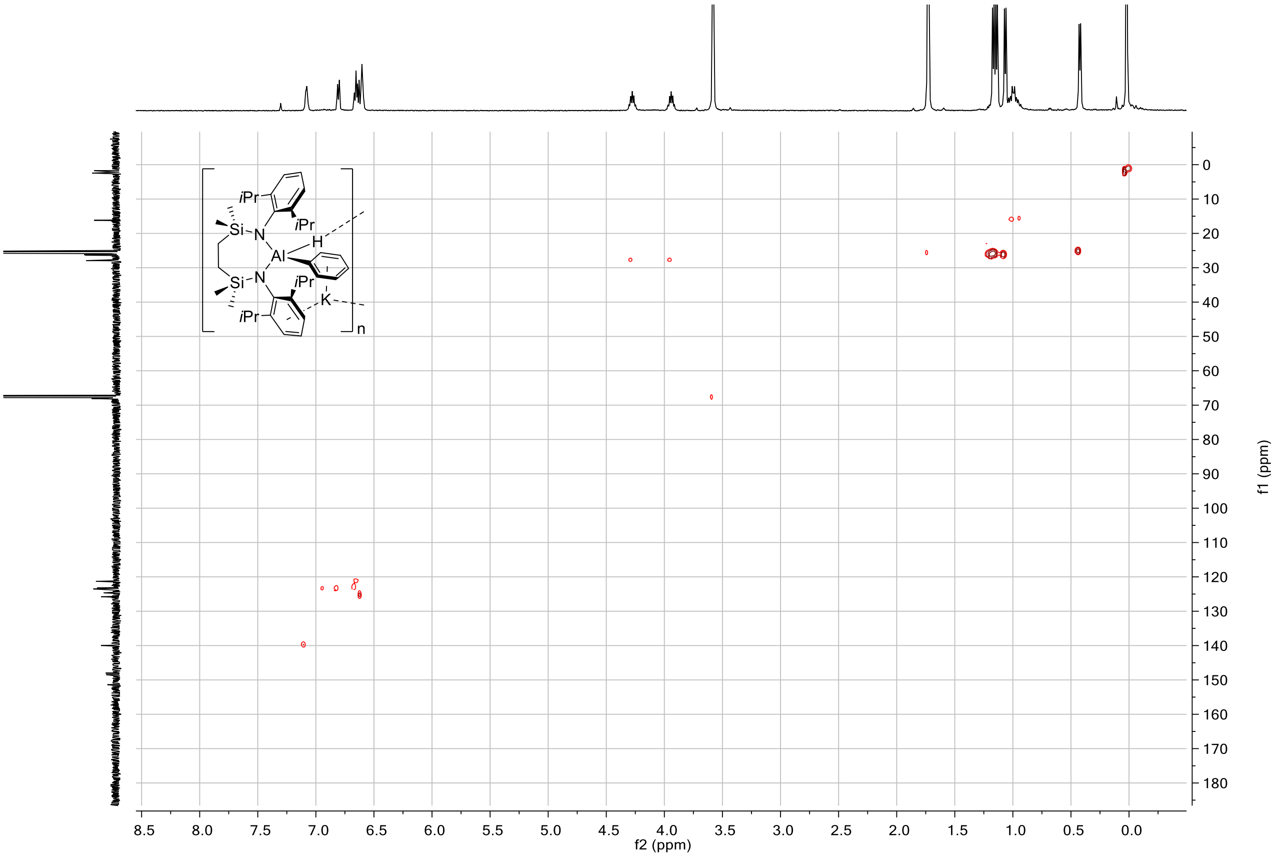

Figure S15: ^{1}H-^{13}C HMBC spectrum of **19**.

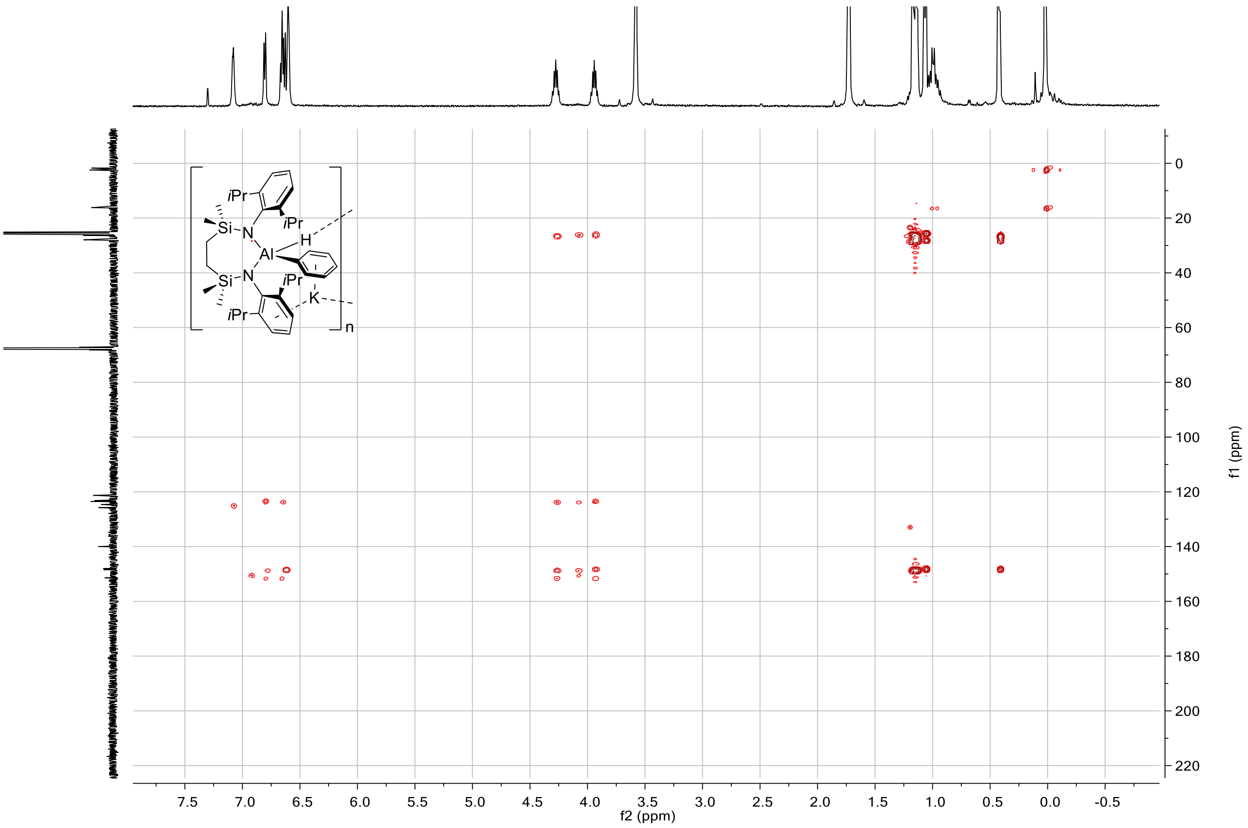

Figure S16: ^{1}H NMR (400MHz, 298 K, d_8-THF) spectrum of **20.** *benzene $^{+}$hexane $^{\times}$grease.

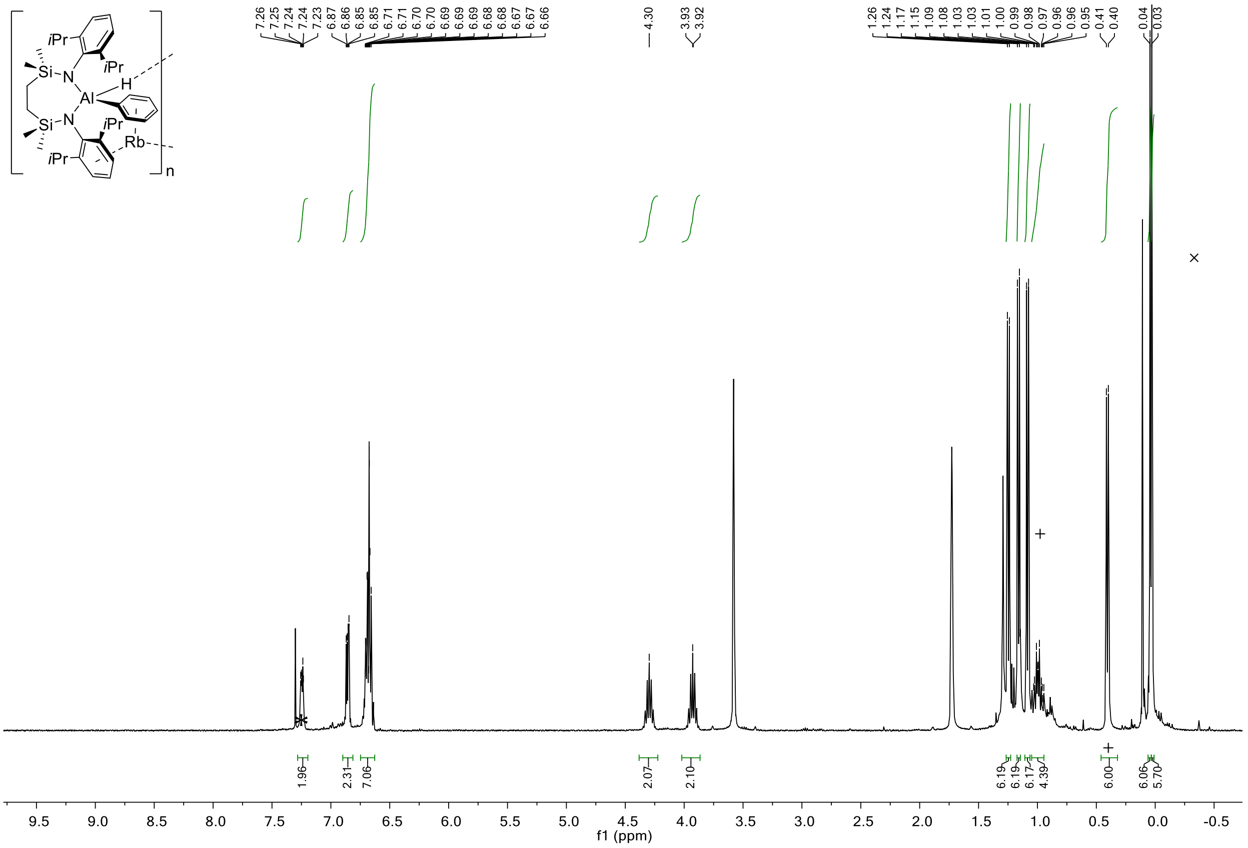

Figure S17: ^{13}C{^{1}H} NMR (101MHz, 298 K, d_8-THF) spectrum of **20**. $^{+}$hexane

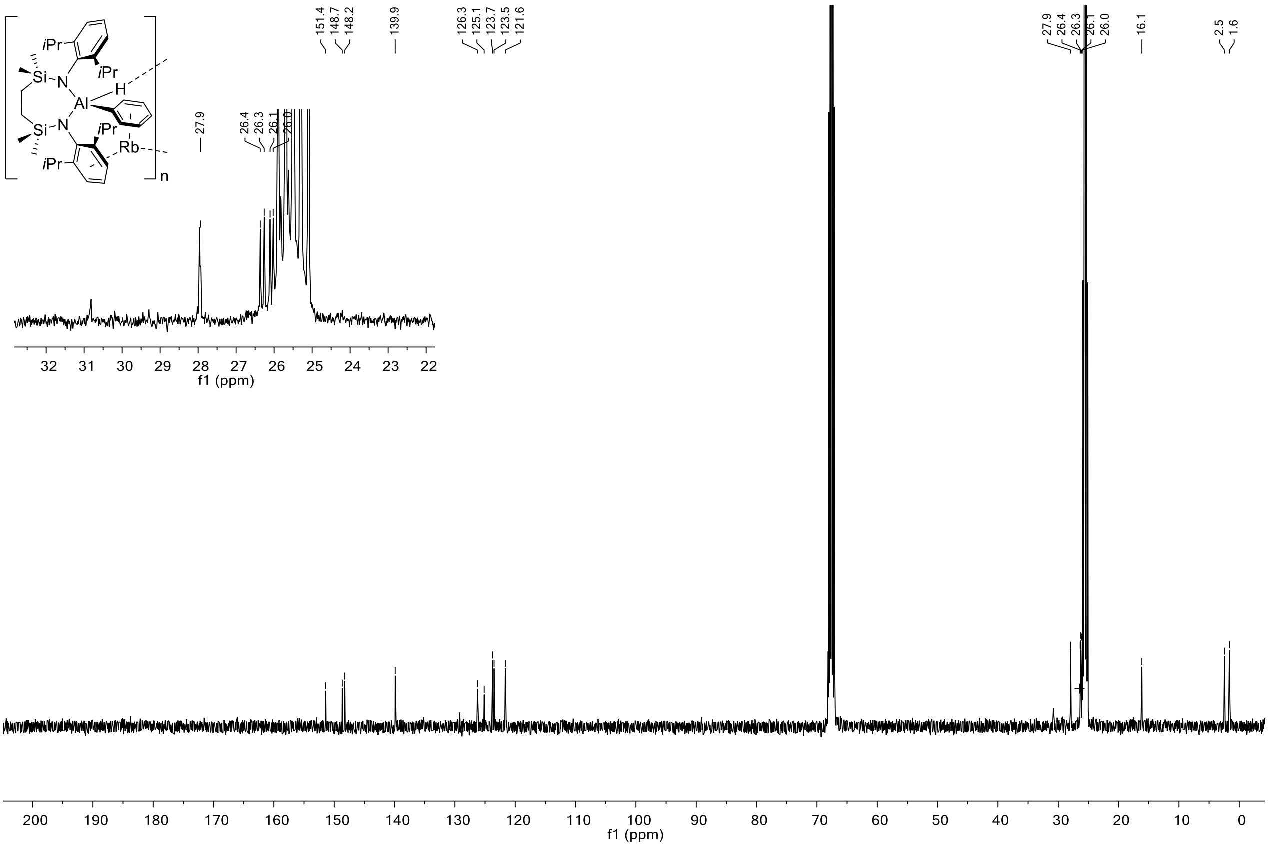

Figure S18: ^{1}H-^{13}C HSQC spectrum of **20**. $^{+}$hexane

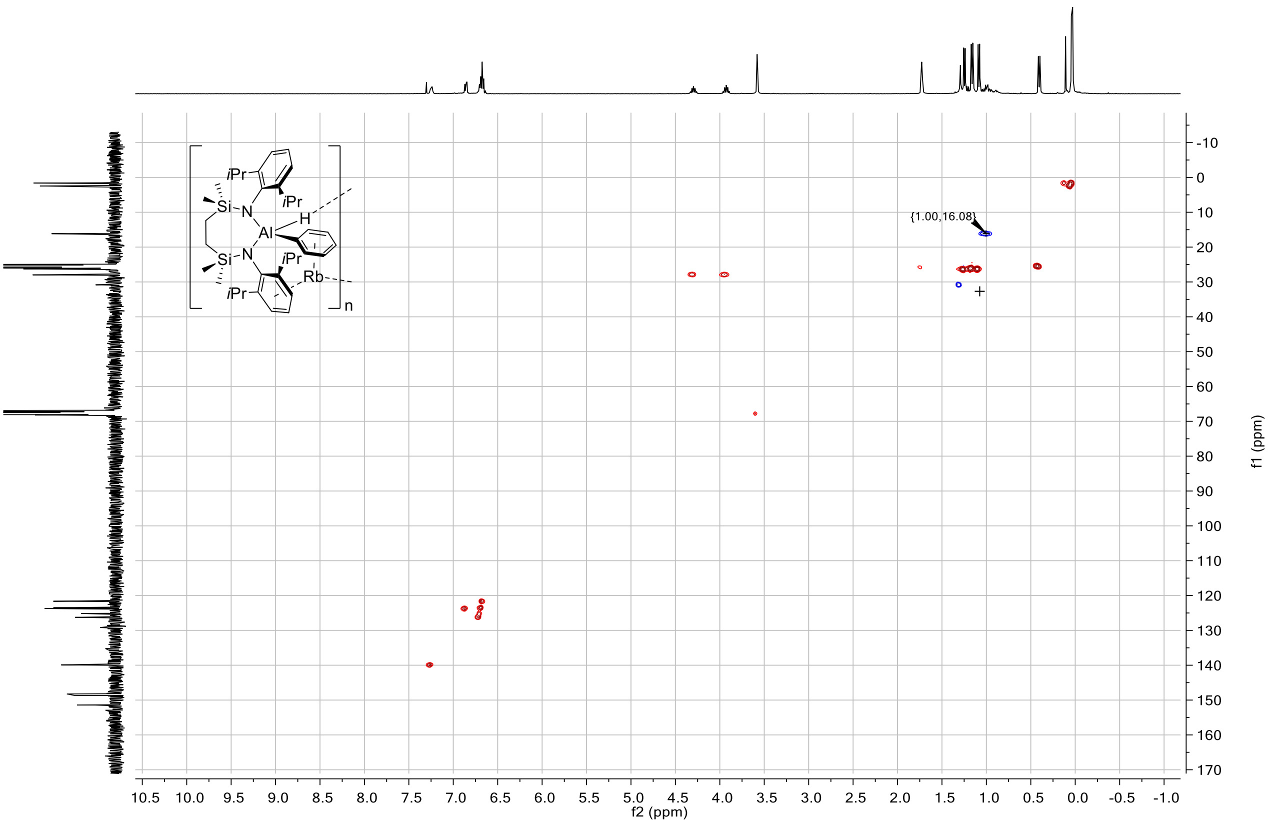

Figure S19: ^{1}H-^{13}C HMBC spectrum of **20**.

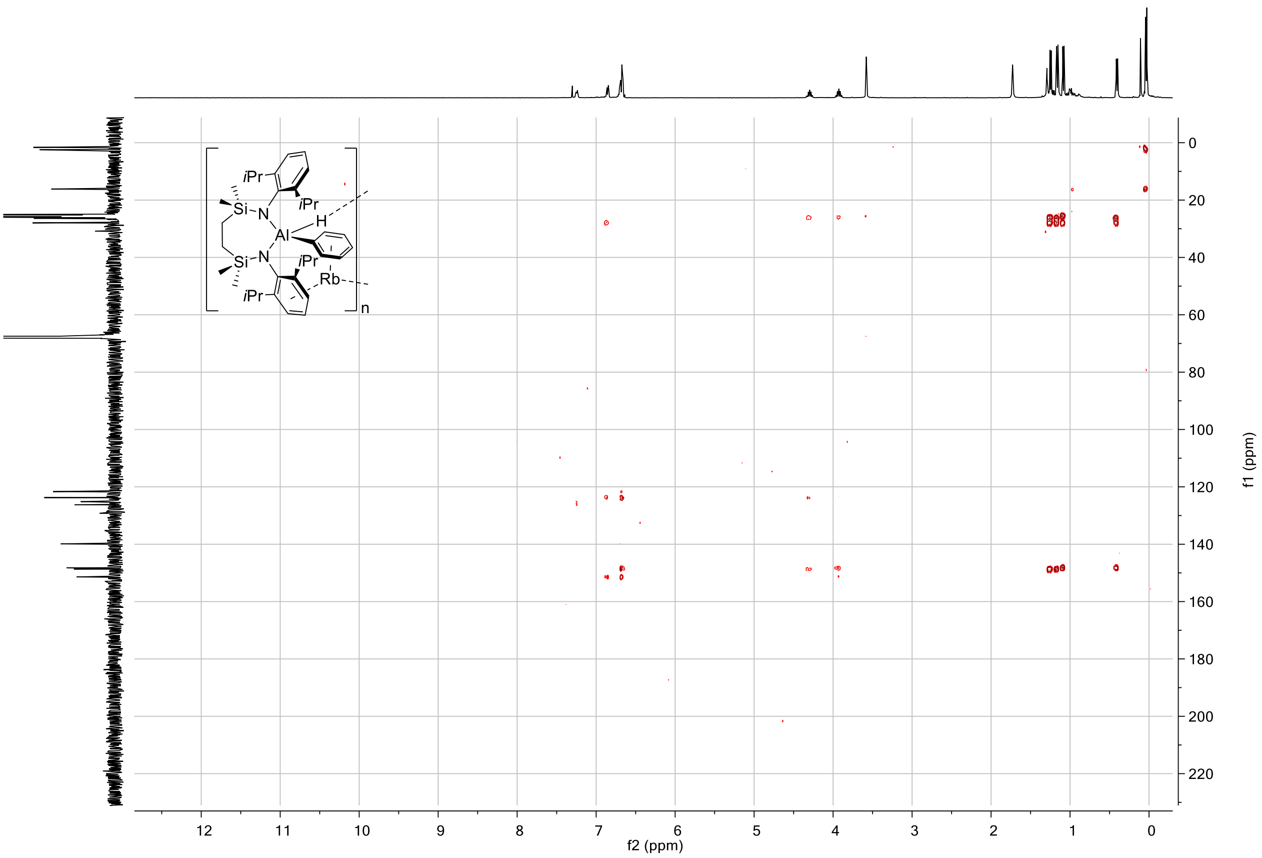

Figure S20: ^{1}H NMR (400MHz, 298 K, d$_8$-THF) spectrum of **21.** *benzene $^{+}$hexane $^{\times}$grease.

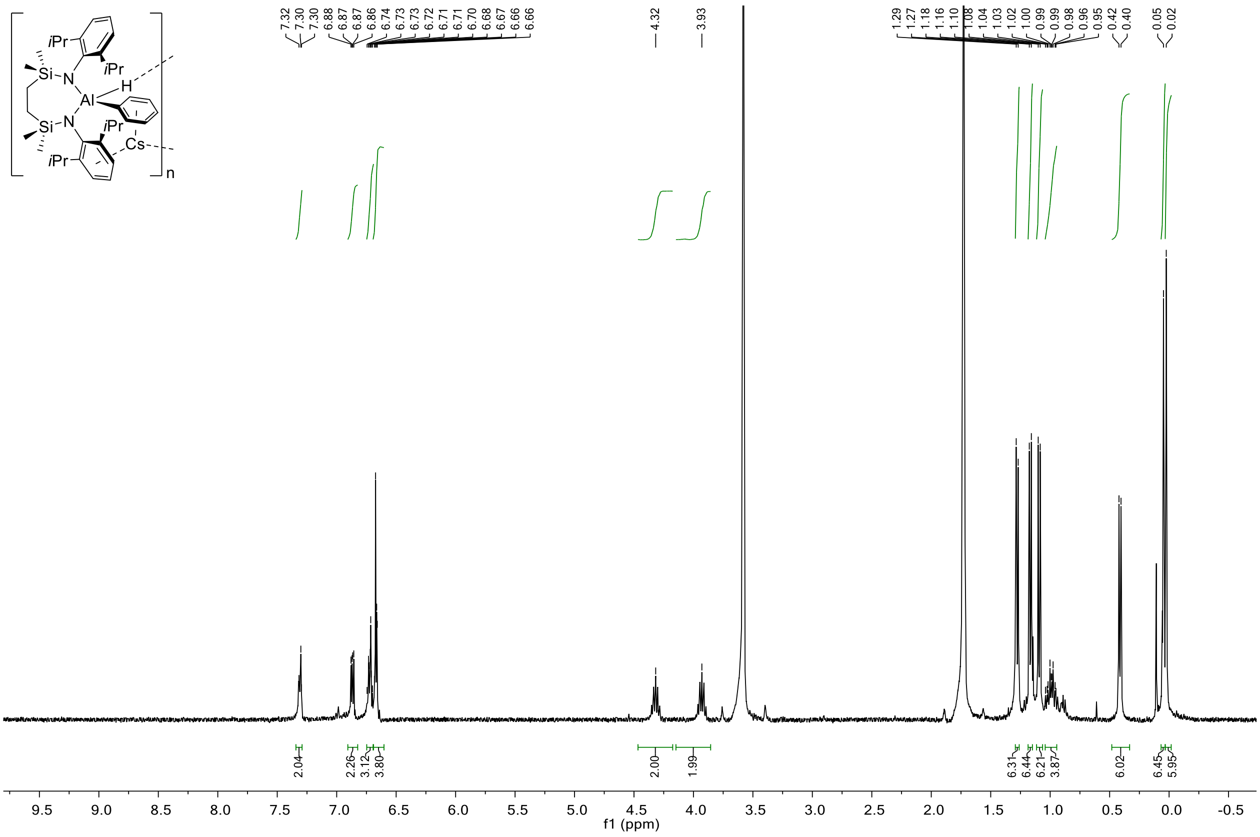

Figure S21: ^{13}C{^{1}H} NMR (101MHz, 298 K, d$_8$-THF) spectrum of **21**.

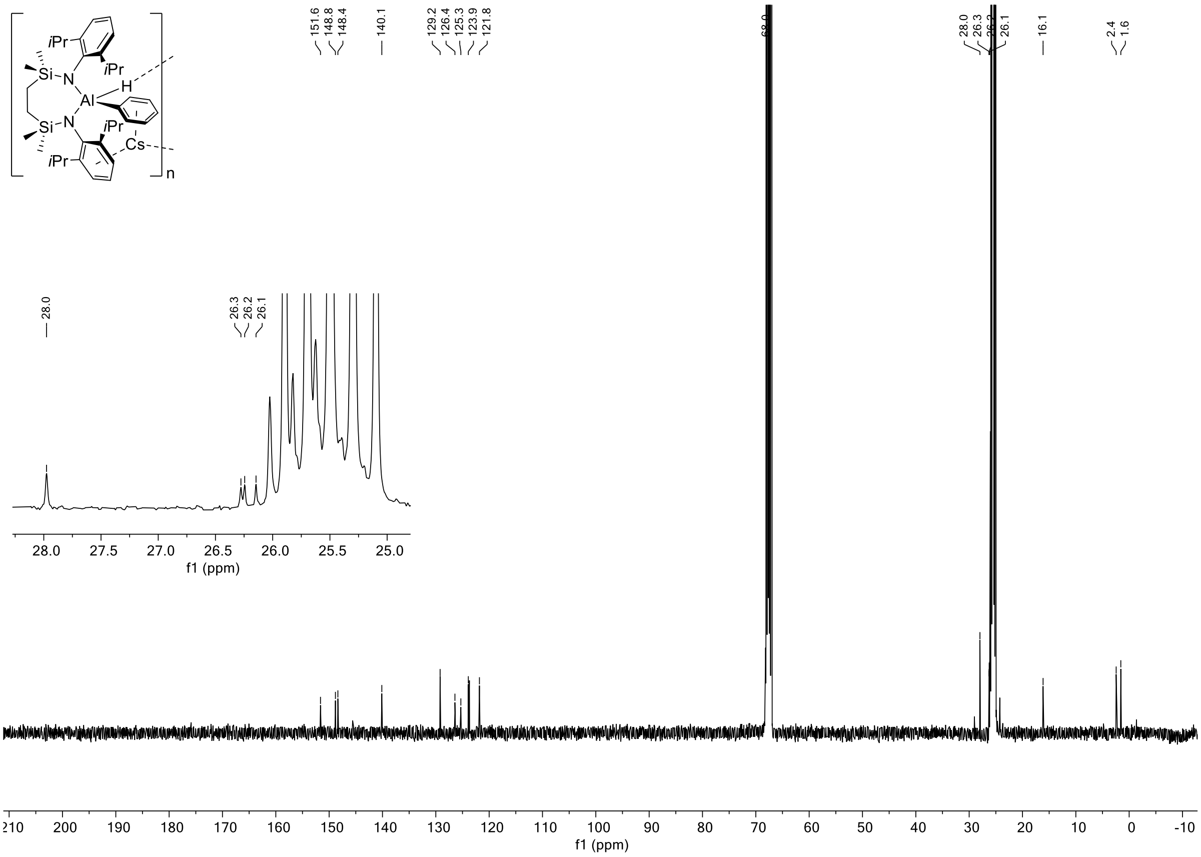

Figure S22: ^{1}H-^{13}C HSQC spectrum of **21**.

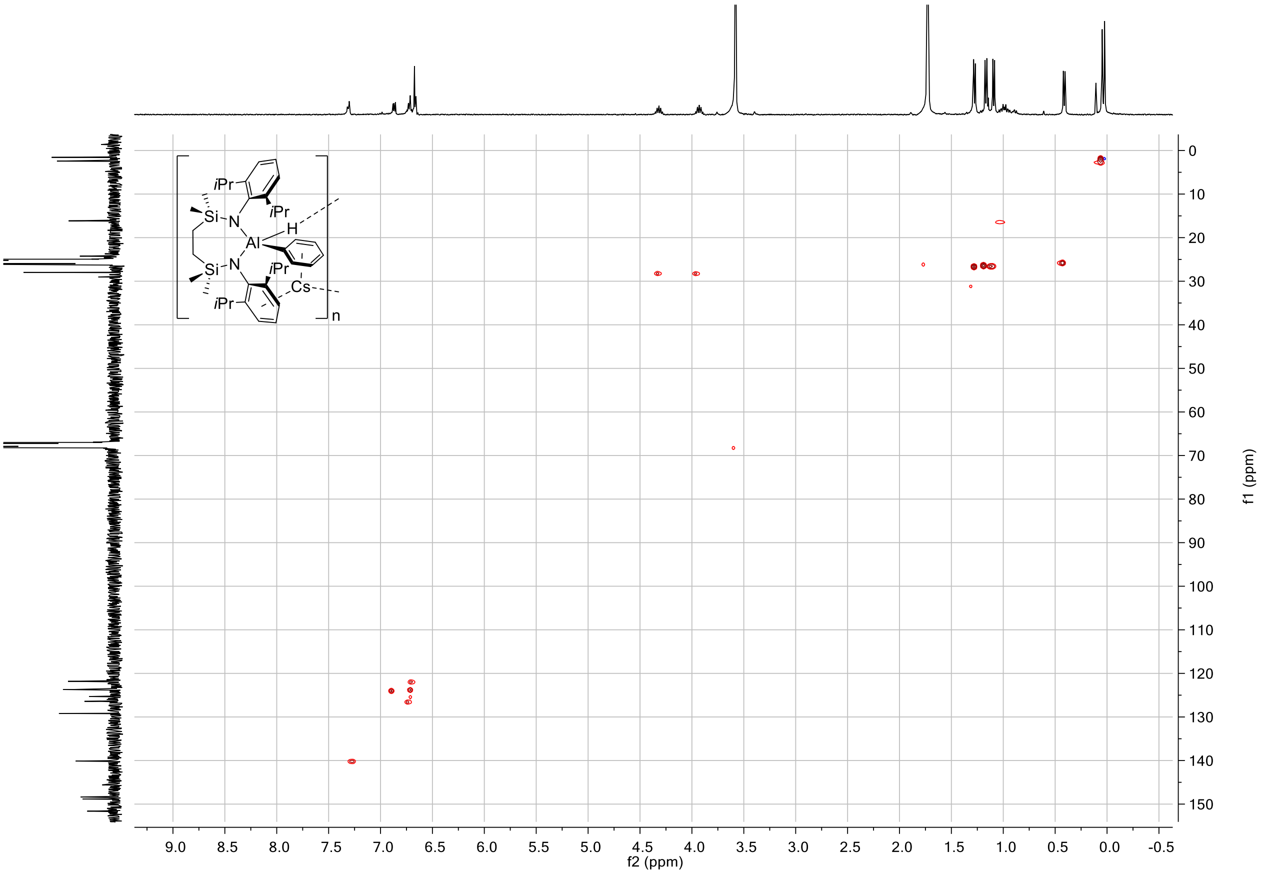

Figure S23 ^{1}H-^{13}C HMBC spectrum of **21**.

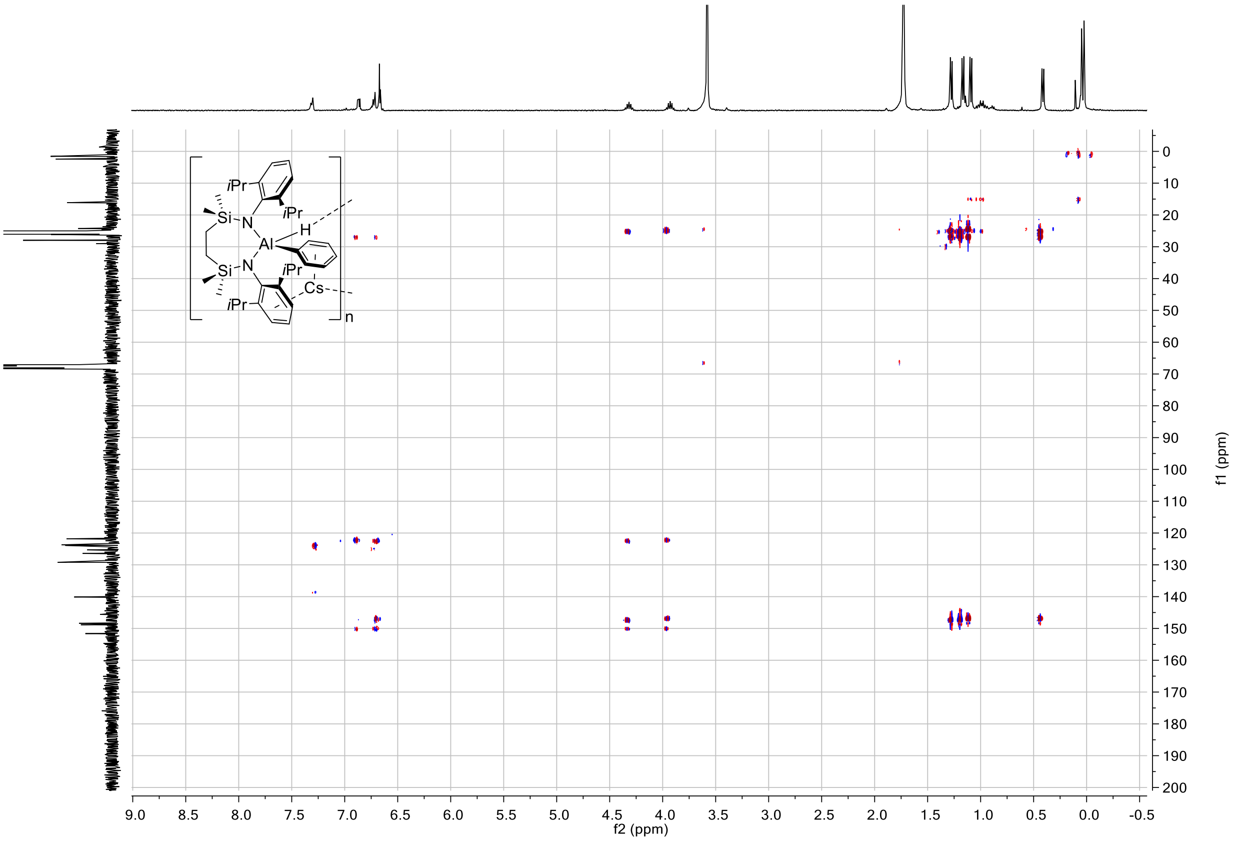

*Attempted kinetic study of the reaction [({SiN^{Dipp}}Al)Cs]$_2$ (**17**) with benzene*

Inside a J-Young's tube, [({SiN^{Dipp}}Al)Cs]$_2$ (**17**) (33 mg, 0.025 mmol) was dissolved with 0.4 mL of C_6D_6 to afford a bright yellow solution, protio-benzene (3.9 mg, 4.4 μl, 0.05 mmol) was then added *via* a micropipette. The reaction mixture was kept at 343 K inside an NMR spectrometer and monitored by ^{1}H NMR spectroscopy every 30 minutes over a period of 13 hours. No significant change was observed in the ^{1}H spectrum during the reaction monitoring.

Figure S24: Stacked ^{1}H NMR (400 MHz, 343 K, d_6-benzene) spectra of the **17**/benzene reaction mixture; bottom to top: initial spectrum of the reaction mixture and spectra recorded every 30 minutes thereafter.

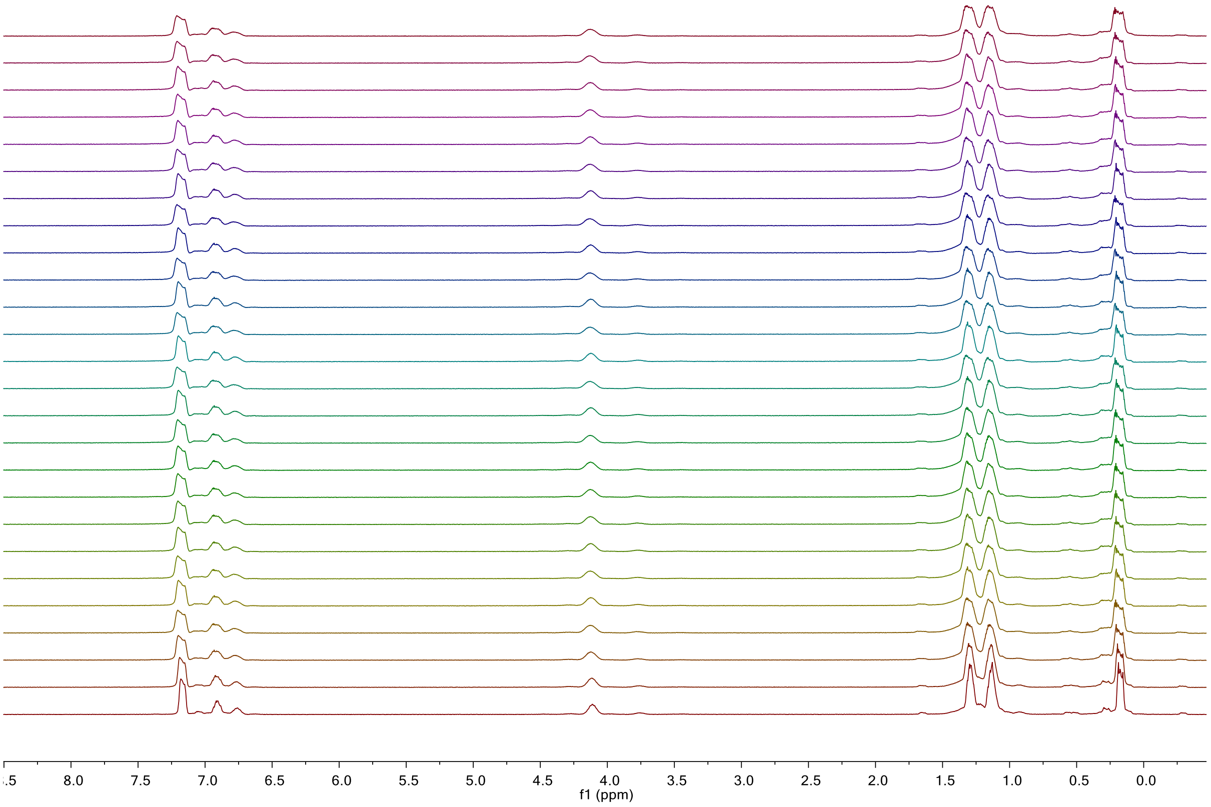

Single Crystal X-ray Diffraction Analysis

Single Crystal X–ray diffraction data for compounds **14** and **16 - 21** were collected on a SuperNova EosS2 diffractometer using either CuKα (λ = 1.54184 Å; **14**, **19**, **20** and **21**) or Mo Kα (λ = 0.71073; **16**, **17** and **18**) radiation. The crystals were maintained at 150 K during data collection. Using Olex2,[2] the structures were solved with the olex2.solve[3] structure solution program or ShelXT and refined with the ShelXL[4] refinement package using Least Squares minimisation. Noteworthy points follow, and where disorder has been modelled, both distance and ADP restraints have been employed, on merit, in these regions to assist convergence.

The asymmetric unit of **14** contains half of a dimer and two independent hexane halves. The remainders of each fragment arise via crystallographic inversion symmetry. There are some low-intensity spurious, residual electron-density peaks which reflect that the crystal, while visually perfect, was (in diffraction terms) of very good quality, albeit not premium. This manifested as streaking of some peaks and evidence of, perhaps, a small crack in the sample. The integration mask was, therefore, adjusted during data reduction.

There is one disordered hexane guest (55:45) in the asymmetric unit of **16**, which was refined with the inclusion of distance and APP restraints. The hydrogen atoms attached to C11, C26, C38 and C59 were located and refined at a distance of 0.98 Å from the relevant parent atom.

In addition to one molecule of the caesium complex, the asymmetric unit in **17** also contains one molecule of hexane. The latter was treated for 57:43 disorder.

In **18**, the asymmetric unit comprises a monomer which gives rise to 1-D polymers which propagate along the *b* axis in the gross structure. Si2 and C15-C18 were treated for 87:13 disorder.

67:33 disorder was modelled for N2, Si2, C16, C17 and C18 in the asymmetric unit of **19**. Once again, the gross structure is dominated by 1-D polymers.

The contents of the asymmetric unit in the structure of **20** also propagate to generate 1-D polymers, parallel to the *b* axis, in the gross structure. Aluminium bound hydrogen atoms were located and refined without restraints. The data were addressed for sample twinning (180° rotation about *b*) at the point of integration and refinement was effected for a 2-component twin.

The data set for **21** was also representative of a twinned crystal (180° rotation about *b*) and this was addressed at the point of integration. As for **18, 19** and **20,** the asymmetric unit is a monomer which gives rise to 1-D polymers in the gross structure. Hydrogen atoms bound to aluminium centres were located and refined without restraints.

Table S1: Crystal data and structure refinement for compounds **14**, **16**, **17** and **18**.

Identification code	**14**	**16**	**17**	**18**
Empirical formula	$C_{51}H_{87}AlN_3Na_2Si_3$	$C_{66}H_{114}Al_2N_4Rb_2Si_4$	$C_{66}H_{114}Al_2Cs_2N_4Si_4$	$C_{37}H_{58}AlN_2RbSi_2$
Formula weight	899.46	1300.87	1395.75	699.48
Crystal system	triclinic	monoclinic	monoclinic	monoclinic
Space group	P–1	$P2_1/c$	$P2_1/c$	$P2_1/n$
a/ Å	10.51871(18)	16.7763(3)	17.0215(4)	17.1990(4)
b/ Å	13.1772(3)	17.3329(3)	17.2252(6)	10.6411(2)
c/ Å	21.5875(5)	26.5344(4)	26.7216(8)	21.7562(5)
α/ °	105.1605(19)	90	90	90
β/ °	93.2082(16)	101.481(2)	101.274(3)	102.918(2)
γ/ °	101.1801(16)	90	90	90
U/ Å^3	2815.20(10)	7561.3(2)	7683.6(4)	3880.96(15)
Z	2	4	4	4
ρ_{calc}/ g cm^{-3}	1.061	1.143	1.207	1.197
μ/ mm^{-1}	1.319	1.418	1.071	1.386
F(000)	982.0	2776.0	2920.0	1488.0
Crystal size/ mm^3	0.26 × 0.105 × 0.065	0.471 × 0.349 × 0.162	0.267 × 0.155 × 0.092	0.246 × 0.093 × 0.061
2θ range for data collection/°	7.118 to 145.744	6.648 to 60.792	6.644 to 60.584	6.686 to 60.968
Index ranges	$-13 \leq h \leq 9$, $-16 \leq k \leq 16$, $-25 \leq l \leq 26$	$-23 \leq h \leq 23$, $-24 \leq k \leq 22$, $-36 \leq l \leq 36$	$-22 \leq h \leq 22$, $-23 \leq k \leq 21$, $-35 \leq l \leq 30$	$-24 \leq h \leq 22$, $-14 \leq k \leq 14$, $-30 \leq l \leq 28$
Reflections collected	25761	94208	45340	38025
Independent reflections, R_{int}	11065, 0.0332	20530, 0.0434	19683, 0.0291	10385, 0.0409
Data/restraints/parameters	11065/0/561	20530/178/829	19683/201/785	10385/117/451
Goodness–of–fit on F^2	1.031	1.030	1.043	1.036
Final R1, wR2 [I>=2σ(I)]	0.0535, 0.1382	0.0412, 0.0828	0.0402, 0.0810	0.0433, 0.0851
Final R1, wR2 [all data]	0.0635, 0.1460	0.0703, 0.0925	0.0654, 0.0901	0.0744, 0.0955
Largest diff. peak/hole/ e Å^{-3}	0.73/–0.39	0.42/–0.40	0.52/–0.47	0.47/–0.44

Table S2: Crystal data and structure refinement for compounds **19**, **20** and **21**.

Identification code	**19**	**20**	**21**
Empirical formula	$C_{36}H_{56}AlKN_2Si_2$	$C_{72}H_{112}Al_2N_4Rb_2Si_4$	$C_{72}H_{112}Al_2Cs_2N_4Si_4$
Formula weight	639.08	1370.91	1465.79
Crystal system	monoclinic	triclinic	triclinic
Space group	$P2_1/c$	P–1	P–1
a/ Å	11.7099(4)	11.7623(4)	11.8376(4)
b/ Å	12.9316(5)	13.0073(3)	13.0926(3)
c/ Å	25.0396(8)	24.9819(4)	25.0313(3)
α/ °	90	90.4636(15)	90.330(1)
β/ °	98.151(3)	98.128(2)	98.135(2)
γ/ °	90	91.451(2)	91.695(3)
U/ Å^3	3753.4(2)	3782.26(15)	3838.56(16)
Z	4	2	2
ρ_{calc}/ g cm^{-3}	1.131	1.204	1.268
μ/ mm^{-1}	2.257	2.811	8.522
F(000)	1384.0	1456.0	1528.0
Crystal size/ mm^3	0.129 × 0.071 × 0.035	0.191 × 0.091 × 0.034	0.129 × 0.086 × 0.035
2θ range for data collection/°	7.132 to 146.8	7.594 to 145.71	7.548 to 146.97
Index ranges	$-14 \le h \le 14$, $-15 \le k \le 15$, $-30 \le l \le 25$	$-14 \le h \le 14$, $-15 \le k \le 16$, $-30 \le l \le 30$	$-14 \le h \le 14$, $-16 \le k \le 12$, $-30 \le l \le 30$
Reflections collected	20520	16692	18379
Independent reflections, R_{int}*	7414, 0.0479	16692	18379
Data/restraints/parameters	7414/129/442	16692/0/790	18379/0/790
Goodness–of–fit on F^2	1.039	0.887	0.875
Final $R1$, $wR2$ [I>=2σ(I)]	0.0600, 0.1521	0.0354, 0.0745	0.0297, 0.0607
Final $R1$, $wR2$ [all data]	0.0775, 0.1668	0.0521, 0.0776	0.0426, 0.0628
Largest diff. peak/hole/ e Å^{-3}	0.55/–0.40	0.65/–0.41	0.80/–0.57

* R_{int} not noted for twinned structures.

Computational Details

Methodology

DFT calculations were run with Gaussian 16 (C.01).[5] The Na, Al, Si, K, Cs and Rb centres were described with the Stuttgart RECPs and associated basis sets,[6] and 6-31G** basis sets were used for all other atoms (BS1).[7] A polarization function was also added to Al (ζ_d = 0.190), Si (ζ_d = 0.284), K (ζ_d = 1.000), Rb (ζ_d = 0.491) and Cs (ζ_d = 0.306). Initial BP86[8] optimizations were performed using the 'grid = ultrafine' option, with all stationary points being fully characterized via analytical frequency calculations as minima (all positive eigenvalues) or transition states (one negative eigenvalue). All energies were recomputed with a larger basis set featuring 6-311++G** basis sets on all atoms, with the exception of Rb and Cs, where def2-TVZP was used (BS2). Corrections for the effect of benzene (ε = 2.2706) solvent were run using the polarizable continuum model and BS1, using the keyword "scrf=benzene" within Gaussian.[9] Single-point dispersion corrections to the BP86/BS1 results employed Grimme's D3 parameter set with Becke-Johnson damping as implemented in Gaussian.[10]

Breakdown of Energy Contributions

The following tables detail the evolution of the relative energies as the successive corrections to the initial SCF energy are included. Terms used are:

$\mathbf{\Delta E_{BSI}}$	SCF energy computed with the BP86 functional with BS1
$\mathbf{\Delta H_{BSI}}$	Enthalpy at 0 K with BS1
$\mathbf{\Delta G_{BSI}}$	Free energy at 298.15 K and 1 atm with BS1
$\mathbf{\Delta G_{BSI/\mathit{bnz}}}$	Free energy corrected for benzene solvent with BS1
$\mathbf{\Delta G_{BSI/bnz+D3BJ}}$	Free energy corrected for benzene and dispersion effects with BS1
$\mathbf{\Delta E_{BS2}}$	SCF energy computed with the BP86 functional with BS2
$\mathbf{\Delta G_{\mathit{bnz}}}$	Free energy corrected for basis set (BS2), dispersion effects and benzene solvent

In each case the final data used in the main article are highlighted in bold. Free energies are quoted in kcal mol^{-1}, and include all three single point corrections (for BS2, solvation and dispersion).

Table S3: Relative energies for computed structures (in kcal mol^{-1}). Data in bold is at the same level as free energies used in the text. Free energies are quoted relative to $\mathbf{A^{Na}}$.

	ΔE_{BSI}	ΔH_{BSI}	ΔG_{BSI}	$\Delta G_{BSI/bnz}$	$\Delta G_{BSI/bnz+D3BJ}$	ΔE_{BS2}	ΔG_{bnz}
$\mathbf{A^{Na}}$	0.0	0.0	0.0	0.0	0.0	0.0	**0.0**
$\mathbf{A^{Na}}$·C_6H_6	-0.7	-0.5	6.6	6.9	2.2	0.2	**3.1**
$\mathbf{B^{Na}}$	31.5	31.4	46.3	46.8	34.2	30.7	**33.4**
**TS(B-C)**$^{\mathbf{Na}}$	36.7	35.3	51.7	51.8	38.9	37.5	**39.8**
$\mathbf{C^{Na}}$	3.1	2.7	19.4	17.1	-0.1	5.2	**2.0**
$\mathbf{C^{Na}}$·C_6H_6	-12.6	-13.1	12.6	12.0	-14.3	-8.2	**-9.9**
$\mathbf{D^{Na}}$	25.6	24.0	50.4	47.6	20.5	26.8	**21.6**
**TS(D-E)**$^{\mathbf{Na}}$	31.7	28.1	56.5	53.1	31.7	31.9	**31.9**
$\mathbf{E^{Na}}$	-17.9	-20.0	8.7	4.5	-25.8	-14.9	**-22.8**
$\mathbf{D'^{Na}}$	5.3	5.2	37.0	37.6	-1.1	9.3	**3.0**
$\mathbf{F^{Na}}$	14.4	14.2	20.4	17.6	30.8	13.5	**29.9**
$\mathbf{G^{Na}}$	5.6	5.6	8.3	7.1	13.4	5.6	**13.5**
**TS(G-H)**$^{\mathbf{Na}}$	46.6	45.9	53.0	48.3	45.6	46.0	**45.0**
$\mathbf{H^{Na}}$	35.1	34.3	42.1	40.0	36.5	33.3	**34.7**
**TS(H-I)**$^{\mathbf{Na}}$	42.0	39.3	48.6	46.7	43.0	40.2	**41.1**
$\mathbf{I^{Na}}$	0.4	-0.5	8.2	4.0	-2.1	-0.8	**-3.3**
**TS(I-J)**$^{\mathbf{Na}}$	6.5	4.8	13.0	4.7	-2.3	5.7	**-3.2**
$\mathbf{J^{Na}}$	-5.2	-6.4	1.6	-1.7	-5.4	-6.1	**-6.3**
$\mathbf{K^{Na}}$	4.2	4.5	14.4	13.6	15.9	5.2	**16.9**
**TS(K-L)**$^{\mathbf{Na}}$	36.8	36.1	52.9	52.1	40.1	38.7	**42.0**
$\mathbf{L^{Na}}$	30.3	29.7	45.9	45.5	33.8	30.5	**34.0**
**TS(L-M)**$^{\mathbf{Na}}$	36.2	33.4	51.1	50.4	38.5	36.2	**38.5**
$\mathbf{M^{Na}}$	-8.9	-9.6	8.0	6.7	-9.4	-7.5	**-8.0**
$\mathbf{N^{Na}}$	-12.9	-13.8	3.2	2.5	-13.9	-10.6	**-11.6**

Table S4: Relative energies for computed structures (in kcal mol^{-1}). Data in bold is at the same level as free energies used in the text. Free energies are quoted relative to $\mathbf{A^K}$.

	ΔE_{BSI}	ΔH_{BSI}	ΔG_{BSI}	$\Delta G_{BSI/bnz}$	$\Delta G_{BSI/bnz+D3BJ}$	ΔE_{BS2}	ΔG_{bnz}
$\mathbf{A^K}$	0.0	0.0	0.0	0.0	0.0	0.0	**0.0**
$\mathbf{A^K}\cdot C_6H_6$	-0.8	-0.8	4.9	5.1	0.0	0.1	**0.9**
$\mathbf{B^K}$	37.5	36.9	51.1	50.1	32.3	34.6	**29.4**
**TS(B-C)^K**	41.0	38.9	53.8	52.6	36.0	39.1	**34.1**
$\mathbf{C^K}$	4.0	3.7	19.5	16.7	-2.1	4.2	**-1.9**
$\mathbf{C^K}\cdot C_6H_6$	-11.0	-11.9	12.3	11.1	-14.8	-9.0	**-12.9**
$\mathbf{D^K}$	18.9	17.8	45.1	44.8	8.9	17.7	**7.7**
**TS(D-E)^K**	34.3	30.0	57.2	52.3	18.1	31.3	**15.1**
$\mathbf{E^K}$	-18.1	-20.5	6.0	0.7	-33.4	-18.6	**-33.9**
$\mathbf{D'^K}$	8.5	7.9	37.3	36.8	-5.0	9.4	**-4.2**
$\mathbf{F^K}$	21.0	20.9	28.0	23.5	27.9	18.7	**25.7**
$\mathbf{G^K}$	9.7	9.4	10.06	7.6	14.5	8.9	**13.7**
**TS(G-H)^K**	47.5	46.7	53.7	48.1	42.0	44.2	**38.7**
$\mathbf{H^K}$	39.8	388.9	46.1	42.6	36.0	35.3	**31.5**
**TS(H-I)^K**	45.9	43.1	51.4	48.0	41.2	41.2	**36.6**
$\mathbf{I^K}$	2.1	1.0	8.5	3.5	-6.4	0.1	**-8.4**
**TS(I-J)^K**	2.1	1.0	10.1	5.3	-4.0	0.0	**-6.1**
$\mathbf{J^K}$	-3.5	-4.9	2.3	-2.3	-9.8	-6.1	**-12.4**
$\mathbf{K^K}$	7.5	7.8	19.5	18.2	11.3	8.5	**12.2**
**TS(K-L)^K**	39.6	39.2	55.4	52.8	41.1	38.7	**40.3**
$\mathbf{L^K}$	34.4	33.5	48.5	47.0	33.5	31.4	**30.5**
**TS(L-M)^K**	40.5	37.9	54.7	52.3	39.3	36.6	**35.3**
$\mathbf{M^K}$	-5.5	-6.4	9.5	6.6	-10.0	-5.7	**-10.3**
$\mathbf{N^K}$	-10.8	-11.8	3.7	1.7	-13.0	-11.0	**-13.2**

Table S5: Relative energies for computed structures (in kcal mol^{-1}). Data in bold is at the same level as free energies used in the text. Free energies are quoted relative to $\mathbf{A^{Rb}}$.

	ΔE_{BSI}	ΔH_{BSI}	ΔG_{BSI}	$\Delta G_{BSI/bnz}$	$\Delta G_{BSI/bnz+D3BJ}$	ΔE_{BS2}	ΔG_{bnz}
$\mathbf{A^{Rb}}$	0.0	0.0	0.0	0.0	0.0	0.0	**0.0**
$\mathbf{A^{Rb}}\cdot C_6H_6$	-0.8	-0.8	5.8	6.0	0.8	0.3	**1.9**
$\mathbf{B^{Rb}}$	38.7	37.9	51.4	50.2	30.4	36.1	**27.8**
$\mathbf{TS(B\text{-}C)^{Rb}}$	41.6	39.5	54.1	52.9	33.9	40.0	**32.3**
$\mathbf{C^{Rb}}$	3.6	3.2	18.9	16.1	-5. 3	4.1	**-4.8**
$\mathbf{C^{Rb}}\cdot C_6H_6$	-11.4	-12.5	11.1	9.6	-16.3	-9.0	**-13.9**
$\mathbf{D^{Rb}}$	29.1	26.9	51.9	48.0	11.4	28.1	**10.4**
$\mathbf{TS(D\text{-}E)^{Rb}}$	32.8	28.4	55.1	50.2	13.4	31.1	**11.7**
$\mathbf{E^{Rb}}$	-19.8	-22.4	3.2	-2.2	-39.7	-19.3	**-39.2**
$\mathbf{D'^{Rb}}$	8.7	7.9	36.9	36.0	-8.9	10.2	**-7.4**
$\mathbf{F^{Rb}}$	19.6	19.6	27.4	9.4	3.0	2.5	**21.9**
$\mathbf{G^{Rb}}$	9.9	9.8	11.1	7.8	13.5	9.2	**12.8**
$\mathbf{TS(G\text{-}H)^{Rb}}$	46.5	45.7	52.7	47.3	39.9	44.0	**37.4**
$\mathbf{H^{Rb}}$	39.9	38.8	45.2	41.6	33.7	36.4	**30.2**
$\mathbf{TS(H\text{-}I)^{Rb}}$	45.7	42.9	51.2	47.9	39.5	42.3	**36.1**
$\mathbf{I^{Rb}}$	4.7	0.3	6.6	0.9	-11.3	0.3	**-12.6**
$\mathbf{TS(I\text{-}J)^{Rb}}$	1.7	0.4	9.1	3.6	-9.1	0.6	**-10.2**
$\mathbf{J^{Rb}}$	-4.3	-5.8	0.9	-3.5	-13.0	-5.8	**-14.5**
$\mathbf{K^{Rb}}$	7.3	7.4	17.9	16.5	9.4	8.0	**10.1**
$\mathbf{TS(K\text{-}L)^{Rb}}$	39.8	39.3	54.8	51.9	39.5	39.1	**38.9**
$\mathbf{L^{Rb}}$	34.9	34.0	49.4	47.7	33.7	32.6	**31.3**
$\mathbf{TS(L\text{-}M)^{Rb}}$	40.9	38.3	54.4	51.8	37.9	38.1	**35.0**
$\mathbf{M^{Rb}}$	-5.2	-6.2	8.7	5.5	-11.9	-5.0	**-1.6**
$\mathbf{N^{Rb}}$	-10.7	-11.8	4.1	2.0	-13.4	-10.6	**-13.3**

Table S6: Relative energies for computed structures (in kcal mol^{-1}). Data in bold is at the same level as free energies used in the text. Free energies are quoted relative to $\mathbf{A^{Cs}}$.

	ΔE_{BSI}	ΔH_{BSI}	ΔG_{BSI}	$\Delta G_{BSI/bnz}$	$\Delta G_{BSI/bnz+D3BJ}$	ΔE_{BS2}	ΔG_{bnz}
$\mathbf{A^{Cs}}$	0.0	0.0	0.0	0.0	0.0	0.0	**0.0**
$\mathbf{A^{Cs}}\cdot C_6H_6$	-1.0	-0.7	5.6	5.7	1.7	-0.0	**2.7**
$\mathbf{B^{Cs}}$	38.4	37.8	51.2	49.9	30.1	34.8	**26.5**
**TS(B-C)**^{Cs}	41.4	39.2	52.8	51.7	31.2	39.0	**28.8**
$\mathbf{C^{Cs}}$	3.1	2.7	18.0	15.3	-7.2	3.3	**-7.1**
$\mathbf{C^{Cs}}\cdot C_6H_6$	-12.0	-12.9	10.4	8.9	-16.2	-10.0	**-14.1**
$\mathbf{D^{Cs}}$	26.8	24.9	51.7	47.5	11.3	25.4	**10.0**
**TS(D-E)**^{Cs}	29.9	25.9	53.0	49.0	12.3	28.0	**10.3**
$\mathbf{E^{Cs}}$	-22.5	-25.0	1.1	-3.4	-42.1	-22.0	**-41.7**
$\mathbf{D'^{Cs}}$	6.9	6.4	36.8	29.2	-18.8	8.2	**-17.6**
$\mathbf{F^{Cs}}$	18.2	18.3	26.8	23.1	20.9	16.1	**18.8**
$\mathbf{G^{Cs}}$	9.8	9.8	12.7	10.3	13.0	8.4	**11.5**
**TS(G-H)**^{Cs}	44.6	43.9	51.1	46.3	38.3	41.6	**35.3**
$\mathbf{H^{Cs}}$	38.7	37.8	44.5	41.7	33.7	34.3	**29.3**
**TS(H-I)**^{Cs}	44.3	41.6	50.1	47.4	38.7	40.1	**34.5**
$\mathbf{I^{Cs}}$	0.3	-1.0	5.8	1.5	-10.4	-1.3	**-11.9**
**TS(I-J)**^{Cs}	0.4	-0.8	8.0	3.6	-9.8	-1.0	**-11.2**
$\mathbf{J^{Cs}}$	-5.8	-7.3	-0.8	-4.4	-15.2	-7.5	**-16.9**
$\mathbf{K^{Cs}}$	7.0	7.1	18.4	17.0	10.9	6.8	**10.8**
**TS(K-L)**^{Cs}	39.1	38.7	53.6	50.4	38.5	37.7	**37.2**
$\mathbf{L^{Cs}}$	34.2	33.5	48.4	47.1	34.0	31.1	**30.8**
**TS(L-M)**^{Cs}	39.7	37.3	53.1	51.0	36.5	36.0	**32.8**
$\mathbf{M^{Cs}}$	-5.2	-5.9	9.9	7.1	-9.1	-5.4	**-9.4**
$\mathbf{N^{Cs}}$	-11.1	-12.1	2.7	0.6	-14.8	-11.4	**-15.1**

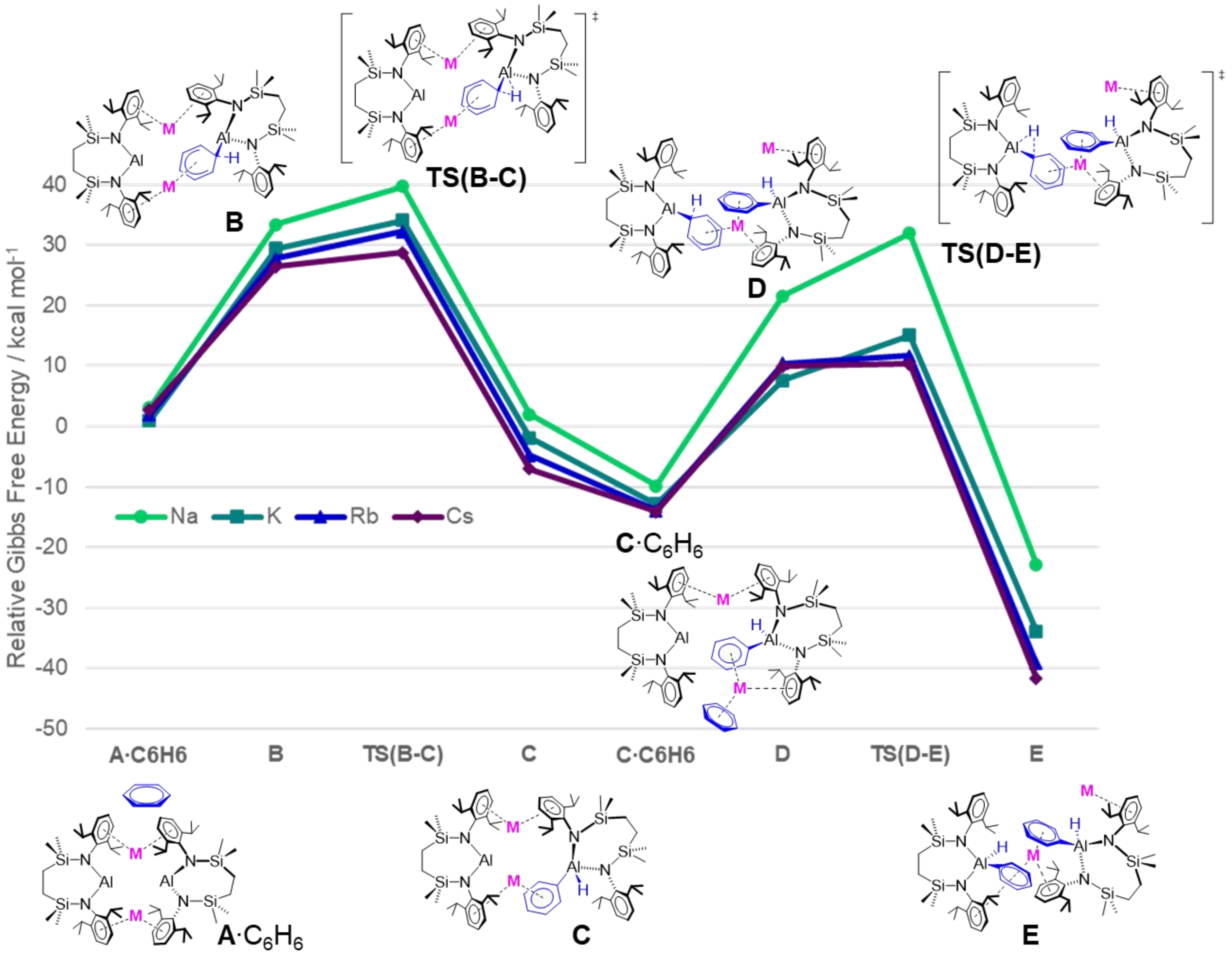

Figure S25: Computed free energy profile (BP86-D3BJ(PCM=C_6H_6)/BS2//BP86/BS1 level, energies quoted in kcal mol^{-1}) for the formation of the dimeric (hydrido)(phenyl)aluminate **E**, from **A**, for all four group 1 metals.

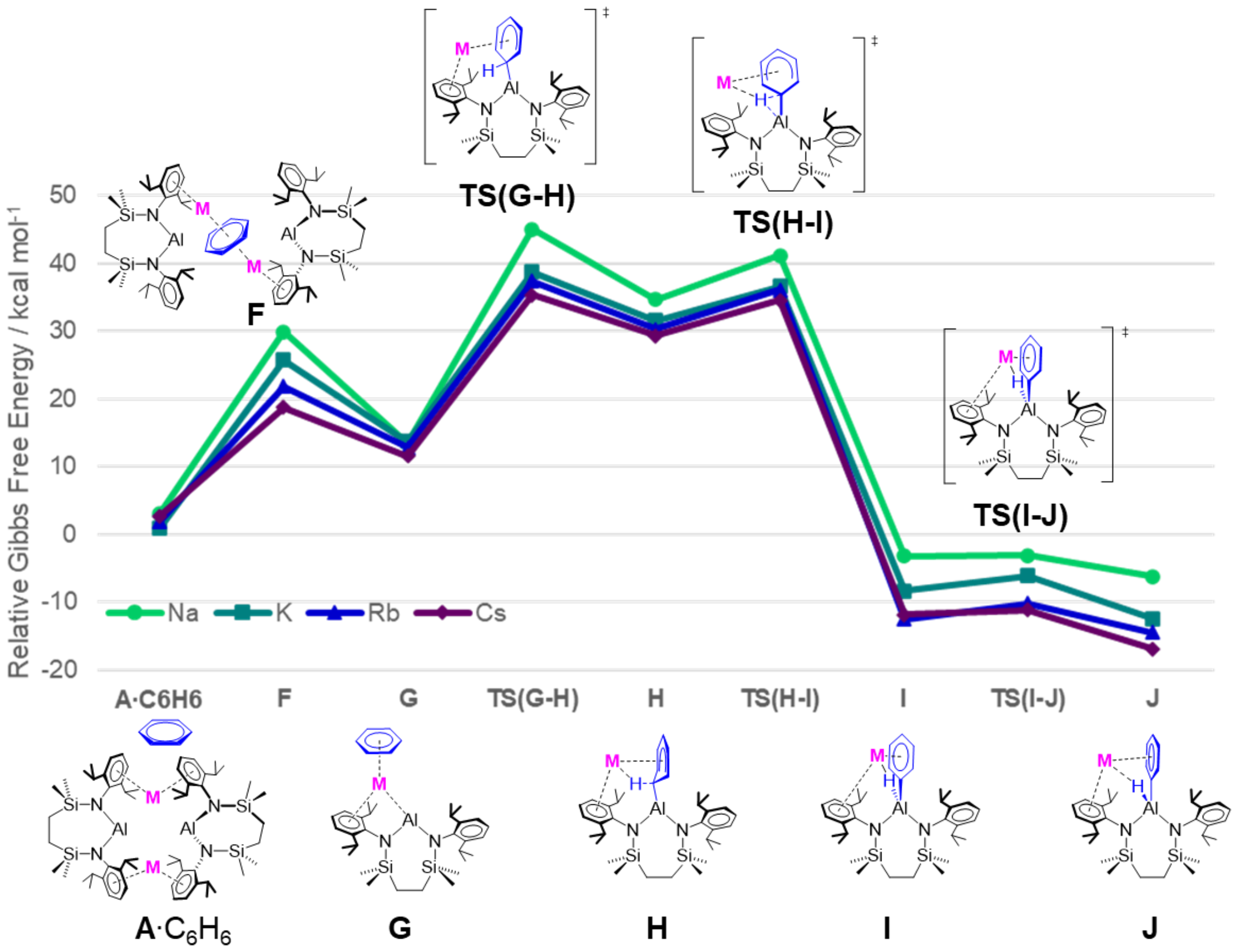

Figure S26: Computed free energy profile (BP86-D3BJ(PCM=C_6H_6)/BS2//BP86/BS1 level, energies quoted in kcal mol^{-1}) for the formation of the monomeric (hydrido)(phenyl)aluminate, **J**, from **A**, for all four group 1 metals.

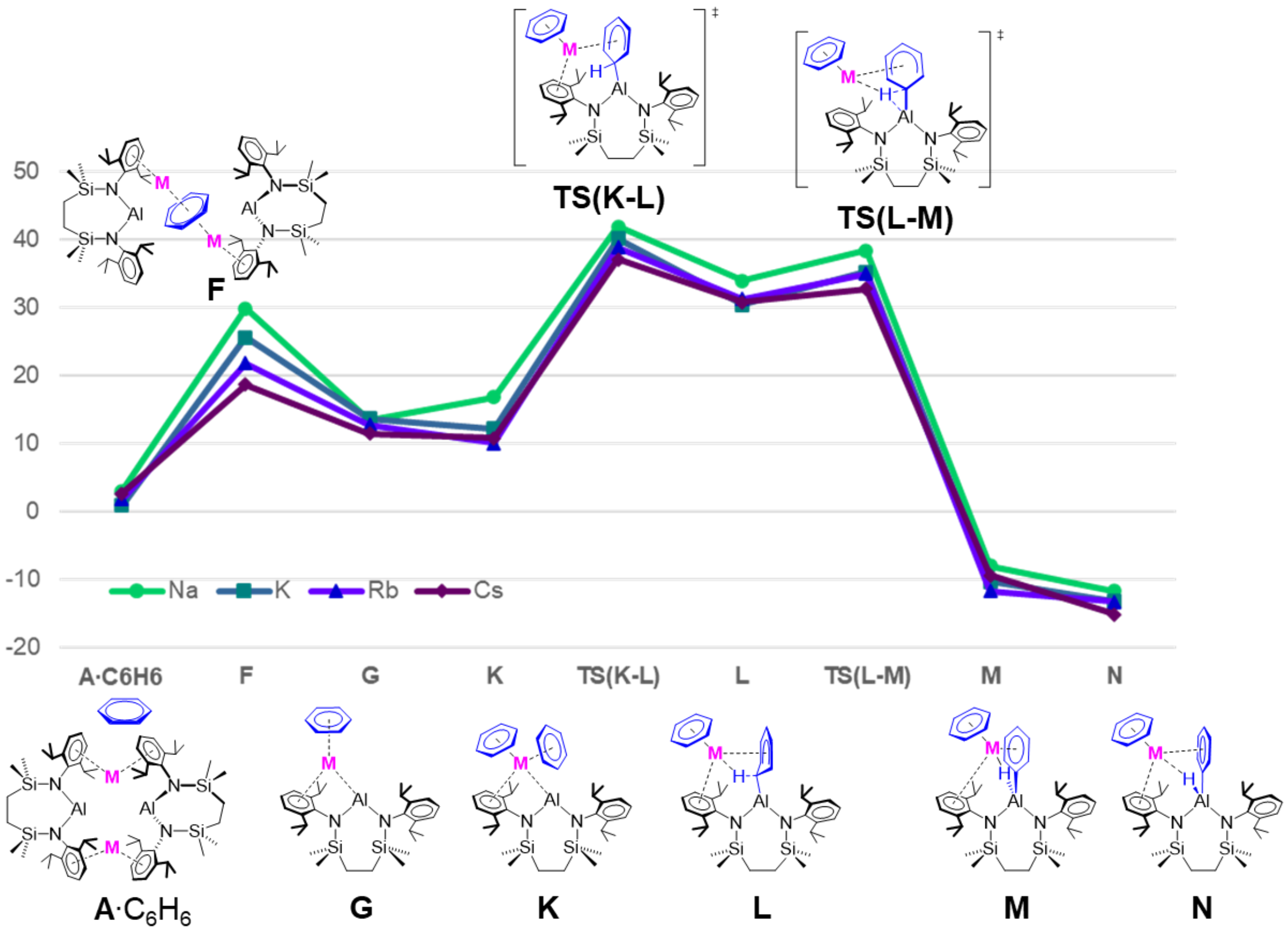

Figure S27. Computed free energy profile (BP86-D3BJ(PCM=C_6H_6)/BS2//BP86/BS1 level, energies quoted in kcal mol^{-1}) for the formation of the monomeric (hydrido)(phenyl)aluminate, **J**, from **A**, for all four group 1 metals, with a second benzene ring at the group 1 metal "M".

Raw Electronic Energies (in Hartrees)

C_6H_6

```
SCF (BP86) Energy = -232.242070159
Enthalpy 0K = -232.144365
Enthalpy 298K = -232.138889
Free Energy 298K = -232.171907
Lowest Frequency = 397.4324 cm-1
Second Frequency = 397.5218 cm-1
SCF (BP86-D3BJ) Energy = -232.258991628
SCF (C6H6) Energy = -232.243164330
SCF (BS2) Energy = -232.299995204
```

Na

A^{Na}
SCF (BP86) Energy = -2586.08782241
Enthalpy 0K = -2584.657168
Enthalpy 298K = -2584.559378
Free Energy 298K = -2584.796761
Lowest Frequency = 5.2428 cm-1
Second Frequency = 10.8198 cm-1
SCF (BP86-D3BJ) Energy = -2586.58341262
SCF (C6H6) Energy = -2586.09832536
SCF (BS2) Energy = -4534.23907213

A^{Na}·C_6H_6
SCF (BP86) Energy = -2818.33094366
Enthalpy 0K = -2816.802340
Enthalpy 298K = -2816.697424
Free Energy 298K = -2816.958192
Lowest Frequency = 5.2035 cm-1
Second Frequency = 7.2563 cm-1
SCF (BP86-D3BJ) Energy = -2818.85098555
SCF (C6H6) Energy = -2818.34195764
SCF (BS2) Energy = -4766.53870446

B^{Na}
SCF (BP86) Energy = -2818.27968567
Enthalpy 0K = -2816.751482
Enthalpy 298K = -2816.648736
Free Energy 298K = -2816.894917
Lowest Frequency = 7.2520 cm-1
Second Frequency = 10.2042 cm-1
SCF (BP86-D3BJ) Energy = -2818.81229890
SCF (C6H6) Energy = -2818.29051256
SCF (BS2) Energy = -4766.49007320

TS(B-C)^{Na}
SCF (BP86) Energy = -2818.27137927
Enthalpy 0K = -2816.745231
Enthalpy 298K = -2816.643458
Free Energy 298K = -2816.886299
Lowest Frequency = -682.7612 cm-1
Second Frequency = 6.4234 cm-1
SCF (BP86-D3BJ) Energy = -2818.80442987
SCF (C6H6) Energy = -2818.28274475
SCF (BS2) Energy = -4766.47926284

C^{Na}
SCF (BP86) Energy = -2818.32499117
Enthalpy 0K = -2816.797228
Enthalpy 298K = -2816.694973
Free Energy 298K = -2816.937736
Lowest Frequency = 8.3311 cm-1
Second Frequency = 12.9238 cm-1
SCF (BP86-D3BJ) Energy = -2818.86496753
SCF (C6H6) Energy = -2818.34020453
SCF (BS2) Energy = -4766.53081547

C^{Na}·C_6H_6
SCF (BP86) Energy = -3050.59201942
Enthalpy 0K = -3048.966781
Enthalpy 298K = -3048.857146
Free Energy 298K = -3049.120475
Lowest Frequency = 9.7258 cm-1
Second Frequency = 12.2425 cm-1
SCF (BP86-D3BJ) Energy = -3051.16335327
SCF (C6H6) Energy = -3050.60565578
SCF (BS2) Energy = -4998.85206270

D^{Na}
SCF (BP86) Energy = -3050.53110877
Enthalpy 0K = -3048.907729
Enthalpy 298K = -3048.799264
Free Energy 298K = -3049.060190
Lowest Frequency = 1.6703 cm-1
Second Frequency = 8.8556 cm-1
SCF (BP86-D3BJ) Energy = -3051.10380128
SCF (C6H6) Energy = -3050.54830210
SCF (BS2) Energy = -4998.79640167

TS(D-E)^{Na}
SCF (BP86) Energy = -3050.52143055
Enthalpy 0K = -3048.901155
Enthalpy 298K = -3048.793487
Free Energy 298K = -3049.050536
Lowest Frequency = -588.2954 cm-1
Second Frequency = 5.2050 cm-1
SCF (BP86-D3BJ) Energy = -3051.08494447
SCF (C6H6) Energy = -3050.53954715
SCF (BS2) Energy = -4998.78817574

E^{Na}
SCF (BP86) Energy = -3050.60049230
Enthalpy 0K = -3048.977795
Enthalpy 298K = -3048.869591
Free Energy 298K = -3049.126674
Lowest Frequency = 7.0789 cm-1
Second Frequency = 11.2875 cm-1
SCF (BP86-D3BJ) Energy = -3051.17822821
SCF (C6H6) Energy = -3050.61990075
SCF (BS2) Energy = -4998.86284422

D'^{Na}
SCF (BP86) Energy = -3050.56360096
Enthalpy 0K = -3048.937652
Enthalpy 298K = -3048.830590
Free Energy 298K = -3049.081693
Lowest Frequency = 6.4599 cm-1
Second Frequency = 13.1108 cm-1
SCF (BP86-D3BJ) Energy = -3051.15466765
SCF (C6H6) Energy = -3050.57524809
SCF (BS2) Energy = -4998.82418424

F^{Na}
SCF (BP86) Energy = -2818.30694876
Enthalpy 0K = -2816.778880
Enthalpy 298K = -2816.673868
Free Energy 298K = -2816.936184
Lowest Frequency = 2.4462 cm-1
Second Frequency = 4.6405 cm-1
SCF (BP86-D3BJ) Energy = -2818.79840933
SCF (C6H6) Energy = -2818.32296703
SCF (BS2) Energy = -4766.51755868

G^{Na}
SCF (BP86) Energy = -1525.27710011
Enthalpy 0K = -1524.464110
Enthalpy 298K = -1524.408934
Free Energy 298K = -1524.557068
Lowest Frequency = 10.4198 cm-1
Second Frequency = 11.1936 cm-1
SCF (BP86-D3BJ) Energy = -1525.53167356
SCF (C6H6) Energy = -1525.28540088
SCF (BS2) Energy = -2499.41062016

TS(G-H)^{Na}
SCF (BP86) Energy = -1525.21175239
Enthalpy 0K = -1524.399755
Enthalpy 298K = -1524.346208
Free Energy 298K = -1524.485838
Lowest Frequency = -96.6119 cm-1
Second Frequency = 10.9447 cm-1
SCF (BP86-D3BJ) Energy = -1525.48079683
SCF (C6H6) Energy = -1525.22554599
SCF (BS2) Energy = -2499.34620243

H^{Na}
SCF (BP86) Energy = -1525.23010925
Enthalpy 0K = -1524.418299
Enthalpy 298K = -1524.364704
Free Energy 298K = -1524.503244
Lowest Frequency = 19.5884 cm-1
Second Frequency = 23.0383 cm-1
SCF (BP86-D3BJ) Energy = -1525.50043650
SCF (C6H6) Energy = -1525.23975856
SCF (BS2) Energy = -2499.36653266

TS(H-I)^{Na}
SCF (BP86) Energy = -1525.21898144
Enthalpy 0K = -1524.410308
Enthalpy 298K = -1524.357455
Free Energy 298K = -1524.492910
Lowest Frequency = -556.1831 cm-1
Second Frequency = 19.1078 cm-1
SCF (BP86-D3BJ) Energy = -1525.48966905
SCF (C6H6) Energy = -1525.22827885
SCF (BS2) Energy = -2499.35550675

I^{Na}
SCF (BP86) Energy = -1525.28533717
Enthalpy 0K = -1524.473663
Enthalpy 298K = -1524.420434
Free Energy 298K = -1524.557297
Lowest Frequency = 19.7999 cm-1
Second Frequency = 25.3489 cm-1
SCF (BP86-D3BJ) Energy = -1525.55983486
SCF (C6H6) Energy = -1525.29827919
SCF (BS2) Energy = -2499.42073634

TS(I-J)^{Na}
SCF (BP86) Energy = -1525.27561996
Enthalpy 0K = -1524.465236
Enthalpy 298K = -1524.412324
Free Energy 298K = -1524.549536
Lowest Frequency = -18.2542 cm-1
Second Frequency = 10.6865 cm-1
SCF (BP86-D3BJ) Energy = -1525.55156558
SCF (C6H6) Energy = -1525.29521187
SCF (BS2) Energy = -2499.41052340

J^{Na}
SCF (BP86) Energy = -1525.29422675
Enthalpy 0K = -1524.483180
Enthalpy 298K = -1524.429763
Free Energy 298K = -1524.567757
Lowest Frequency = 15.0649 cm-1
Second Frequency = 21.8424 cm-1
SCF (BP86-D3BJ) Energy = -1525.56491365
SCF (C6H6) Energy = -1525.30576162
SCF (BS2) Energy = -2499.42917029

K^{Na}
SCF (BP86) Energy = -1757.52131073
Enthalpy 0K = -1756.610096
Enthalpy 298K = -1756.547927
Free Energy 298K = -1756.719237
Lowest Frequency = 3.8082 cm-1
Second Frequency = 4.3424 cm-1
SCF (BP86-D3BJ) Energy = -1757.79917738
SCF (C6H6) Energy = -1757.53010908
SCF (BS2) Energy = -2731.71129299

TS(K-L)^{Na}
SCF (BP86) Energy = -1757.46940747
Enthalpy 0K = -1756.559829
Enthalpy 298K = -1756.499336
Free Energy 298K = -1756.657917
Lowest Frequency = -90.3484 cm-1
Second Frequency = 8.9625 cm-1
SCF (BP86-D3BJ) Energy = -1757.77016542
SCF (C6H6) Energy = -1757.47813716
SCF (BS2) Energy = -2731.65785947

L^{Na}
SCF (BP86) Energy = -1757.47977584
Enthalpy 0K = -1756.570049
Enthalpy 298K = -1756.509398
Free Energy 298K = -1756.669005
Lowest Frequency = 6.3083 cm-1
Second Frequency = 16.1608 cm-1
SCF (BP86-D3BJ) Energy = -1757.78005567
SCF (C6H6) Energy = -1757.48784174
SCF (BS2) Energy = -2731.67098516

TS(L-M)^{Na}
SCF (BP86) Energy = -1757.47033389
Enthalpy 0K = -1756.564049
Enthalpy 298K = -1756.504144
Free Energy 298K = -1756.660821
Lowest Frequency = -563.3784 cm-1
Second Frequency = 5.5943 cm-1
SCF (BP86-D3BJ) Energy = -1757.77091498
SCF (C6H6) Energy = -1757.47886486
SCF (BS2) Energy = -2731.66181170

M^{Na}
SCF (BP86) Energy = -1757.54224034
Enthalpy 0K = -1756.632613
Enthalpy 298K = -1756.572472
Free Energy 298K = -1756.729437
Lowest Frequency = 6.1901 cm-1
Second Frequency = 12.4421 cm-1
SCF (BP86-D3BJ) Energy = -1757.84947157
SCF (C6H6) Energy = -1757.55179627
SCF (BS2) Energy = -2731.73146564

N^{Na}
SCF (BP86) Energy = -1757.54857684
Enthalpy 0K = -1756.639326
Enthalpy 298K = -1756.578949
Free Energy 298K = -1756.737044
Lowest Frequency = 8.3121 cm-1
Second Frequency = 11.4652 cm-1
SCF (BP86-D3BJ) Energy = -1757.85638547
SCF (C6H6) Energy = -1757.55712342
SCF (BS2) Energy = -2731.73646386

K

A^K
SCF (BP86) Energy = -2642.35511332
Enthalpy 0K = -2640.924883
Enthalpy 298K = -2640.827075
Free Energy 298K = -2641.063221
Lowest Frequency = 12.6225 cm-1
Second Frequency = 16.0088 cm-1
SCF (BP86-D3BJ) Energy = -2642.84199670
SCF (C6H6) Energy = -2642.36088469
SCF (BS2) Energy = -5409.62525030

A^K·C_6H_6
SCF (BP86) Energy = -2874.59842211
Enthalpy 0K = -2873.070493
Enthalpy 298K = -2872.965348
Free Energy 298K = -2873.227388
Lowest Frequency = 2.7778 cm-1
Second Frequency = 5.9414 cm-1
SCF (BP86-D3BJ) Energy = -2875.11022891
SCF (C6H6) Energy = -2874.60498543
SCF (BS2) Energy = -5641.92502057

B^K
SCF (BP86) Energy = -2874.53750781
Enthalpy 0K = -2873.010402
Enthalpy 298K = -2872.907398
Free Energy 298K = -2873.153668
Lowest Frequency = 8.4822 cm-1
Second Frequency = 11.3821 cm-1
SCF (BP86-D3BJ) Energy = -2875.06973447
SCF (C6H6) Energy = -2874.54594879
SCF (BS2) Energy = -5641.87014494

TS(B-C)^K
SCF (BP86) Energy = -2874.53193098
Enthalpy 0K = -2873.007232
Enthalpy 298K = -2872.904992
Free Energy 298K = -2873.149445
Lowest Frequency = -568.2323 cm-1
Second Frequency = 6.6554 cm-1
SCF (BP86-D3BJ) Energy = -2875.06223827
SCF (C6H6) Energy = -2874.54060944
SCF (BS2) Energy = -5641.86299724

C^K
SCF (BP86) Energy = -2874.59084518
Enthalpy 0K = -2873.063366
Enthalpy 298K = -2872.961133
Free Energy 298K = -2873.203982
Lowest Frequency = 7.6117 cm-1
Second Frequency = 12.5748 cm-1
SCF (BP86-D3BJ) Energy = -2875.12464305
SCF (C6H6) Energy = -2874.60225080
SCF (BS2) Energy = -5641.91851529

C^K·C_6H_6
SCF (BP86) Energy = -3106.85669862
Enthalpy 0K = -3105.232508
Enthalpy 298K = -3105.122537
Free Energy 298K = -3105.387411
Lowest Frequency = 7.3930 cm-1
Second Frequency = 13.8810 cm-1
SCF (BP86-D3BJ) Energy = -3107.41865052
SCF (C6H6) Energy = -3106.86657211
SCF (BS2) Energy = -5874.23964248

D^K
SCF (BP86) Energy = -3106.80915237
Enthalpy 0K = -3105.185303
Enthalpy 298K = -3105.076800
Free Energy 298K = -3105.335214
Lowest Frequency = 5.4968 cm-1
Second Frequency = 11.3412 cm-1
SCF (BP86-D3BJ) Energy = -3107.38707741
SCF (C6H6) Energy = -3106.81760974
SCF (BS2) Energy = -5874.19706814

TS(D-E)^K
SCF (BP86) Energy = -3106.78456657
Enthalpy 0K = -3105.165773
Enthalpy 298K = -3105.057466
Free Energy 298K = -3105.315883
Lowest Frequency = -571.8579 cm-1
Second Frequency = 6.9369 cm-1
SCF (BP86-D3BJ) Energy = -3107.35980123
SCF (C6H6) Energy = -3106.80036641
SCF (BS2) Energy = -5874.17536790

E^K
SCF (BP86) Energy = -3106.86802242
Enthalpy 0K = -3105.246205
Enthalpy 298K = -3105.137595
Free Energy 298K = -3105.397461
Lowest Frequency = 5.0291 cm-1
Second Frequency = 9.1342 cm-1
SCF (BP86-D3BJ) Energy = -3107.44300520
SCF (C6H6) Energy = -3106.88444689
SCF (BS2) Energy = -5874.25481177

D'^K
SCF (BP86) Energy = -3106.82566224
Enthalpy 0K = -3105.201001
Enthalpy 298K = -3105.093450
Free Energy 298K = -3105.347550
Lowest Frequency = 6.0585 cm-1
Second Frequency = 10.7250 cm-1
SCF (BP86-D3BJ) Energy = -3107.41299673
SCF (C6H6) Energy = -3106.83446460
SCF (BS2) Energy = -5874.21027621

F[K]
SCF (BP86) Energy = -2874.56379197
Enthalpy 0K = -2873.036014
Enthalpy 298K = -2872.931155
Free Energy 298K = -2873.190469
Lowest Frequency = 6.1750 cm-1
Second Frequency = 6.4303 cm-1
SCF (BP86-D3BJ) Energy = -2875.06061912
SCF (C6H6) Energy = -2874.57782538
SCF (BS2) Energy = -5641.89543403

G[K]
SCF (BP86) Energy = -1553.40415651
Enthalpy 0K = -1552.591828
Enthalpy 298K = -1552.536411
Free Energy 298K = -1552.686625
Lowest Frequency = 8.4939 cm-1
Second Frequency = 9.7906 cm-1
SCF (BP86-D3BJ) Energy = -1553.65351909
SCF (C6H6) Energy = -1553.41293040
SCF (BS2) Energy = -2937.09841820

TS(G-H)[K]
SCF (BP86) Energy = -1553.34394447
Enthalpy 0K = -1552.532466
Enthalpy 298K = -1552.478873
Free Energy 298K = -1552.617992
Lowest Frequency = -75.6653 cm-1
Second Frequency = 14.9502 cm-1
SCF (BP86-D3BJ) Energy = -1553.61392777
SCF (C6H6) Energy = -1553.35686172
SCF (BS2) Energy = -2937.04226920

H[K]
SCF (BP86) Energy = -1553.35624733
Enthalpy 0K = -1552.544855
Enthalpy 298K = -1552.491141
Free Energy 298K = -1552.629988
Lowest Frequency = 19.0325 cm-1
Second Frequency = 25.2212 cm-1
SCF (BP86-D3BJ) Energy = -1553.62702040
SCF (C6H6) Energy = -1553.36592682
SCF (BS2) Energy = -2937.05643855

TS(H-I)[K]
SCF (BP86) Energy = -1553.34647567
Enthalpy 0K = -1552.538198
Enthalpy 298K = -1552.485118
Free Energy 298K = -1552.621554
Lowest Frequency = -477.2901 cm-1
Second Frequency = 19.6960 cm-1
SCF (BP86-D3BJ) Energy = -1553.61766398
SCF (C6H6) Energy = -1553.35590165
SCF (BS2) Energy = -2937.04691520

I[K]
SCF (BP86) Energy = -1553.41631836
Enthalpy 0K = -1552.605173
Enthalpy 298K = -1552.551741
Free Energy 298K = -1552.689972
Lowest Frequency = 14.4935 cm-1
Second Frequency = 20.7514 cm-1
SCF (BP86-D3BJ) Energy = -1553.69241611
SCF (C6H6) Energy = -1553.42832917
SCF (BS2) Energy = -2937.11246546

TS(I-J)[K]
SCF (BP86) Energy = -1553.41627760
Enthalpy 0K = -1552.605159
Enthalpy 298K = -1552.552611
Free Energy 298K = -1552.687412
Lowest Frequency = -16.8365 cm-1
Second Frequency = 20.5598 cm-1
SCF (BP86-D3BJ) Energy = -1553.69148292
SCF (C6H6) Energy = -1553.42795870
SCF (BS2) Energy = -2937.11262161

J[K]
SCF (BP86) Energy = -1553.42521517
Enthalpy 0K = -1552.614639
Enthalpy 298K = -1552.561027
Free Energy 298K = -1552.699871
Lowest Frequency = 14.7595 cm-1
Second Frequency = 21.2730 cm-1
SCF (BP86-D3BJ) Energy = -1553.69762301
SCF (C6H6) Energy = -1553.43642271
SCF (BS2) Energy = -2937.12239586

K[K]
SCF (BP86) Energy = -1785.64970514
Enthalpy 0K = -1784.738690
Enthalpy 298K = -1784.676618
Free Energy 298K = -1784.844370
Lowest Frequency = 8.3134 cm-1
Second Frequency = 11.4005 cm-1
SCF (BP86-D3BJ) Energy = -1785.93805623
SCF (C6H6) Energy = -1785.65683307
SCF (BS2) Energy = -3169.39910738

TS(K-L)[K]
SCF (BP86) Energy = -1785.59855191
Enthalpy 0K = -1784.688676
Enthalpy 298K = -1784.628207
Free Energy 298K = -1784.787220
Lowest Frequency = -89.0422 cm-1
Second Frequency = 8.7919 cm-1
SCF (BP86-D3BJ) Energy = -1785.89437903
SCF (C6H6) Energy = -1785.60772717
SCF (BS2) Energy = -3169.35089529

L^K
SCF (BP86) Energy = -1785.60681015
Enthalpy 0K = -1784.697859
Enthalpy 298K = -1784.636973
Free Energy 298K = -1784.798101
Lowest Frequency = 7.3181 cm-1
Second Frequency = 10.3443 cm-1
SCF (BP86-D3BJ) Energy = -1785.90554285
SCF (C6H6) Energy = -1785.61434855
SCF (BS2) Energy = -3169.36253069

$TS(L-M)^K$
SCF (BP86) Energy = -1785.59708947
Enthalpy 0K = -1784.690731
Enthalpy 298K = -1784.630747
Free Energy 298K = -1784.788339
Lowest Frequency = -406.7795 cm-1
Second Frequency = 9.4953 cm-1
SCF (BP86-D3BJ) Energy = -1785.89509305
SCF (C6H6) Energy = -1785.60596916
SCF (BS2) Energy = -3169.35436636

M^K
SCF (BP86) Energy = -1785.67041997
Enthalpy 0K = -1784.761307
Enthalpy 298K = -1784.700925
Free Energy 298K = -1784.860322
Lowest Frequency = 7.7314 cm-1
Second Frequency = 9.4523 cm-1
SCF (BP86-D3BJ) Energy = -1785.97406451
SCF (C6H6) Energy = -1785.68014863
SCF (BS2) Energy = -3169.42176504

N^K
SCF (BP86) Energy = -1785.67893680
Enthalpy 0K = -1784.770042
Enthalpy 298K = -1784.709514
Free Energy 298K = -1784.869576
Lowest Frequency = 2.5851 cm-1
Second Frequency = 11.7784 cm-1
SCF (BP86-D3BJ) Energy = -1785.97951452
SCF (C6H6) Energy = -1785.68722098
SCF (BS2) Energy = -3169.43019566

Rb

A^{Rb}
SCF (BP86) Energy = -2633.94560140
Enthalpy 0K = -2632.515733
Enthalpy 298K = -2632.417698
Free Energy 298K = -2632.655716
Lowest Frequency = 12.7497 cm-1
Second Frequency = 14.5843 cm-1
SCF (BP86-D3BJ) Energy = -2634.42967725
SCF (C6H6) Energy = -2633.95114511
SCF (BS2) Energy = -4257.93276718

A^{Rb}·C_6H_6
SCF (BP86) Energy = -2866.18900701
Enthalpy 0K = -2864.661439
Enthalpy 298K = -2864.556128
Free Energy 298K = -2864.818380
Lowest Frequency = 6.2313 cm-1
Second Frequency = 8.2104 cm-1
SCF (BP86-D3BJ) Energy = -2866.69830180
SCF (C6H6) Energy = -2866.19530406
SCF (BS2) Energy = -4490.23237145

B^{Rb}
SCF (BP86) Energy = -2866.12595841
Enthalpy 0K = -2864.599672
Enthalpy 298K = -2864.496276
Free Energy 298K = -2864.745723
Lowest Frequency = 6.6492 cm-1
Second Frequency = 10.7599 cm-1
SCF (BP86-D3BJ) Energy = -2866.65842257
SCF (C6H6) Energy = -2866.13453388
SCF (BS2) Energy = -4490.17519264

TS(B-C)^{Rb}
SCF (BP86) Energy = -2866.12135863
Enthalpy 0K = -2864.597228
Enthalpy 298K = -2864.494699
Free Energy 298K = -2864.741399
Lowest Frequency = -524.8653 cm-1
Second Frequency = 6.3472 cm-1
SCF (BP86-D3BJ) Energy = -2866.65255484
SCF (C6H6) Energy = -2866.12996799
SCF (BS2) Energy = -4490.16908506

C^{Rb}
SCF (BP86) Energy = -2866.18192335
Enthalpy 0K = -2864.655018
Enthalpy 298K = -2864.552528
Free Energy 298K = -2864.797469
Lowest Frequency = 7.6714 cm-1
Second Frequency = 11.9832 cm-1
SCF (BP86-D3BJ) Energy = -2866.71703281
SCF (C6H6) Energy = -2866.19308165
SCF (BS2) Energy = -4490.22623352

C^{Rb}·C_6H_6
SCF (BP86) Energy = -3098.44786815
Enthalpy 0K = -3096.824321
Enthalpy 298K = -3096.714010
Free Energy 298K = -3096.981845
Lowest Frequency = 7.5272 cm-1
Second Frequency = 10.6297 cm-1
SCF (BP86-D3BJ) Energy = -3099.00698543
SCF (C6H6) Energy = -3098.45805405
SCF (BS2) Energy = -4722.54708417

D^{Rb}
SCF (BP86) Energy = -3098.38344028
Enthalpy 0K = -3096.761525
Enthalpy 298K = -3096.652522
Free Energy 298K = -3096.915163
Lowest Frequency = 5.1721 cm-1
Second Frequency = 7.3245 cm-1
SCF (BP86-D3BJ) Energy = -3098.95968094
SCF (C6H6) Energy = -3098.39910449
SCF (BS2) Energy = -4722.48797358

TS(D-E)^{Rb}
SCF (BP86) Energy = -3098.37750954
Enthalpy 0K = -3096.759239
Enthalpy 298K = -3096.650554
Free Energy 298K = -3096.911686
Lowest Frequency = -541.1637 cm-1
Second Frequency = 5.5547 cm-1
SCF (BP86-D3BJ) Energy = -3098.95422200
SCF (C6H6) Energy = -3098.39302061
SCF (BS2) Energy = -4722.48315511

E^{Rb}
SCF (BP86) Energy = -3098.46123465
Enthalpy 0K = -3096.840226
Enthalpy 298K = -3096.731126
Free Energy 298K = -3096.994454
Lowest Frequency = 3.1743 cm-1
Second Frequency = 8.3183 cm-1
SCF (BP86-D3BJ) Energy = -3099.03885882
SCF (C6H6) Energy = -3098.47754672
SCF (BS2) Energy = -4722.56349125

D'^{Rb}
SCF (BP86) Energy = -3098.41590128
Enthalpy 0K = -3096.791826
Enthalpy 298K = -3096.683922
Free Energy 298K = -3096.940733
Lowest Frequency = 6.0887 cm-1
Second Frequency = 10.0298 cm-1
SCF (BP86-D3BJ) Energy = -3099.00546618
SCF (C6H6) Energy = -3098.42500602
SCF (BS2) Energy = -4722.51655478

F^{Rb}
SCF (BP86) Energy = -2866.15642550
Enthalpy 0K = -2864.628905
Enthalpy 298K = -2864.523878
Free Energy 298K = -2864.783897
Lowest Frequency = 5.1229 cm-1
Second Frequency = 7.2652 cm-1
SCF (BP86-D3BJ) Energy = -2866.65758238
SCF (C6H6) Energy = -2866.17008047
SCF (BS2) Energy = -4490.20324451

G^{Rb}
SCF (BP86) Energy = -1549.19906545
Enthalpy 0K = -1548.386551
Enthalpy 298K = -1548.331199
Free Energy 298K = -1548.482128
Lowest Frequency = 6.0210 cm-1
Second Frequency = 8.2424 cm-1
SCF (BP86-D3BJ) Energy = -1549.44887285
SCF (C6H6) Energy = -1549.20816954
SCF (BS2) Energy = -2361.25168138

TS(G-H)^{Rb}
SCF (BP86) Energy = -1549.14075628
Enthalpy 0K = -1548.329402
Enthalpy 298K = -1548.275740
Free Energy 298K = -1548.415759
Lowest Frequency = -59.2134 cm-1
Second Frequency = 12.1390 cm-1
SCF (BP86-D3BJ) Energy = -1549.41150318
SCF (C6H6) Energy = -1549.15330274
SCF (BS2) Energy = -2361.19627018

H^{Rb}
SCF (BP86) Energy = -1549.15131367
Enthalpy 0K = -1548.340411
Enthalpy 298K = -1548.286418
Free Energy 298K = -1548.427672
Lowest Frequency = 14.6273 cm-1
Second Frequency = 19.7197 cm-1
SCF (BP86-D3BJ) Energy = -1549.42292676
SCF (C6H6) Energy = -1549.16094580
SCF (BS2) Energy = -2361.20834826

TS(H-I)^{Rb}
SCF (BP86) Energy = -1549.14210281
Enthalpy 0K = -1548.333941
Enthalpy 298K = -1548.280724
Free Energy 298K = -1548.418135
Lowest Frequency = -456.2114 cm-1
Second Frequency = 18.7345 cm-1
SCF (BP86-D3BJ) Energy = -1549.41435666
SCF (C6H6) Energy = -1549.15132366
SCF (BS2) Energy = -2361.19901115

I^{Rb}
SCF (BP86) Energy = -1549.21224902
Enthalpy 0K = -1548.401720
Enthalpy 298K = -1548.348000
Free Energy 298K = -1548.489262
Lowest Frequency = 6.4283 cm-1
Second Frequency = 16.2466 cm-1
SCF (BP86-D3BJ) Energy = -1549.49063041
SCF (C6H6) Energy = -1549.22512653
SCF (BS2) Energy = -2361.26587314

TS(I-J)^{Rb}
SCF (BP86) Energy = -1549.21211921
Enthalpy 0K = -1548.401605
Enthalpy 298K = -1548.348836
Free Energy 298K = -1548.485261
Lowest Frequency = -11.8122 cm-1
Second Frequency = 21.1351 cm-1
SCF (BP86-D3BJ) Energy = -1549.49138694
SCF (C6H6) Energy = -1549.22468888
SCF (BS2) Energy = -2361.26542359

J^{Rb}
SCF (BP86) Energy = -1549.22168362
Enthalpy 0K = -1548.411501
Enthalpy 298K = -1548.357612
Free Energy 298K = -1548.498396
Lowest Frequency = 12.1632 cm-1
Second Frequency = 19.6577 cm-1
SCF (BP86-D3BJ) Energy = -1549.49573357
SCF (C6H6) Energy = -1549.23245820
SCF (BS2) Energy = -2361.27567591

K^{Rb}
SCF (BP86) Energy = -1781.44536199
Enthalpy 0K = -1780.534840
Enthalpy 298K = -1780.472460
Free Energy 298K = -1780.643206
Lowest Frequency = 8.5649 cm-1
Second Frequency = 9.2568 cm-1
SCF (BP86-D3BJ) Energy = -1781.73256255
SCF (C6H6) Energy = -1781.45255571
SCF (BS2) Energy = -2593.55367605

TS(K-L)^{Rb}
SCF (BP86) Energy = -1781.39357743
Enthalpy 0K = -1780.483997
Enthalpy 298K = -1780.423311
Free Energy 298K = -1780.584272
Lowest Frequency = -83.6286 cm-1
Second Frequency = 7.6590 cm-1
SCF (BP86-D3BJ) Energy = -1781.68910671
SCF (C6H6) Energy = -1781.40329863
SCF (BS2) Energy = -2593.50403187

L^{Rb}
SCF (BP86) Energy = -1781.40129822
Enthalpy 0K = -1780.492396
Enthalpy 298K = -1780.431466
Free Energy 298K = -1780.592971
Lowest Frequency = 8.1765 cm-1
Second Frequency = 11.0757 cm-1
SCF (BP86-D3BJ) Energy = -1781.69956013
SCF (C6H6) Energy = -1781.40892677
SCF (BS2) Energy = -2593.51450419

TS(L-M)^{Rb}
SCF (BP86) Energy = -1781.39173128
Enthalpy 0K = -1780.485586
Enthalpy 298K = -1780.425363
Free Energy 298K = -1780.585012
Lowest Frequency = -355.3576 cm-1
Second Frequency = 6.2048 cm-1
SCF (BP86-D3BJ) Energy = -1781.68989417
SCF (C6H6) Energy = -1781.40074776
SCF (BS2) Energy = -2593.50573077

M^{Rb}
SCF (BP86) Energy = -1781.46520551
Enthalpy 0K = -1780.556405
Enthalpy 298K = -1780.495793
Free Energy 298K = -1780.657746
Lowest Frequency = 4.7248 cm-1
Second Frequency = 8.6138 cm-1
SCF (BP86-D3BJ) Energy = -1781.76875160
SCF (C6H6) Energy = -1781.47530870
SCF (BS2) Energy = -2593.57429313

N^{Rb}
SCF (BP86) Energy = -1781.47403236
Enthalpy 0K = -1780.565433
Enthalpy 298K = -1780.504727
Free Energy 298K = -1780.665095
Lowest Frequency = 9.9412 cm-1
Second Frequency = 10.8025 cm-1
SCF (BP86-D3BJ) Energy = -1781.77444299
SCF (C6H6) Energy = -1781.48240756
SCF (BS2) Energy = -2593.58323034

Cs

A^{Cs}
SCF (BP86) Energy = -2626.08290758
Enthalpy 0K = -2624.653689
Enthalpy 298K = -2624.555432
Free Energy 298K = -2624.795269
Lowest Frequency = 12.0088 cm-1
Second Frequency = 12.1241 cm-1
SCF (BP86-D3BJ) Energy = -2626.56582530
SCF (C6H6) Energy = -2626.08813663
SCF (BS2) Energy = -4250.07078859

A^{Cs}·C_6H_6
SCF (BP86) Energy = -2858.32656628
Enthalpy 0K = -2856.799229
Enthalpy 298K = -2856.693894
Free Energy 298K = -2856.958332
Lowest Frequency = 1.8028 cm-1
Second Frequency = 5.3149 cm-1
SCF (BP86-D3BJ) Energy = -2858.83277973
SCF (C6H6) Energy = -2858.33258690
SCF (BS2) Energy = -4482.37082448

B^{Cs}
SCF (BP86) Energy = -2858.26376081
Enthalpy 0K = -2856.737766
Enthalpy 298K = -2856.634294
Free Energy 298K = -2856.885570
Lowest Frequency = 3.8310 cm-1
Second Frequency = 10.5007 cm-1
SCF (BP86-D3BJ) Energy = -2858.79522199
SCF (C6H6) Energy = -2858.27217944
SCF (BS2) Energy = -4482.31526159

TS(B-C)^{Cs}
SCF (BP86) Energy = -2858.25903366
Enthalpy 0K = -2856.735575
Enthalpy 298K = -2856.632818
Free Energy 298K = -2856.883060
Lowest Frequency = -530.0217 cm-1
Second Frequency = 2.2164 cm-1
SCF (BP86-D3BJ) Energy = -2858.79156374
SCF (C6H6) Energy = -2858.26709750
SCF (BS2) Energy = -4482.30862233

C^{Cs}
SCF (BP86) Energy = -2858.32001076
Enthalpy 0K = -2856.793685
Enthalpy 298K = -2856.690972
Free Energy 298K = -2856.938508
Lowest Frequency = 6.7273 cm-1
Second Frequency = 8.6746 cm-1
SCF (BP86-D3BJ) Energy = -2858.85568652
SCF (C6H6) Energy = -2858.33070202
SCF (BS2) Energy = -4482.36551002

C^{Cs}·C_6H_6
SCF (BP86) Energy = -3090.58623005
Enthalpy 0K = -3088.962991
Enthalpy 298K = -3088.852615
Free Energy 298K = -3089.122474
Lowest Frequency = 4.9625 cm-1
Second Frequency = 8.6516 cm-1
SCF (BP86-D3BJ) Energy = -3091.14287398
SCF (C6H6) Energy = -3090.59612427
SCF (BS2) Energy = -4714.68671257

D^{Cs}
SCF (BP86) Energy = -3090.52440743
Enthalpy 0K = -3088.902766
Enthalpy 298K = -3088.793729
Free Energy 298K = -3089.056690
Lowest Frequency = 5.6747 cm-1
Second Frequency = 6.9801 cm-1
SCF (BP86-D3BJ) Energy = -3091.09891515
SCF (C6H6) Energy = -3090.53846865
SCF (BS2) Energy = -4714.63024643

TS(D-E)^{Cs}
SCF (BP86) Energy = -3090.51934934
Enthalpy 0K = -3088.901181
Enthalpy 298K = -3088.792479
Free Energy 298K = -3089.054626
Lowest Frequency = -509.1941 cm-1
Second Frequency = 5.1906 cm-1
SCF (BP86-D3BJ) Energy = -3091.09473521
SCF (C6H6) Energy = -3090.53306978
SCF (BS2) Energy = -4714.62612339

E^{Cs}
SCF (BP86) Energy = -3090.60286692
Enthalpy 0K = -3088.982199
Enthalpy 298K = -3088.872972
Free Energy 298K = -3089.137321
Lowest Frequency = 4.2477 cm-1
Second Frequency = 8.6586 cm-1
SCF (BP86-D3BJ) Energy = -3091.18138007
SCF (C6H6) Energy = -3090.61740007
SCF (BS2) Energy = -4714.70586309

D'^{Cs}
SCF (BP86) Energy = -3090.55598484
Enthalpy 0K = -3088.932223
Enthalpy 298K = -3088.824272
Free Energy 298K = -3089.080412
Lowest Frequency = 8.5546 cm-1
Second Frequency = 10.6991 cm-1
SCF (BP86-D3BJ) Energy = -3091.14928406
SCF (C6H6) Energy = -3090.57556413
SCF (BS2) Energy = -4714.65778830

F^{Cs}
SCF (BP86) Energy = -2858.29593806
Enthalpy 0K = -2856.768887
Enthalpy 298K = -2856.663763
Free Energy 298K = -2856.924514
Lowest Frequency = 5.5875 cm-1
Second Frequency = 7.0804 cm-1
SCF (BP86-D3BJ) Energy = -2858.79924707
SCF (C6H6) Energy = -2858.30814085
SCF (BS2) Energy = -4482.34518320

G^{Cs}
SCF (BP86) Energy = -1545.26783925
Enthalpy 0K = -1544.455536
Enthalpy 298K = -1544.400195
Free Energy 298K = -1544.549363
Lowest Frequency = 9.8108 cm-1
Second Frequency = 15.9524 cm-1
SCF (BP86-D3BJ) Energy = -1545.52201289
SCF (C6H6) Energy = -1545.27525391
SCF (BS2) Energy = -2357.32202650

TS(G-H)^{Cs}
SCF (BP86) Energy = -1545.21249301
Enthalpy 0K = -1544.401205
Enthalpy 298K = -1544.347462
Free Energy 298K = -1544.488103
Lowest Frequency = -69.7740 cm-1
Second Frequency = 12.6762 cm-1
SCF (BP86-D3BJ) Energy = -1545.48351520
SCF (C6H6) Energy = -1545.22394079
SCF (BS2) Energy = -2357.26912413

H^{Cs}
SCF (BP86) Energy = -1545.22189894
Enthalpy 0K = -1544.410994
Enthalpy 298K = -1544.356994
Free Energy 298K = -1544.498579
Lowest Frequency = 12.1067 cm-1
Second Frequency = 20.3200 cm-1
SCF (BP86-D3BJ) Energy = -1545.49305127
SCF (C6H6) Energy = -1545.23016745
SCF (BS2) Energy = -2357.28077702

TS(H-I)^{Cs}
SCF (BP86) Energy = -1545.21294057
Enthalpy 0K = -1544.404894
Enthalpy 298K = -1544.351592
Free Energy 298K = -1544.489763
Lowest Frequency = -399.6589 cm-1
Second Frequency = 18.5181 cm-1
SCF (BP86-D3BJ) Energy = -1545.48515507
SCF (C6H6) Energy = -1545.22096347
SCF (BS2) Energy = -2357.27147082

I^{Cs}
SCF (BP86) Energy = -1545.28310095
Enthalpy 0K = -1544.472770
Enthalpy 298K = -1544.418963
Free Energy 298K = -1544.560259
Lowest Frequency = 6.9130 cm-1
Second Frequency = 22.1399 cm-1
SCF (BP86-D3BJ) Energy = -1545.56029806
SCF (C6H6) Energy = -1545.29376490
SCF (BS2) Energy = -2357.33740950

TS(I-J)^{Cs}
SCF (BP86) Energy = -1545.28291193
Enthalpy 0K = -1544.472494
Enthalpy 298K = -1544.419676
Free Energy 298K = -1544.556825
Lowest Frequency = -12.6496 cm-1
Second Frequency = 15.3888 cm-1
SCF (BP86-D3BJ) Energy = -1545.56255887
SCF (C6H6) Energy = -1545.29366896
SCF (BS2) Energy = -2357.33701600

J^{Cs}
SCF (BP86) Energy = -1545.29270209
Enthalpy 0K = -1544.482814
Enthalpy 298K = -1544.428796
Free Energy 298K = -1544.570863
Lowest Frequency = 7.1198 cm-1
Second Frequency = 17.7124 cm-1
SCF (BP86-D3BJ) Energy = -1545.56827091
SCF (C6H6) Energy = -1545.30206479
SCF (BS2) Energy = -2357.34735164

K^{Cs}
SCF (BP86) Energy = -1777.51452553
Enthalpy 0K = -1776.604331
Enthalpy 298K = -1776.541906
Free Energy 298K = -1776.712181
Lowest Frequency = 6.7876 cm-1
Second Frequency = 11.6485 cm-1
SCF (BP86-D3BJ) Energy = -1777.79950542
SCF (C6H6) Energy = -1777.52153219
SCF (BS2) Energy = -2589.62449588

TS(K-L)^{Cs}
SCF (BP86) Energy = -1777.46327673
Enthalpy 0K = -1776.553983
Enthalpy 298K = -1776.493099
Free Energy 298K = -1776.656092
Lowest Frequency = -87.3896 cm-1
Second Frequency = 6.6342 cm-1
SCF (BP86-D3BJ) Energy = -1777.75755656
SCF (C6H6) Energy = -1777.47306995
SCF (BS2) Energy = -2589.57525547

L^{Cs}
SCF (BP86) Energy = -1777.47107049
Enthalpy 0K = -1776.562234
Enthalpy 298K = -1776.501190
Free Energy 298K = -1776.664351
Lowest Frequency = 6.1613 cm-1
Second Frequency = 7.7833 cm-1
SCF (BP86-D3BJ) Energy = -1777.76737275
SCF (C6H6) Energy = -1777.47787400
SCF (BS2) Energy = -2589.58586453

TS(L-M)^{Cs}
SCF (BP86) Energy = -1777.46230668
Enthalpy 0K = -1776.556062
Enthalpy 298K = -1776.495860
Free Energy 298K = -1776.656853
Lowest Frequency = -362.7109 cm-1
Second Frequency = 2.7439 cm-1
SCF (BP86-D3BJ) Energy = -1777.76080978
SCF (C6H6) Energy = -1777.47040103
SCF (BS2) Energy = -2589.57797581

M^{Cs}
SCF (BP86) Energy = -1777.53386110
Enthalpy 0K = -1776.624973
Enthalpy 298K = -1776.564381
Free Energy 298K = -1776.725754
Lowest Frequency = 6.5479 cm-1
Second Frequency = 8.2597 cm-1
SCF (BP86-D3BJ) Energy = -1777.83501104
SCF (C6H6) Energy = -1777.54302548
SCF (BS2) Energy = -2589.64403871

N^{Cs}
SCF (BP86) Energy = -1777.54331599
Enthalpy 0K = -1776.634877
Enthalpy 298K = -1776.574082
Free Energy 298K = -1776.737206
Lowest Frequency = 1.9718 cm-1
Second Frequency = 6.9991 cm-1
SCF (BP86-D3BJ) Energy = -1777.84313146
SCF (C6H6) Energy = -1777.55143325
SCF (BS2) Energy = -2589.65357394

References

1. Schwamm, R. J.; Coles, M. P.; Hill, M. S.; Mahon, M. F.; McMullin, C. L.; Rajabi, N. A.; Wilson, A. S. S., A Stable Calcium Alumanyl. *Angew. Chem. Int. Ed.* **2020**, *59*, 3928-3932.
2. Dolomanov, O. V.; Bourhis, L. J.; Gildea, R. J.; Howard, J. A. K.; Puschmann, H. *J. Appl. Cryst.* **2009**, *42*, 339-341.
3. Sheldrick, G. M. *Acta Cryst*. **2015**, A71, 3-8.
4. Sheldrick, G. M. *Acta Cryst*. **2015**, C71, 3-8.
5. M. J. Frisch, G. W. Trucks, H. B. Schlegel, G. E. Scuseria, M. A. Robb, J. R. Cheeseman, G. Scalmani, V. Barone, G. A. Petersson, H. Nakatsuji, X. Li, M. Caricato, A. V. Marenich, J. Bloino, B. G. Janesko, R. Gomperts, B. Mennucci, H. P. Hratchian, J. V. Ortiz, A. F. Izmaylov, J. L. Sonnenberg, Williams, F. Ding, F. Lipparini, F. Egidi, J. Goings, B. Peng, A. Petrone, T. Henderson, D. Ranasinghe, V. G. Zakrzewski, J. Gao, N. Rega, G. Zheng, W. Liang, M. Hada, M. Ehara, K. Toyota, R. Fukuda, J. Hasegawa, M. Ishida, T. Nakajima, Y. Honda, O. Kitao, H. Nakai, T. Vreven, K. Throssell, J. A. Montgomery Jr., J. E. Peralta, F. Ogliaro, M. J. Bearpark, J. J. Heyd, E. N. Brothers, K. N. Kudin, V. N. Staroverov, T. A. Keith, R. Kobayashi, J. Normand, K. Raghavachari, A. P. Rendell, J. C. Burant, S. S. Iyengar, J. Tomasi, M. Cossi, J. M. Millam, M. Klene, C. Adamo, R. Cammi, J. W. Ochterski, R. L. Martin, K. Morokuma, O. Farkas, J. B. Foresman, D. J. Fox, Wallingford, CT, 2016.
6. Andrae, D.; Häußermann, U.; Dolg, M.; Stoll, H.; Preuß, H. *Theor. Chim. Acta* **1990**, *77*, 123-141.
7. (a) Hariharan, P. C.; Pople, J. A. *Theor. Chim. Acta* **1973**, 28, 213-222; (b) Hehre, W. J.; Ditchfield, R.; Pople, J. A.; *J. Chem. Phys.* **1972**, *56*, 2257-2261.
8. (a) Becke, A. D.; *Phys. Rev. A* **1988**, *38*, 3098-3100; (b) Perdew, J. P. *Phys. Rev. B* **1986**, *33*, 8822-8824.
9. Tomasi, J.; Mennucci, B.; Cammi, R. *Chem. Rev.* **2005**, *105*, 2999-3094.
10. Grimme, S.; Ehrlich, S.; Goerigk, L. *J. Comp. Chem.* **2011**, *32*, 1456-1465.
